# Selective photothermal killing of cancer cells using LED-activated nucleus targeting fluorescent carbon dots†

**DOI:** 10.1039/c9na00293f

**Published:** 2019-07-15

**Authors:** Stephen A. Hill, Sadiyah Sheikh, Qiaoyu Zhang, Lorena Sueiro Ballesteros, Andrew Herman, Sean A. Davis, David J. Morgan, Monica Berry, David Benito-Alifonso, M. Carmen Galan

**Affiliations:** School of Chemistry, University of Bristol Cantock's Close Bristol UK M.C.Galan@bristol.ac.uk; School of Cellular and Molecular Medicine, Faculty of Life Sciences Flow Cytometry Facility University Walk Bristol UK; Cardiff Catalysis Institute, School of Chemistry, Cardiff University Park Place Cardiff UK

## Abstract

The development of effective theranostic probes in cancer therapy is hampered due to issues with selectivity and off-target toxicity. We report the selective LED-photothermal ablation of cervical (HeLa) cancer cells over human dermal fibroblasts (HDF) using a new class of green-emissive fluorescent carbon dots (FCDs). The FCDs can be easily prepared in one pot using cheap and commercial starting materials. Physico-chemical characterization revealed that a surface coating of 2,5-deoxyfructosazine on a robust amorphous core gives rise to the nanomaterial's unique properties. We show that intracellular uptake mostly involves passive mechanisms in combination with intracellular DNA interactions to target the nucleus and that cancer cell selective killing is likely due to an increase in intracellular temperature in combination with ATP depletion, which is not observed upon exposure to either the “naked” core FCDs or the surface components individually. The selectivity of these nanoprobes and the lack of apparent production of toxic metabolic byproducts make these new nanomaterials promising agents in cancer therapy.

## Abstract 2

Effective theranostic probes in cancer therapy are still a challenge. We report the selective killing of cancer cells upon photothermal ablation using a new class of 2,5-deoxyfructosazine-coated green-emissive carbon dots using mild LED-irradiation.

## Graphical abstract

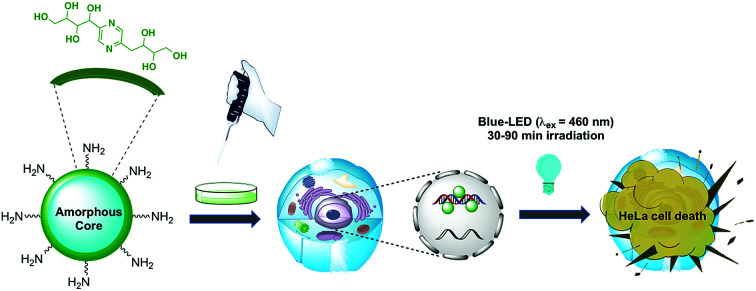

Photothermal therapy (PTT) is a promising non-invasive therapeutic strategy, in which nanoparticles embedded within tumors generate heat, typically in response to exogenously applied light, for thermal ablation of cancer cells.^1–3^ PTT offers unique advantages in cancer therapy including high specificity, minimal invasiveness and precise spatial–temporal selectivity.^4^ A variety of photothermal nanotherapeutics (PTN) including noble metal nanostructures, nanocarbons, carbon dots, transition metal sulfide/oxide nanomaterials, and organic nano-agents have been extensively explored with encouraging results.^2,5–9^ However, one of the major challenges in PTT is the ability to selectively and efficiently find a biocompatible material that can target cancerous cells while avoiding non-specific heating of healthy cells upon irradiation and that does not generate toxic byproducts.^5,10^

Successful cancer therapy relies on early and accurate diagnosis, and fluorescence imaging has increasingly been recognized as a viable strategy to identify a range of cancers.^11,12^ Fluorescent probes that exhibit high stability, sensitivity and specificity for their target without the limitations of organic fluorophores and fluorescent proteins are of great interest in many areas of research, particularly in cellular biology, bioimaging and medical diagnostics.^13,14^ Nanomaterials with novel optical, electronic and surface properties, have become useful platforms for a myriad of applications including imaging, drug delivery and diagnostics.^15–17^ Fluorescent carbon dots (FCDs)^18–22^ have emerged as a new class of non-isotopic detection labels suitable for live cell imaging, that provide a non-toxic alternative to heavy metal-containing fluorescent nanomaterials (*e.g.* quantum dots) while offering many advantages with regard to their ease and low cost of synthesis, unique photoluminescence (PL) properties, chemical inertness, high water solubility and generally low cytotoxicity.^23–29^

Previous work by our group^28–34^ and others^35–38^ on the use of water soluble fluorescent probes for live cell imaging applications has shown that the nanoparticle type, size, shape and surface functionalization have a significant effect on their intracellular uptake and localization. Encouraged by this, we embarked on the synthesis of a new class of fluorescent carbon-based nanomaterials (FCD-3) with unique surface functionality that could be used to target cancer cells and have potential applications in PTT.

Initial efforts were devoted to preparing a green-emitting nanomaterial that could be more easily imaged directly in live cells using confocal microscopy than the more common blue-emitting FCDs.^23,26,39–42^ The synthesis of FCD-3 was thus achieved in one pot after three minutes of microwave heating (domestic 700 W MW) of glucosamine·HCl 1 and *m*-phenylenediamine 2 (Scheme 1A, see the ESI† for experimental details).^43^ Centrifugal filtration (10 kDa molecular weight cut-off) afforded monodisperse quasi-spherical FCD-3. The nanoparticles exhibited an average diameter of 2.42 ± 0.55 nm (Fig. 1A) and an amorphous, carbonaceous core as determined by high-resolution transmission electron microscopy (HR-TEM, Fig. S2†). Dynamic light scattering (DLS) indicated a hydrodynamic diameter of 6–9 nm (Fig. S1A†), while the zeta (electrokinetic) potential indicated a cationic surface of around 12 mV (Fig. S1B†). Thermogravimetric analysis (TGA) showed good core stability at high temperatures, while residual solvent and surface-adhered species were more loosely associated (95% of the mass of FCD-3 was retained when heating to 150 °C, while a 65% mass loss was recorded when reaching 650 °C, see Fig. S3†). Fluorescence spectroscopy showed an excitation-independent emission for FCD-3 centered around 525 nm (*λ*_ex_ = 350–500 nm, Fig. 1B), indicative of emissive center uniformity. The quantum yield for FCD-3 was calculated to be 33% relative to fluorescein (Fig. S4 in the ESI†) and shown to be photostable upon continuous 60 min irradiation with no significant decrease in emission intensity (*λ*_ex_ = 460 nm, *λ*_em_ = 525 nm, Fig. S5 and S6†).^44^ The photoluminescence (PL) properties remained unchanged in the presence of a different range of potential metal and organic quenchers (500 μM), confirming the stability of FCD-3 (see Fig. S7†).

**Scheme 1:**
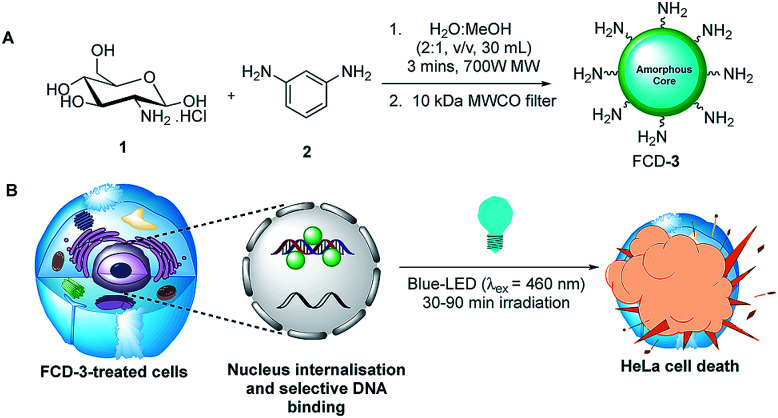
(A) Three minute synthesis of green emitting FCD-3 and (B) FCD-3 nuclear targeting leading to photothermal cancer cell ablation after blue-LED irradiation.

**Fig. 1:**
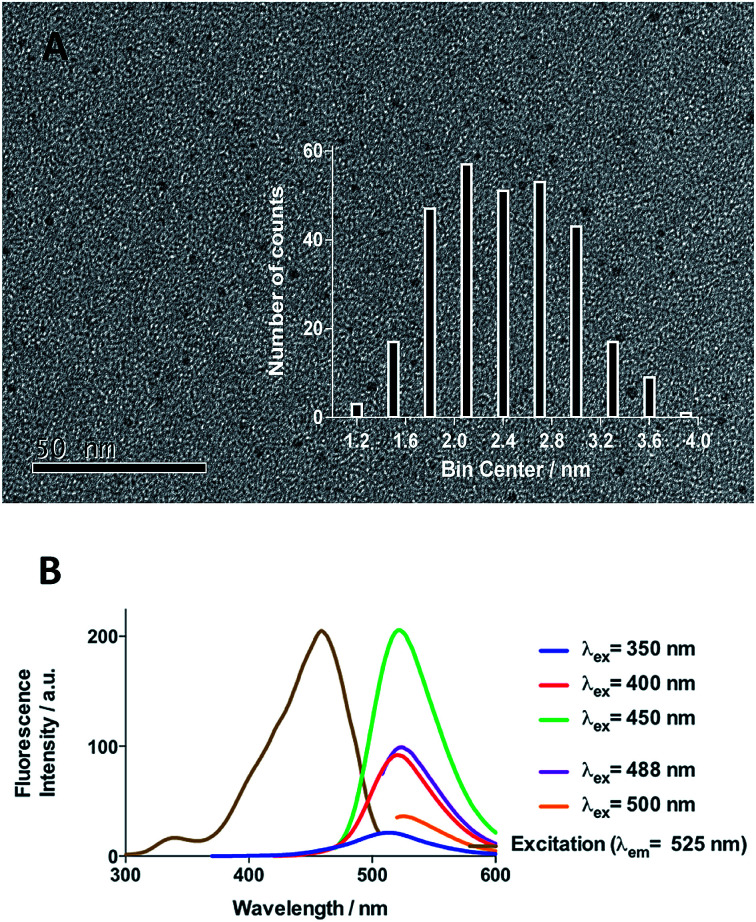
(A) TEM image of FCD-3 and distribution of diameters between 1.8 and 3 nm. (B) Fluorescence emission and excitation spectra showing that fluorescence emission is excitation independent.

To ascertain the chemical composition of FCD-3, functional group analysis was conducted using Fourier-transformed infra-red (FTIR) spectroscopy, X-ray photoelectron spectroscopy (XPS) and UV-vis spectroscopy. The FTIR signal at 3338 cm^−1^ was attributed to either residual carbohydrate architecture (OH groups) or N–H bonds (Fig. S8†). Surface passivation was evidenced by the IR stretching at 1629 cm^−1^ (C=O amide) and minor peaks at 1016 cm^−1^ (C–O or C–N functionality), while the broad signal at 612 cm^−1^ indicated C–Cl bonds. The presence of mainly C, H, N, Cl and O in FCD-3 was also confirmed by elemental and XPS analysis (Table S1 and Fig. S9†). Peak deconvolution of each element indicated the likely presence of a mixture of functionalities, *e.g.* for O, aliphatic hydroxyl/ether C–O or ester/imidate O*–C=O/N; aromatic OH or C=O; for N, amine/N-heteroaromatic motifs or N–C=O bridged structures; for C, C–C and C–OH/C–O–C/C–N bonds and minor peaks as C=O and O–C=O species and for Cl, R–NH_3_·Cl and C–Cl motifs (Fig. S10 and Table S2†). Additionally, UV-vis spectroscopy showed well-defined peaks at 212 nm, 260 nm (aromatic π–π* transitions), and 370 nm (n–π* aromatic C=C or C=O/C=N, Fig. S11†), further confirming the presence of key functional groups.^45,46^

NMR analysis was used to shed further light on the molecular architectures found on FCD-3 (Fig. S12†). Pyrazine motifs were identified (three singlets at *δ* 8.51, 8.30 and 5.01 ppm),^47^ as probed by ^1^H, ^1^H–^13^C HSQC and HMBC experiments, in addition to other complex N-hetero/aromatics akin to fructosazine and likely generated from 1,2-aminoaldose self-dimerisation^48^ (Fig. S13 and S14†). Polyhydroxylated species (*δ* 2.50–4.00 ppm), arising from incomplete glycan dehydration,^49^ were also detected. Time-point ^1^H and ^13^C NMR analysis of reaction aliquots carried out at 30 s intervals up to 180 s (Fig. S15 and S16 in the ESI†) showed loss of sugar anomeric signals from the starting carbohydrate in the spectra, indicative of pyranose ring-opening and iminium formation, followed by dehydration.^29^ Other aromatic structures were also detected in the spectra (*δ* 6.00–7.50 ppm) suggesting the presence of complex polyaromatics. Indeed, ^1^H–^13^C HMBC analysis demonstrated the presence of sp^2^ and sp^3^-enriched surface domains.

Since nanoparticle surface composition is important for biological recognition, additional efforts were made to fully identify the surface molecular structures found on FCD-3. The material was thus subjected to Sephadex G15 size exclusion chromatography using methanol as the polar solvent to help separate non-covalently bound surface species from the core-FCD. In this manner, green fluorescent 2,5-deoxyfructosazine 4, which is a known product of glucosamine self-condensation,^50,51^ was isolated as the major component in addition to core FCD-5 (FCD without 4) and FCD-3, as determined by ^1^H-NMR of the fractions (Fig. S17–S20† and Scheme 2).^52^ Zeta potential measurements of core 5 (−1.37) *vs.*3 (12.05) showed a change from a slightly negative net charge to a cationic charge when 4 is conjugated to the naked core, as expected for a passivated surface. Moreover, diffusion ordered spectroscopy (DOSY) of FCD-3 showed that it had a single diffusion coefficient of 3.83 × 10^−6^ cm^2^ s^−1^ confirming that 4 is associated with the FCD-core in FCD-3 (Fig. S20†).

**Scheme 2:**
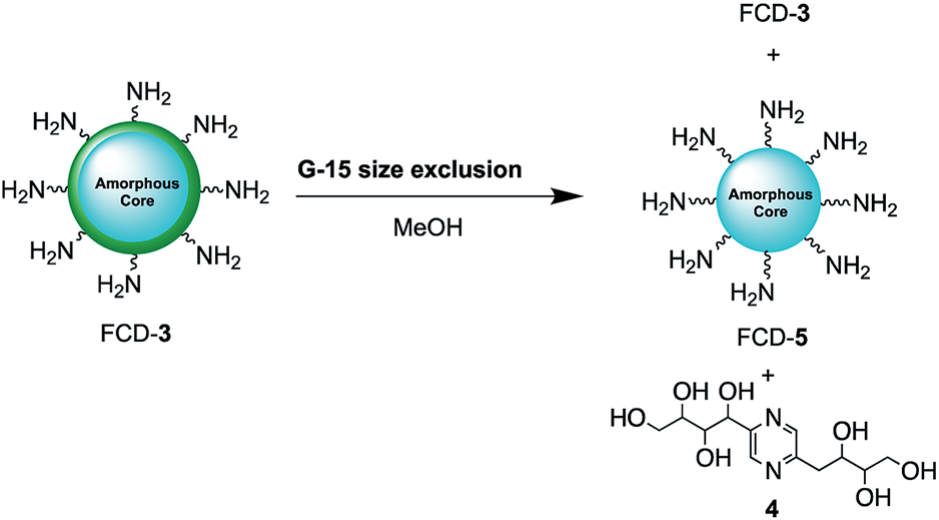
2,5-Deoxyfructosazine 4 was isolated from FCD-3 after size exclusion chromatography.

The utility of FCD-3 as a bioimaging tool was then investigated in HeLa cells (cervical cancer) and human dermal fibroblasts (HDF). It was found that FCD-3 (50 μg mL^−1^) was internalized in both cell lines, although the rate of uptake and intracellular localization differed between cell lines. Both active and passive cell transport mechanisms (37 °C *vs.* 4 °C) were observed, the latter accounting for 67% of fluorescence in HeLa, (Fig. S21†) but only 20% in HDF (Fig. S24 and S25†). Interestingly, very rapid nuclear internalization of FCD-3 was observed for HeLa cells within 1 min (Fig. S22†), with maximum internalization at 5 min after which nuclear fluorescence decreased slightly due to increased cytosolic accumulation which plateaued after 24 h with an average Global Pearson Coefficient (GPC) = 0.62 at 2 h exposure (Fig. 2 and 3). No significant colocalization of FCD-3 was observed for any other organelle (for representative images see Fig. S29–S36†). This result is significant as previous glucosamine-based blue-emitting FCDs^26,29,33^ did not target the cell nucleus. In contrast, much slower overall cellular uptake of FCD-3 was detected for healthy HDF cells (Fig. S26 and S27†) with colocalization in the nucleus only seen after 2 h of continuous exposure (GPC = 0.50, Fig. S28†). Similarly, only nuclear and cytosolic accumulation was detected. These results might be linked to the different cell metabolic ratios associated with cancer *vs.* non-cancer cell lines.^53^

**Fig. 2:**
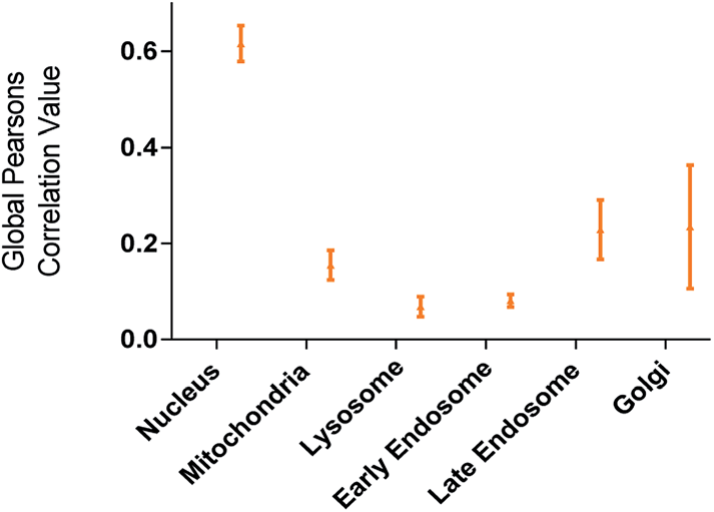
Colocalisation values (average of global Pearson's correlation) for FCD-3 treated HeLa cells (50 μg mL^−1^ after 2 h at 37 °C) with different organelle-selective fluorescent trackers.

**Fig. 3:**
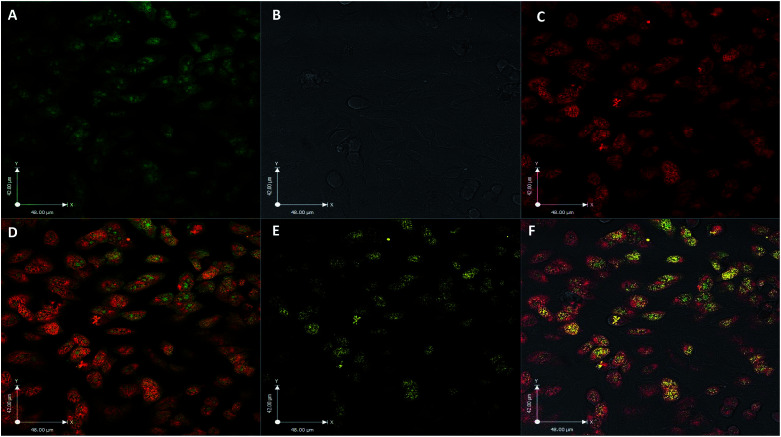
Confocal fluorescence images of HeLa cells after exposure to FCD-3 (50 μg mL^−1^, 2 h). (A) FCD-3 (green); (B) bright field; (C) nucleus tracker (NucRed647); (D) colocalization of FCD-3 and NucRed647 (yellow), (E) positive colocalization in yellow and (F) overlay of all channels.

The nucleus contains most of the cell genetic material *e.g.* DNA, RNA, rRNA and proteins.^54^ Thus, it was proposed that favorable electrostatic interactions between FCD-3 and the phosphate backbone of polynucleotide chains might be responsible for the nuclear uptake observed. To evaluate this, cells were incubated with FCD-3 for 2 h at 37 °C as before (see the ESI† for experimental details), before Förster resonance energy transfer (FRET) between FCD-3 (FRET donor) and cell permeable DNA (NuclearID-Red^55^ and DRAQ-5 (ref. 56)) or RNA (F22 RNASelect^57^) intercalating dyes as FRET acceptors was measured using flow cytometry (Fig. S37–S43†). For both cell lines, a FRET-associated decrease in emission was observed between FCD-3 and intercalating DNA-dyes. For instance, in HeLa cells FCD-3 donor emission decreased by 82% and 60% with Enzo NuclearID-Red and DRAQ-5 DNA dyes, respectively (Fig. S40 and S41†), and by 50% and 44% in HDF (Fig. S41 and S42†).^58^ On the other hand, no RNA FRET-response was observed with F22 in HeLa and a minor reduction of 7% was seen in HDF (Fig. S40 and S43†), suggesting that DNA-mediated interaction with FCD-3 might be responsible for the observed internalization.

To evaluate the cytotoxicity of FCD-3, HeLa cells (cervical cancer) and human dermal fibroblasts (HDF) were continuously exposed to FCD-3 at concentrations from 10^−3^ to 2000 μg mL^−1^ for 1 h, 1 day, and 3 days (Fig. 4). Metabolic competence was assessed using Alamar Blue (AB), and the number of live cells was obtained with calcein. A concentration-dependent toxic effect was observed in both HeLa and HDF treated cells. For HeLa, the lethal concentration (LC_50_) was found to be 100 μg mL^−1^ after three days of exposure, while higher concentrations of FCD-3 were tolerated at shorter exposure times (LC_50_ at 1 h was 1500 μg mL^−1^). Reductive metabolism (RM) was halved after 1 h of exposure to 500 μg mL^−1^ FCD-3 and to 50 μg mL^−1^ after three days of exposure (Fig. S44†). In HDF cells (Fig. S45†), toxicity was detected at higher concentrations and/or longer exposure times to FCD-3 when compare to HeLa cells. LC_50_ was 10 times higher and RM_50_ was observed at 500 μg mL^−1^ FCD-3 but only after three days of exposure. The observed differences in cytotoxicity for each cell line could be attributed to the different rates of cellular uptake and nuclear accumulation of FCD-3.

**Fig. 4:**
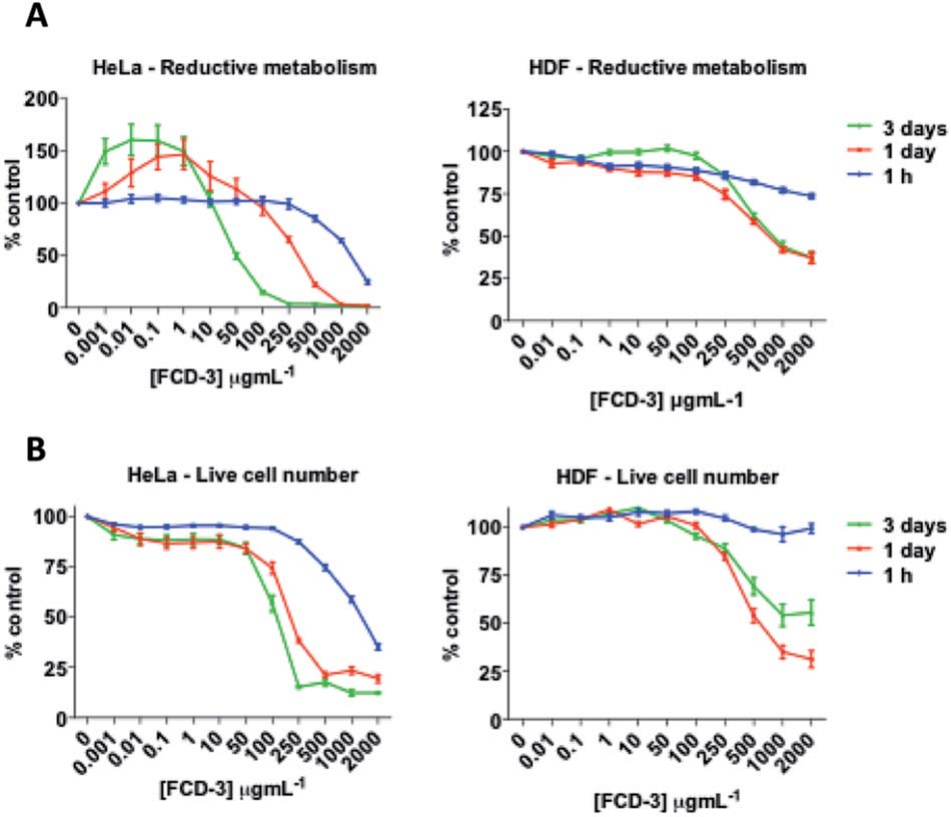
HeLa (left) and HDF (right) cells responses to incubation at 37 °C with FCD-3 (0–2000 μg mL^−1^) for 1 h, 1 day or 3 days. (A) Reductive metabolism (RM); (B) viable cell numbers (calcein). Data referenced to untreated controls (100%).

Next, we showed that photothermal activation of FCD-3 (500 μg mL^−1^) in deionized H_2_O and cell media DMEM (2 mL) was possible using blue-light-emitting diodes (LEDs), which were chosen due to their low cost, ease of use, and widespread availability (*λ*_em_ = 460 nm, Fig. S48†). Encouragingly, a 14 °C increase in temperature was measured after 90 min of illumination when compared to illumination of the solvent alone. Photothermal effects in FCD-3-treated cells post-illumination were then evaluated. An increase in temperature was also recorded in the wells of HeLa exposed to FCD-3 for 2 h at room temperature during the 90 min blue-LED irradiation, when compared to controls (FCD-3-treated cells kept in the dark). The temperature increase was proportional to FCD-3 concentration (Fig. S49†). LED-irradiation of HeLa cells treated with 50 μg mL^−1^ FCD-3 while keeping the cells at 37 °C resulted in temperatures reaching 56 °C after 90 min, while the same experiment in HDF cells only recorded 44 °C.^59^

Thermal increases in eukaryotic cells are known to have detrimental effects and lead to toxicity.^60^ To evaluate this, HeLa and HDF cells, cultured in 96-well plates, were exposed to FCD-3 at 1, 10, 50, 100 and 500 μg mL^−1^ for 2 h, before irradiation with blue LEDs for 30, 60 or 90 min. The FCD-3 containing medium was then removed and fresh medium was added. Cellular health was then assessed after 1 h, 1 day, and 3 days post-illumination using AB and calcein as before and compared to controls (cells that have not been exposed to FCD-3 but were LED-irradiated for the same amount of time). Excitingly, significant cell death and decrease in metabolism were observed for HeLa cells after incubation with at least 10 μg mL^−1^ FCD-3 followed by LED-irradiation (Fig. S50–S52†). In general, cell populations decreased by 75% and 80% after 1 day and 3 days post 30 min illumination when using concentrations as low as 50 μg mL^−1^ FCD-3. Increasing the blue-LED irradiation time to 60 and 90 min increased the toxic effect in HeLa cells. Conversely, in 50 μg mL^−1^ FCD-3-treated HDF cells after 30 min of illumination, RM was only diminished by 40% after 3 days (Fig. S53†) and only after exposures of up to 500 μg mL^−1^ FCD-3 a population reduction of 60% was observed after 1 day post-30 min LED irradiation. Interestingly, at 3 days HDF cells were recovered with only a 20% reduction relative to controls. This apparent recovery was observed for HDF at all FCD-3 exposure concentrations and after all LED-irradiation times (Fig. S54 and S55†). These differences in cellular toxicity after LED illumination in both cell lines could be attributed to differences in photothermal activation which in turn are likely associated to the differential cellular uptake of the nanoprobes.

Since FCD-3 contains a robust FCD-core (5) coated with *in situ* generated 2,5-deoxyfructosazine 4, and with the knowledge that 4 is a versatile molecule with anti-diabetic and anti-inflammatory properties,^50,61^ we wanted to determine whether FCD-3 or just 4 alone in solution was responsible for the cell killing effects observed. To this end, HeLa cells were incubated with FCD-3, 4 or core FCD-5 at two different concentrations (50 and 100 μg mL^−1^ with regard to the concentration of 4) for 2 h at 37 °C.^62^ Following the same process as before, the cells were then subjected to LED irradiation or kept in the dark for 60 min after which cell viability and metabolism were measured after 24 and 72 h and compared to controls (Fig. 5, S56 and S57†). Generally, no significant toxicity was observed in cells incubated with either 4 or FCD-5 either in the dark or under LED illumination, and only cells incubated with FCD-3 and subjected to LED-illumination showed an 80–90% reduction in cell viability as before.^63^

**Fig. 5:**
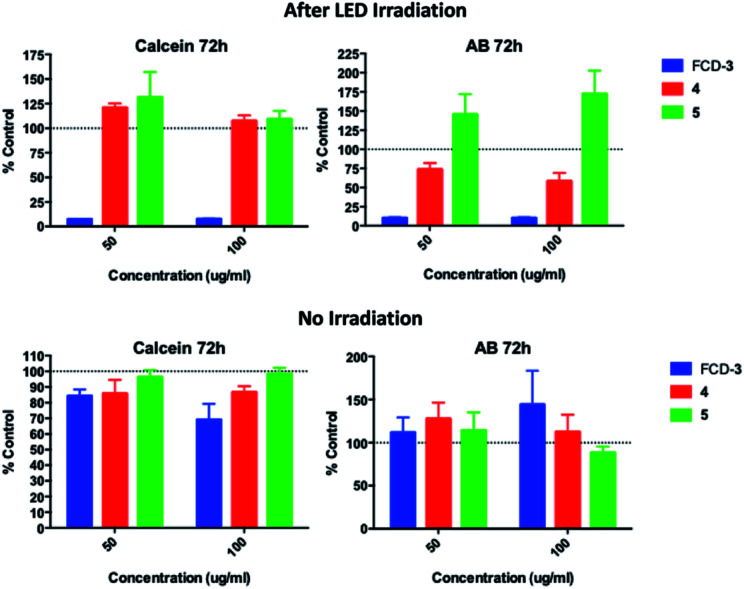
HeLa cell viability (calcein) and metabolism (AB) for cells incubated with FCD-3, 4 or 5 and post 60 min LED irradiation or in the dark. Data referenced to untreated controls subjected to the same illumination conditions (shown as 100%).

To better understand the mechanism of toxicity, dihydroethidium (DHE) was used next to assess the production of cytosolic reactive oxygen species (ROS) in FCD-3-treated cells 1 h post illumination. In both HeLa and HDF, ROS production levels were similar to those of dark controls, except with exposure to FCD-3 of 500 μg mL^−1^ and 90 min illumination which diminished DHE cytoplasm fluorescence by 50% in HeLa cells (Fig. 6). These results suggest that differences between HDF and HeLa responses to FCD-3 exposure/blue-LED illumination are not likely ROS associated.

**Fig. 6:**
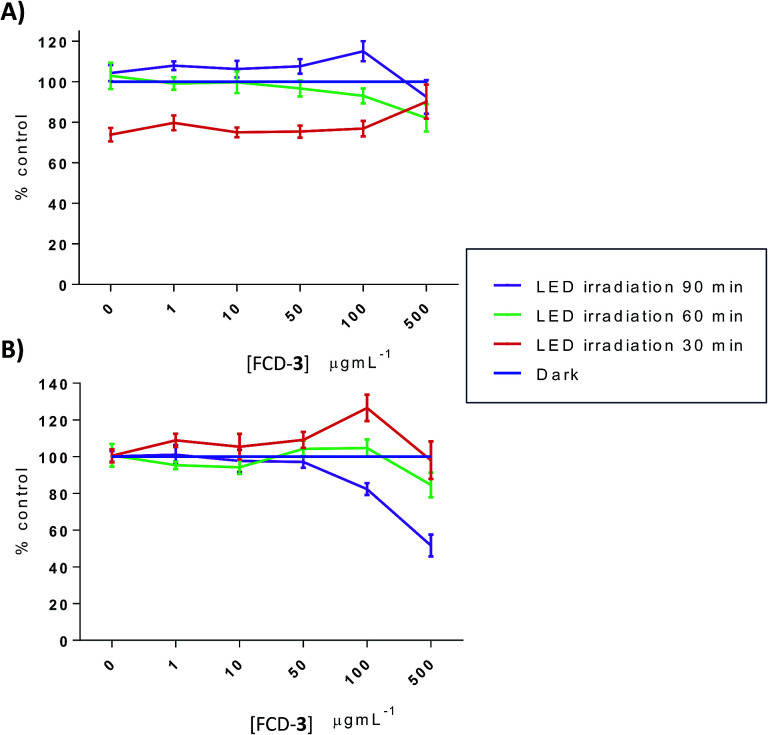
Percentage of cytosolic DHE fluorescence loss induced by reactive oxygen species (ROS) after exposure to FCD-3/blue-LED treatment relative to non-irradiated (dark) controls exposed to equal concentrations of 3 in (A) HeLa cells and (B) HDF cells.

Anaerobic glycolysis which produces adequate adenosine 5′-triphosphate (ATP) and biological components is the hall-mark of cancers to meet the rapid metabolic requirements.^64^ To investigate whether ATP levels had been affected upon treatment with FCD-3 and LED irradiation, intracellular ATP levels were determined in HeLa cells using a luciferin–luciferase luminescence ATP detection assay. HeLa cells were exposed to FCD-3 at 1, 10, 50, 100 and 500 μg mL^−1^ and incubated for 2 h before 90 min blue-LED irradiation. ATP determination was conducted at 1 h, 1 day and 3 days post-illumination. Controls kept in the dark did not experience a significant reduction in ATP levels after exposure to FCD-3. However, in cultures irradiated with blue-LED, ATP levels were reduced in a concentration-dependent manner with 1 day and 3 day levels being diminished by 65% and 75%, respectively, at 1 μg mL^−1^ FCD-3, relative to dark controls (Fig. 7).

**Fig. 7:**
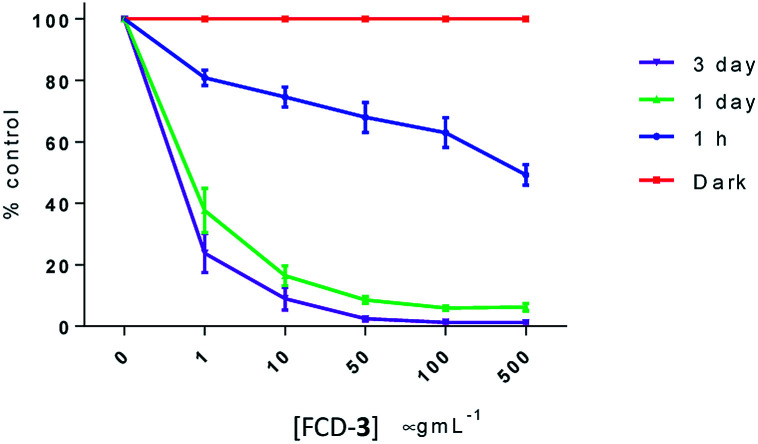
ATP depletion in HeLa after 2 h of exposure to FCD-3 followed by 90 min blue-LED illumination compared to non-irradiated (dark) controls at equal FCD-3 exposures. ATP luminescence was measured after cells were washed and after a period of 1 h, 1 day or 3 days under standard culture conditions.

To explore if metabolic products derived from FCD-3 exposure to cells could induce toxicity, naïve HeLa and HDF cells were incubated with the supernatant and lysates of FCD-3 exposed cultures at different concentrations (1–500 μg mL^−1^) for 2 h before 90 min blue-LED illumination. FCD-3 suspended in the cell culture medium in the absence of cells was irradiated as the control and RM and live cell numbers were quantified after 1 day of exposure. Cell lysates caused no toxic effects in either HeLa or HDF cells. Supernatants of irradiated HeLa cells were toxic only at the highest concentration of FCD-3 (500 μg mL^−1^), as determined by measuring cell viability and reductive metabolism (Fig. S58 and S59†), while no significant effects were measured in HDF cells at all concentrations tested (Fig. S60 and S61†). Moreover, NMR spectroscopy analysis of pre- and post-90 min illumination of FCD-3 in water revealed no visible changes in the structure of the FCDs (Fig. S62†). These results suggest that the cytotoxicity observed is not likely due to the generation of new metabolic or photochemical FCD by-products.

## Conclusions

In conclusion, we have developed a facile, three minute large-scale synthesis of a novel green-emissive carbon-based nanomaterial FCD-3 that can be used to target HeLa cancer cells preferentially over healthy epithelial HDF cells under mild activation conditions. The carbon-based probe contains a stable amorphous core and is decorated with 2,5-deoxyfructosazine 4 as the major component, which together give rise to the nanomaterial's unique properties. We demonstrate that FCD-3 is internalized into both HeLa and HDF cells partly through passive mechanisms and trafficked into the nucleus by interactions with DNA, albeit at significantly distinct rates depending on the cell line. This differential uptake can be exploited in the LED-activated selective killing of HeLa over HDF cells after 60 min of LED-illumination using as little as 50 μg mL^−1^ FCD-3. The killing is believed to be caused by an increase of intracellular localized temperature followed by ATP depletion. Interestingly, neither the FCD core 5 or surface component 4 are individually able to induce cancer cell death as observed for FCD-3. Furthermore, we show that metabolic products from FCD-3 treated cells do not elicit toxic effects on naïve cells. These novel metal-free bifunctional nanoprobes have the potential, with further development, to be effective theranostic agents in cancer therapy.

## Conflicts of interest

There are no conflicts to declare.

## Supplementary Material

NA-001-C9NA00293F-s001
