# Supplementary material for: Selective photothermal killing of cancer cells using LED-activated nucleus targeting fluorescent carbon dots
Source: Nanoscale Adv. 2019 Jul 15;1(8):2840–6. doi: 10.1039/c9na00293f (PMC9417209; doi:10.1039/c9na00293f)
Supplement: NA-001-C9NA00293F-s001 [file NA-001-C9NA00293F-s001.pdf]

Electronic Supplementary Material (ESI) for Nanoscale Advances.
This journal is © The Royal Society of Chemistry 2019

Selective Photothermal Killing of Cancer Cells Using LED-Activated Nucleus Targeting Fluorescent Carbon Dots

Stephen A. Hill,[1§] Sadiyah Sheikh,[1§] Qiaoyu Zhang,[1] Lorena Sueiro Ballesteros,[2] Andrew Herman,[2] Sean A. Davies,[1] David J. Morgan,[3] Monica Berry,[1] David Benito-Alifonso, M. Carmen Galan[1]*

[1]School of Chemistry, University of Bristol, Cantock's Close, Bristol, UK. [2] School of Cellular and Molecular Medicine and Faculty of Life Sciences Flow Cytometry Facility, University Walk, Bristol, UK. [3]Cardiff Catalysis Institute, School of Chemistry, Cardiff University, Park Place, Cardiff

Contents: **Page number:**

**General experimental**	**S2**
**Synthesis of green amine-coated carbon dots (FCD-3)**	**S2**
**Physico-chemical characterisation of FCD-3**	**S3**
**Cell cultures, toxicity assays and confocal microscopy protocols**	**S21**
**Cellular uptake and organelle colocalization measurements for FCD-3 and confocal microscopy images.**	**S24**
**Cell toxicity assays**	**S44**
**References**	**S60**

General Experimental

Chemicals were purchased and used without further purification. FCD-**3** preparation was conducted in a domestic microwave (Wilko's Homebrand) 800W. Concentration centrifugation tubes were GE Healthcare Life Sciences VIVASPIN 6/20 with a 10,000 Da molecular-weight cut off filter. Extracts were concentrated under reduced pressure using both a Büchi rotary evaporator at a pressure of either 15 mmHg (diaphragm pump) and 0.1 mmHg (oil pump), as appropriate, and a high vacuum line at room temperature. ^{1}H & ^{13}C (HSQC and HMBC) NMR spectra were measured in D_2O on 500 MHz Varian. ^{1}H & ^{13}C NMR chemical shifts are quoted in parts per million (ppm) and referenced to the residual solvent peak (D_2O: ^{1}H = 4.79 ppm); and coupling constants (J) given in Hertz. Assignments were made, where necessary, with the aid of HSQC and HMBC NMR experiments. Fourier-transformed infra-red (FTIR) spectroscopy was conducted on a Bruker ATR. Dynamic Light Scattering (DLS) and Zeta analysis (ZP) are carried out using Malvern Instruments, Nano-S90 Red Laser Model ZEN1690 for DLS and Nano-Z ZEN 2600 for Zeta potential. Zeta potential measurements were conducted in distilled H_2O. The morphology and structure of G-FCD were examined by transmission electron microscopy (TEM) on a Jeol 2100F with an accelerating voltage of 200 kV. A drop of the FCD-**3** MeOH/H_2O (1:2) suspension (5 mg/mL) was carefully applied to a 200-mesh carbon-coated copper grid and dried at ambient temperature for TEM characterization. Fluorescence measurements were made and conducted on a Perkin-Elmer LS45, 230V in either 700 or 3500 µL quartz cuvettes (ThorLabs) using a Pulsed Xenon lamp. Absorbance measurements were conducted on Cary UV-Vis 50 spectrophotometer in either 700 or 3500 µL quartz cuvettes (ThorLabs). Quantum yield of fluorescence measurements were conducted between both Perkin-Elmer LS45 and Cary UV-Vis 50 spectrophotometer in in either 700 or 3500 µL quartz cuvettes (ThorLabs), relative to fluorescein (in 0.1M NaOH (aq)). X-ray photoelectron spectroscopy (XPS) was performed using a Kratos Axis Ultra DLD spectrometer, using monochromatic Al ka radiation operating at 144 W power (12 mA x 12 kV). For analysis, samples (dissolved in MeOH/H_2O (1:2)) were pipetted on to clean gold wafers and the solvent evaporated under vacuum in the fast-entry lock of the spectrometer. Analysis of all regions was taken at a pass energy of 20 eV with a 0.1 eV step for high resolution scans, and 160 eV and a 1 eV step for survey spectra. All data was subsequent calibrated to the lowest C(1s) line for saturated C-C bonds taken to be 285 eV and quantified using sensitivity factors supplied by the manufacturer in CasaXPS v2.3.17PR1.1. Thermogravimetric analysis (TGA) was performed on a TA Instruments Q100 calorimeter at a scan rate of 10 °C/min under nitrogen.

Synthesis of Green Amine Coated Carbon Dots (FCD-3)

Glucosamine hydrochloride (1.00 g, 4.63 mmol) was dissolved in deionised H_2O (20 mL) and agitated to achieve complete dissolution in a 250 mL Erlenmeyer conical flask. 1,3-diaminobenzene (0.55 g, 5.10 mmol) dissolved by sonication in methanol (10 mL) was then added to the glucosamine solution and further agitated to ensure solution homogeneity. The conical flask was then placed in a domestic microwave, in a well-ventilated area, and the solution heated for 3 min (800 W, 80% power). A viscous brown oil-like material was afforded, which was dissolved in water (10 mL) and centrifuged (8500 rpm, 30 mins) through a VIVASPIN 20 (10,000 MWCO

filter). The bulk solution was then reduced either *in vacuo* or lyophilised to yield FCD-**3** as either a brown oil-like material or a brown powder respectively, 1-1.5 g.

Physico-Chemical Characterisation of FCD-3

A)

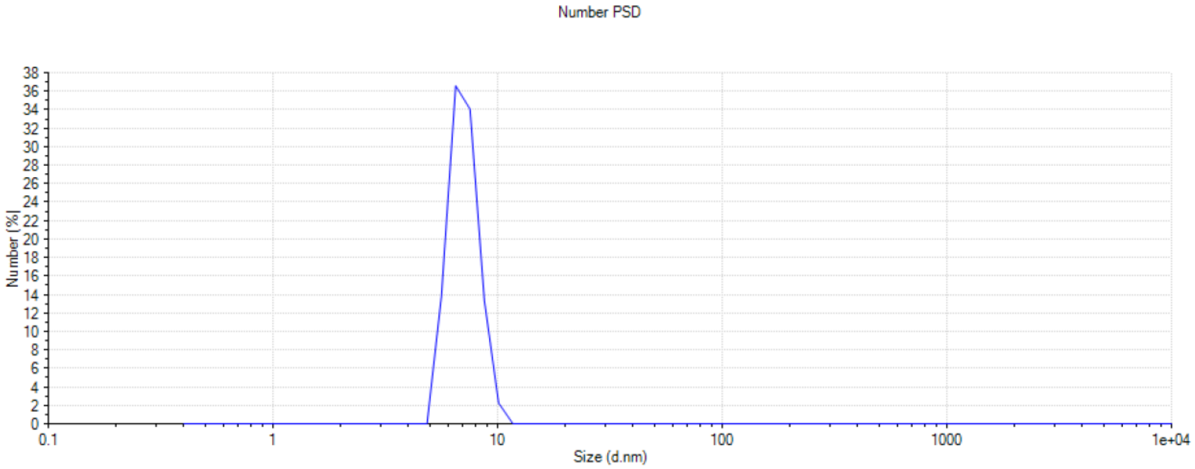

B)

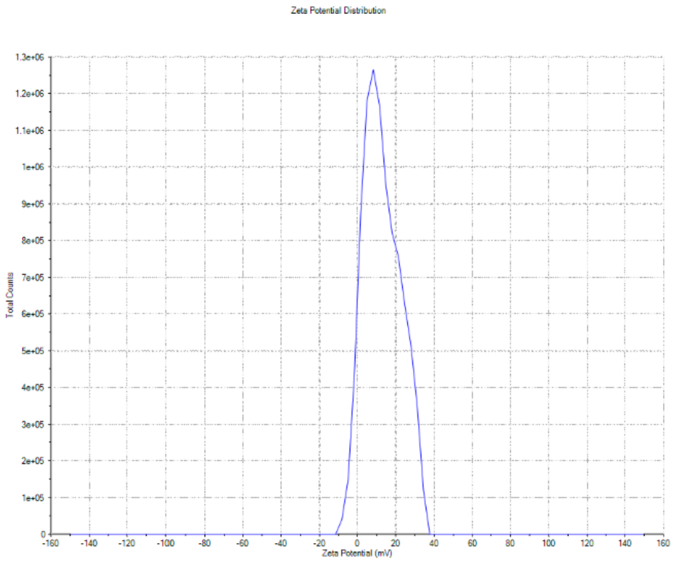

Figure S1. A) DLS profile of FCD-**3** exhibiting a hydrodynamic diameter ranging between 6-9. B) Zeta-potential measurement for FCD-**3** (12.05 mV).nm.

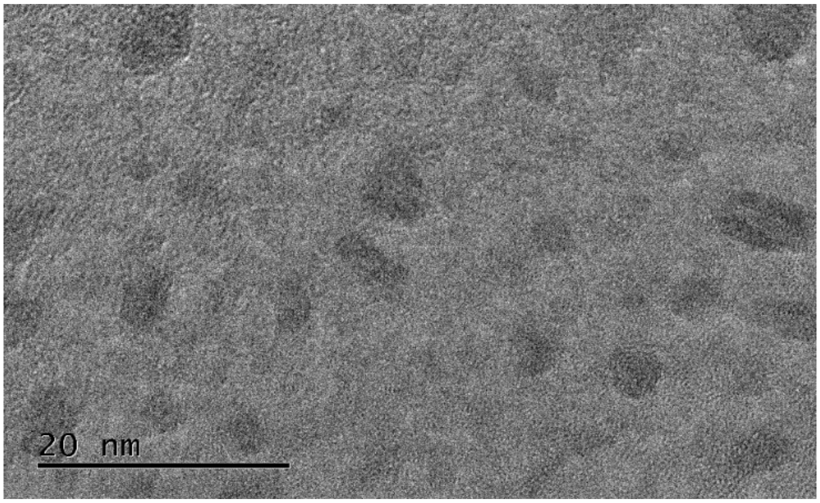

Figure S2. HR-TEM image of FCD-**3** showing an amorphous core as no lattice planes are visible.

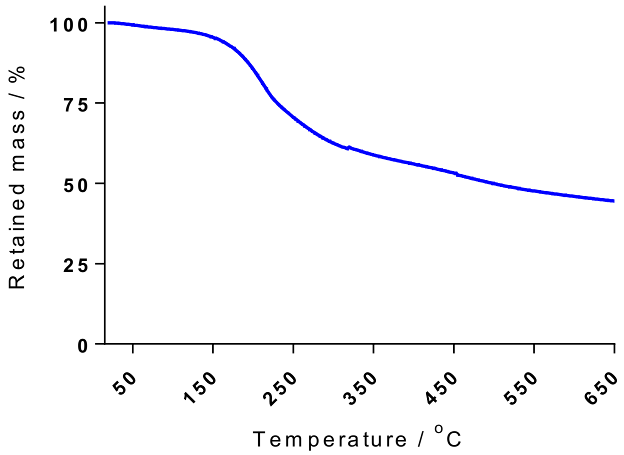

Figure S3. Thermogravimetric analysis curve of FCD-**3** showing % mass change as a function of increasing temperature.

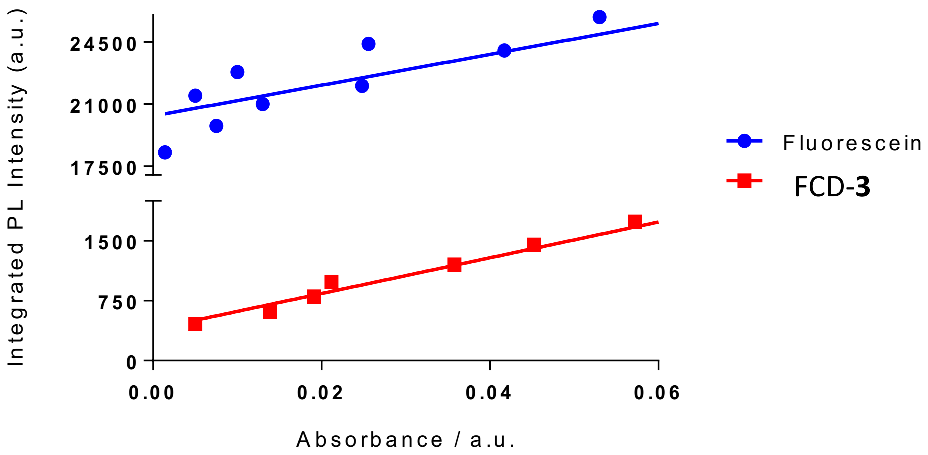

Figure S4. FCD-**3** relative quantum yield of fluorescence determination

The quantum yield of fluorescence (QY or Φ) for FCD-**3**, in aqueous media (refractive index; η= 1.33) was calculated by measuring the integrated fluorescence intensity (excitation 340 nm) at a range of absorbance values below 0.10. The resulting curve was plotted and compared against fluorescein (free acid) in 0.1M NaOH (aq) (refractive index; η= 1.33), which is a standard of known QY of 95%. (I = slope η = refractive index).

$$\Phi_{FCD} = \Phi_{Fluorescein}\, x \left(\frac{I_{FCD}}{I_{Fluorescein}}\right) x \left(\frac{{\eta_{FCD}}^2}{{\eta_{Fluorescein}}^2}\right)$$

$$\Phi_{FCD} = 0.95\, x \left(\frac{30329}{86851}\right) x \left(\frac{1.33^2}{1.33^2}\right) = 0.33$$

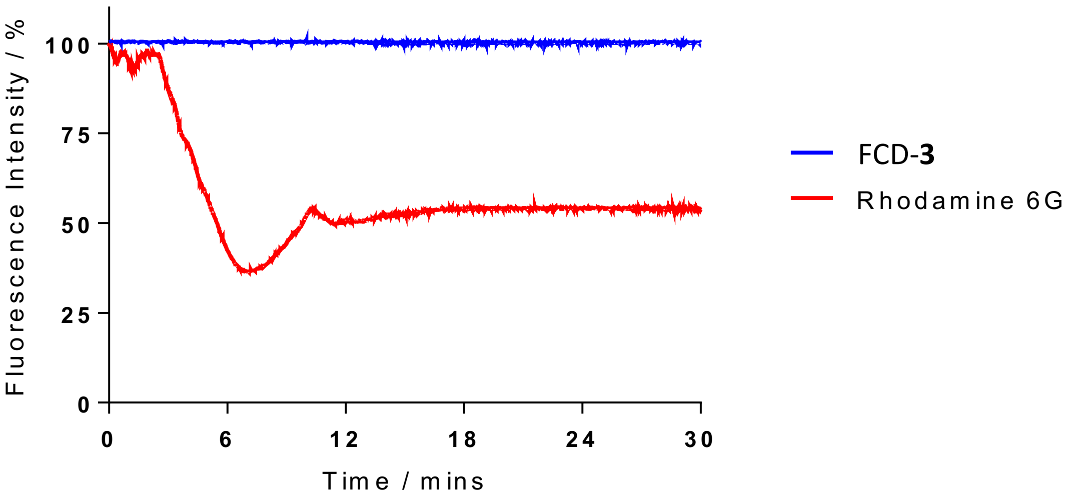

Figure S5. Fluorescence emission intensity of FCD-**3** (λ_{ex}= 460 nm, λ_{em}= 525 nm) and Rhodamine 6G (λ_{ex}= 360 nm, λ_{em}= 600 nm) monitored with continuous irradiation

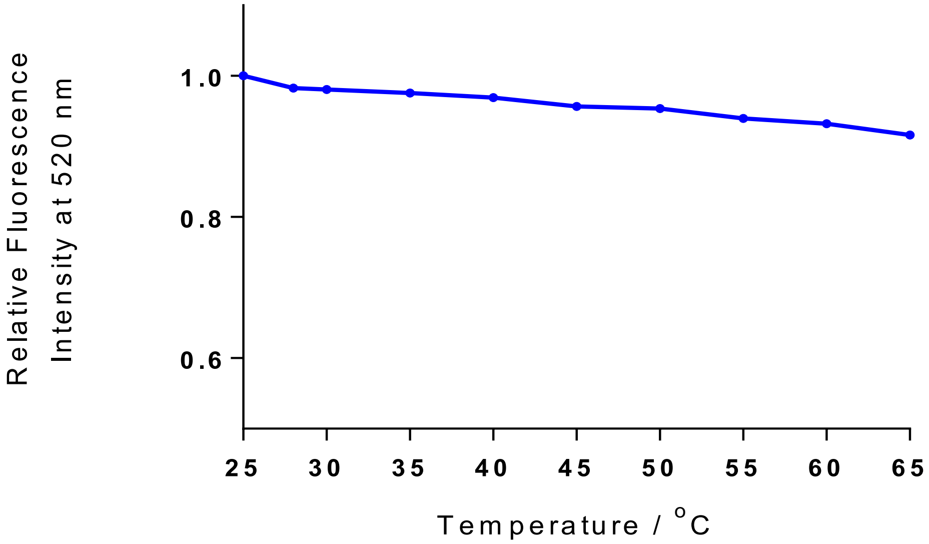

Figure S6. Fluorescence emission intensity of FCD-**3** (λ_{ex}= 460 nm, λ_{em}= 525 nm) as a function of temperature

Fluorescence Emission Quencher Screening Protocol

To 3 mL of distilled H_2O in a 3.5 mL quartz cuvette was added 50 µL of FCD-**3** solution (1 mg mL^{-1} in distilled water) and then the solution was thoroughly mixed. The quencher concentration was then achieved via addition of 20 µL of quencher solution (appropriate concentration) and the resultant solution was thoroughly mixed, before standing for 5 minutes. The fluorescence (always using the same fluorimeter settings) of the solution was recorded before and after quencher addition

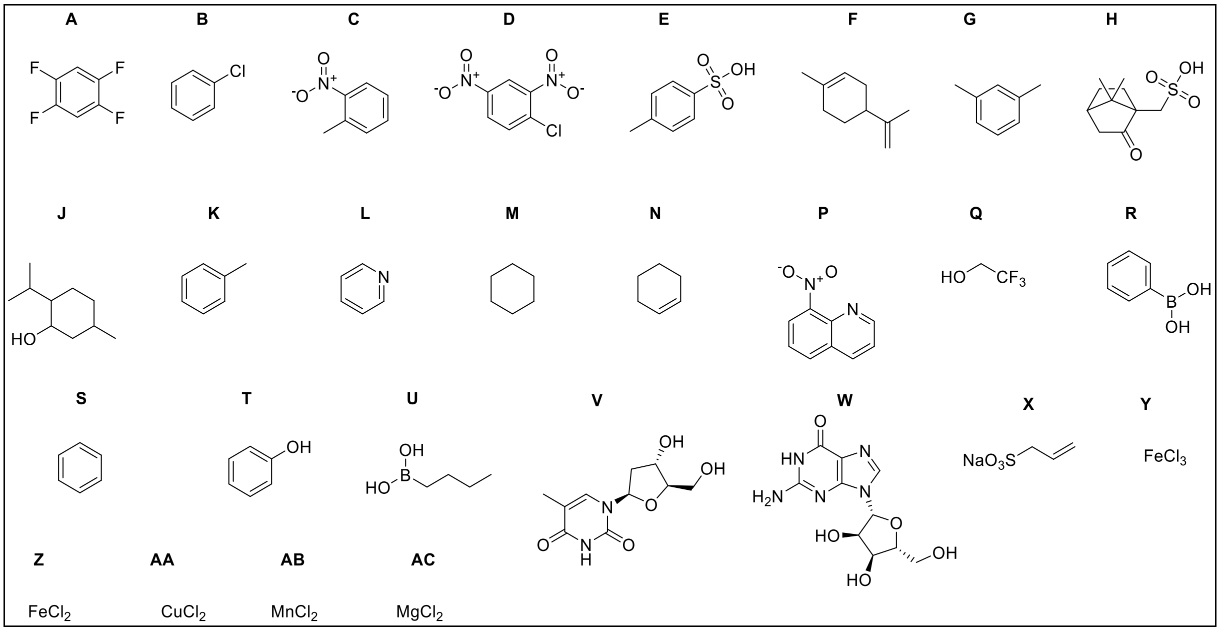

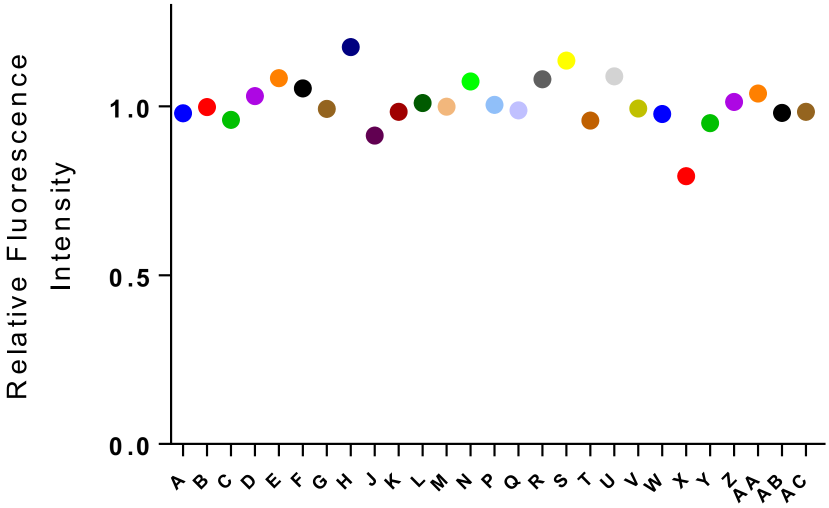

Figure S7. Fluorescence emission intensity for FCD-**3** (λ_{ex}= 460 nm, λ_{em}= 525 nm) upon addition of a series of aromatics, natural products and metal ions (A-AC) at a concentration of 500 µM. The data confirms the stability of the material under a range of potential fluorescent quenchers.

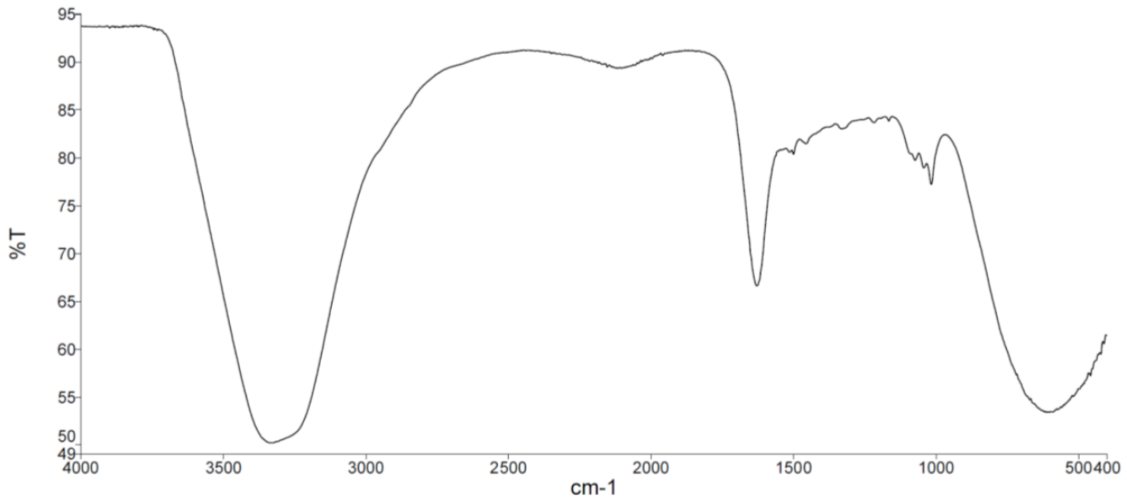

Figure S8. FTIR spectrum of FCD-**3**. Key features: 3338 cm^{-1} (O-H/N-H), 1629 cm^{-1} (amide C=O), 1016 cm^{-1} (C-O/C-N), 612 cm^{-1} (C-Cl)

Table S1. Elemental composition for **FCD-3**

	**Elemental Composition / %**				
**Batch**	**C**	**H**	**N**	**Cl**	**O**
**1**	37.85	7.99	13.29	12.51	28.35
**2**	41.65	7.30	13.01	12.51	29.75
**3**	40.05	6.92	12.45	13.44	25.54
**4**	37.42	7.06	12.90	12.50	27.48
**5**	40.49	6.83	13.09	11.61	27.98
**6**	42.28	6.92	12.66	12.52	25.62
**Average**	**39.96**	**7.17**	**12.90**	**12.52**	**27.45**

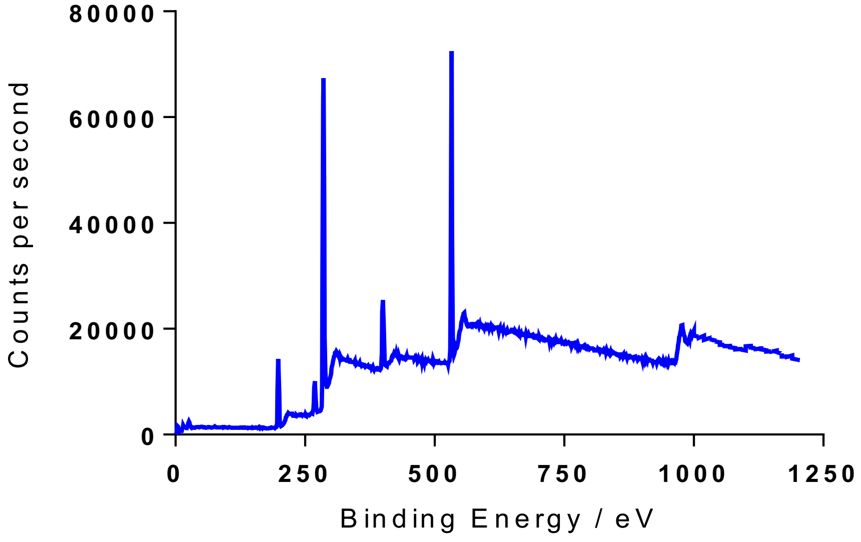

Figure S9. Wide scan XPS spectrum of FCD-**3**. Key features: 285 eV (C 1s), 532 eV (O 1s), 400 eV (N 1s) and 197 eV (Cl 2p)

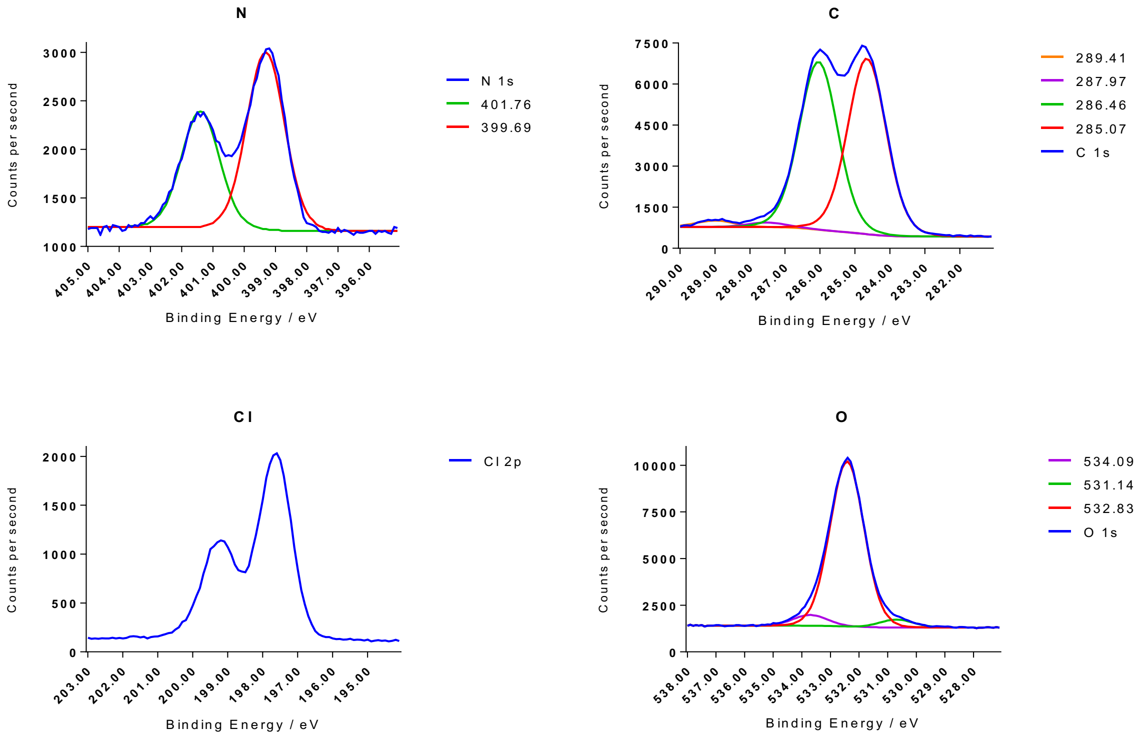

Figure S10. High-resolution XPS for O, N, C, and Cl, and peak fits (blue lines represent raw data) for FCD-**3**.

Table S2. XPS functional group assignments for FCD-**3** based on fittings shown in Figure S10.

Element	Binding Energy / eV	Deconvolution Functionality
**Cl**	198.1	$-NH_3Cl$ (ammonium chloride)
**C**	285.0	C-C (alkyl/aromatic)
**C**	286.5	C-C (C-OH/C-O-C/C-N)
**C**	287.9	C=O
**C**	289.4	O-C=O
**N**	399.7	NH_x
**N**	401.1	Imide/N-C=O/NH_4^+
**O**	531.1	OH (aromatic)
**O**	532.8	C-O (OH/C-O-C) or O-C=O/N
**O**	534.1	C=O (aromatic)

A)

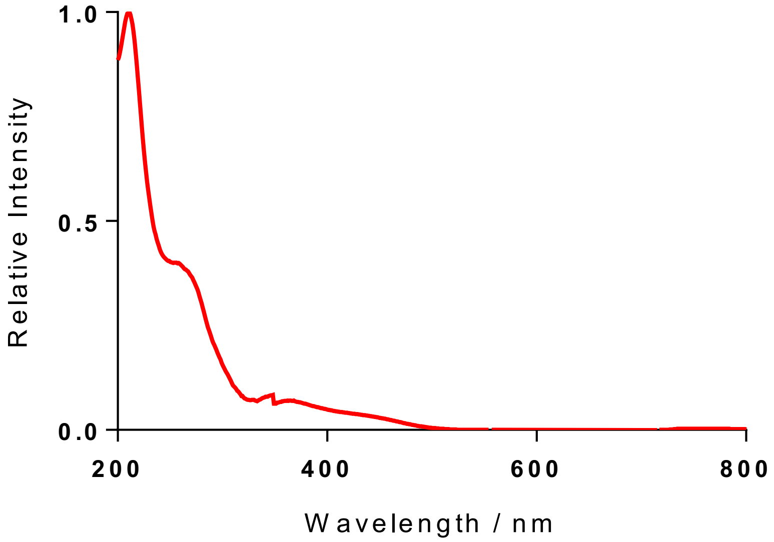

B)

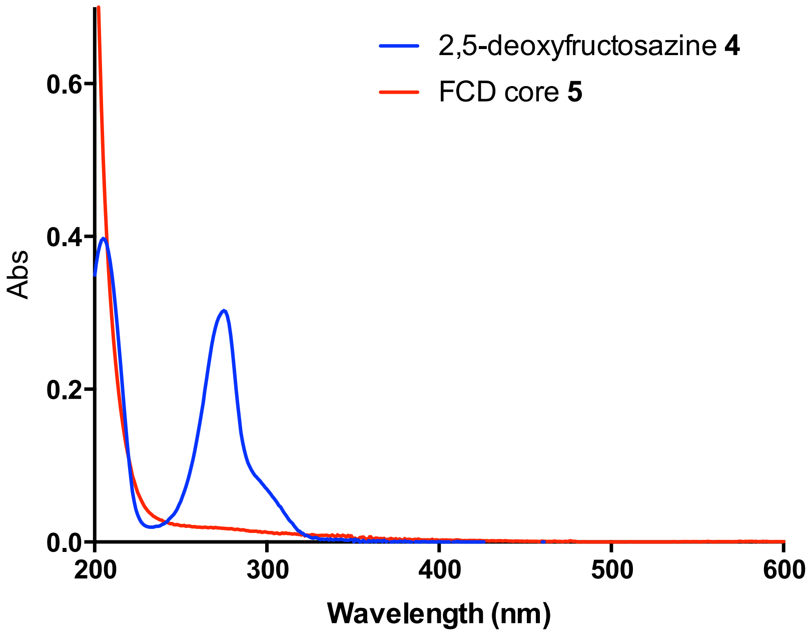

Figure S11. UV-vis spectrum of A) FCD-**3**. Key features: 212 nm, 260 nm (aromatic π-π* transitions), and 370 nm (n-π* aromatic C=C or C=O/C=N); B) 2,5-deoxyfructosazine **4** and FCD-core **5**.

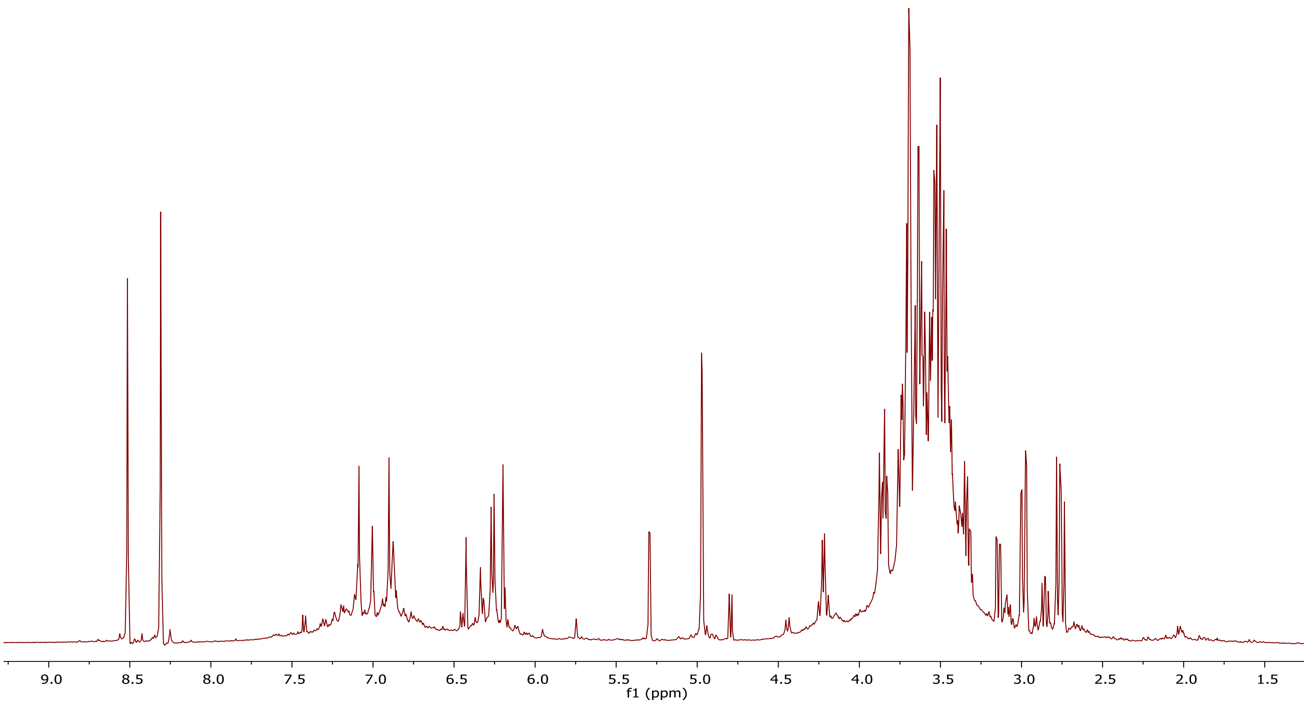

Figure S12. ^{1}H Presat NMR of FCD-**3**. Key features: pyrazine signals (8.51, 8.30, 5.01 ppm), complex aromatic architectures (7.50-6.00 ppm), polyhydroxylated architectures (4.50-2.50 ppm).

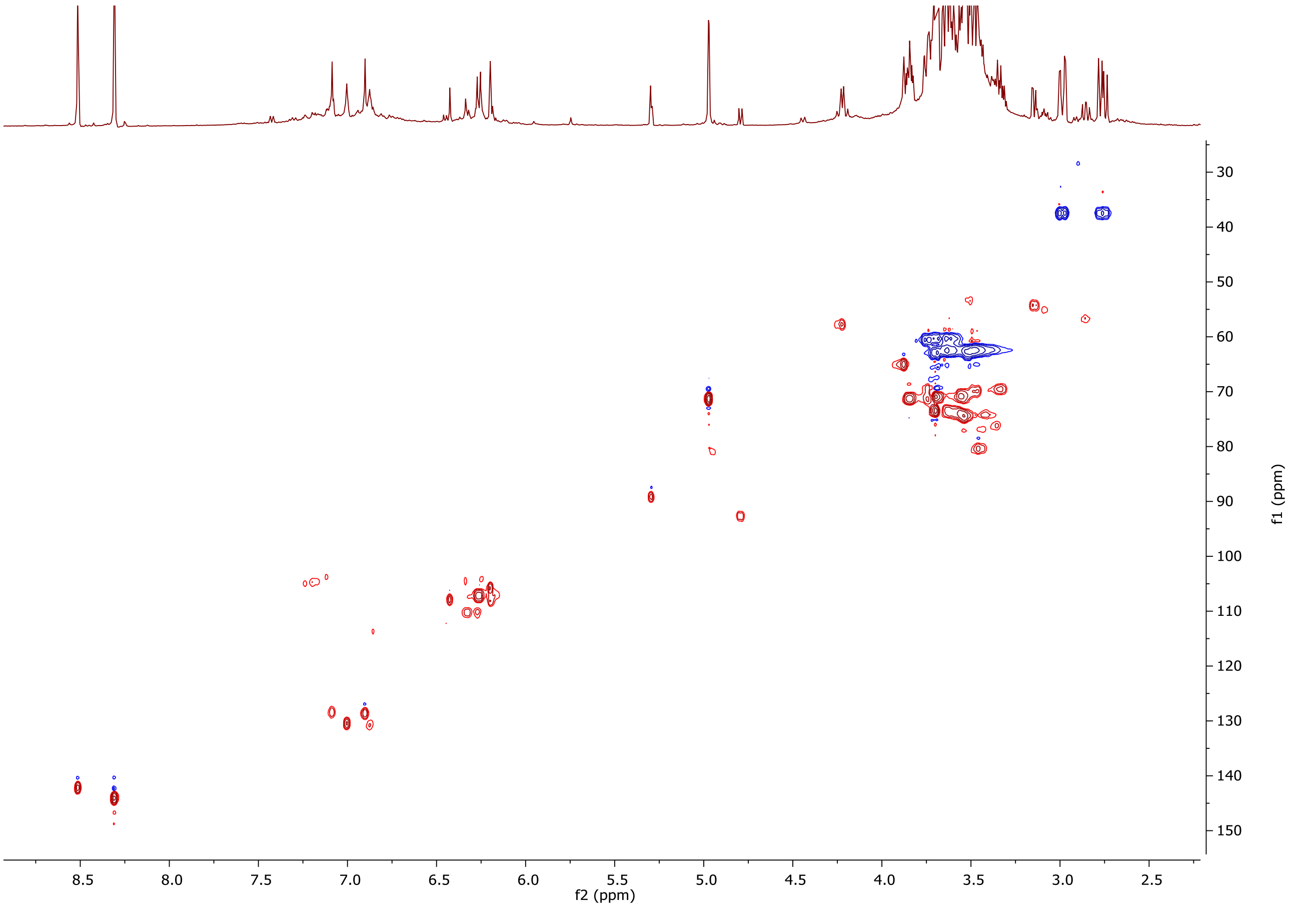

Figure S13. ^{1}H- ^{13}C HSQC NMR of FCD-**3** (D_2O, 500MHz)

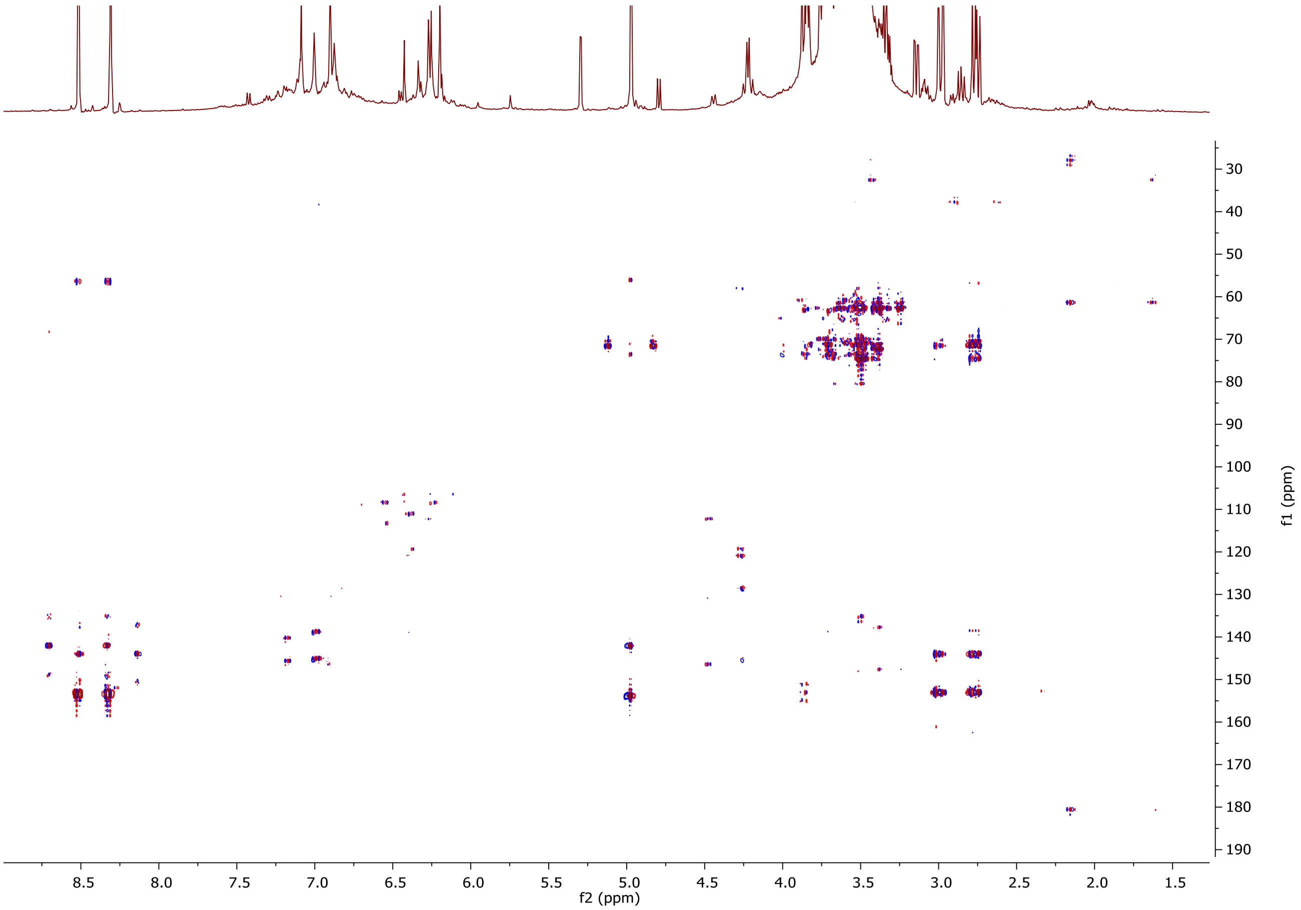

Figure S14. ^{1}H- ^{13}C HMBC NMR of FCD-**3** (D_2O, 500MHz)

The ^{1}H-^{13}C HSQC spectrum shows that signals at 8.30 and 8.51 ppm are coupled to N-heteroaromatics connected to ^{13}C signals at 144 and 141 ppm with a 1-bond correlation, respectively (Figure S13). The singlet centred at 5.01 ppm is correlated to a signal at 70 ppm, hence not having anomeric character. ^{1}H-^{13}C HMBC spectrum highlighted that the signal at 8.30 ppm had long-range connectivity with ^{13}C signals at 142 and 153 ppm, and 8.51 ppm with 144 and 153 ppm (Figure S14). The signal 5.01 ppm had cross-peaks 142 and 153 ppm. Signals in the 2.50-4.00 ppm region had 1-bond correlations with ^{13}C signals between 60-70 ppm and were indicative of C-O bonds i.e. carbohydrate or polyhydroxylated architecture (Figure S12). These signals are suggestive of both incomplete pyranose dehydration and the formation of fructosazine-type motifs.[1-3]

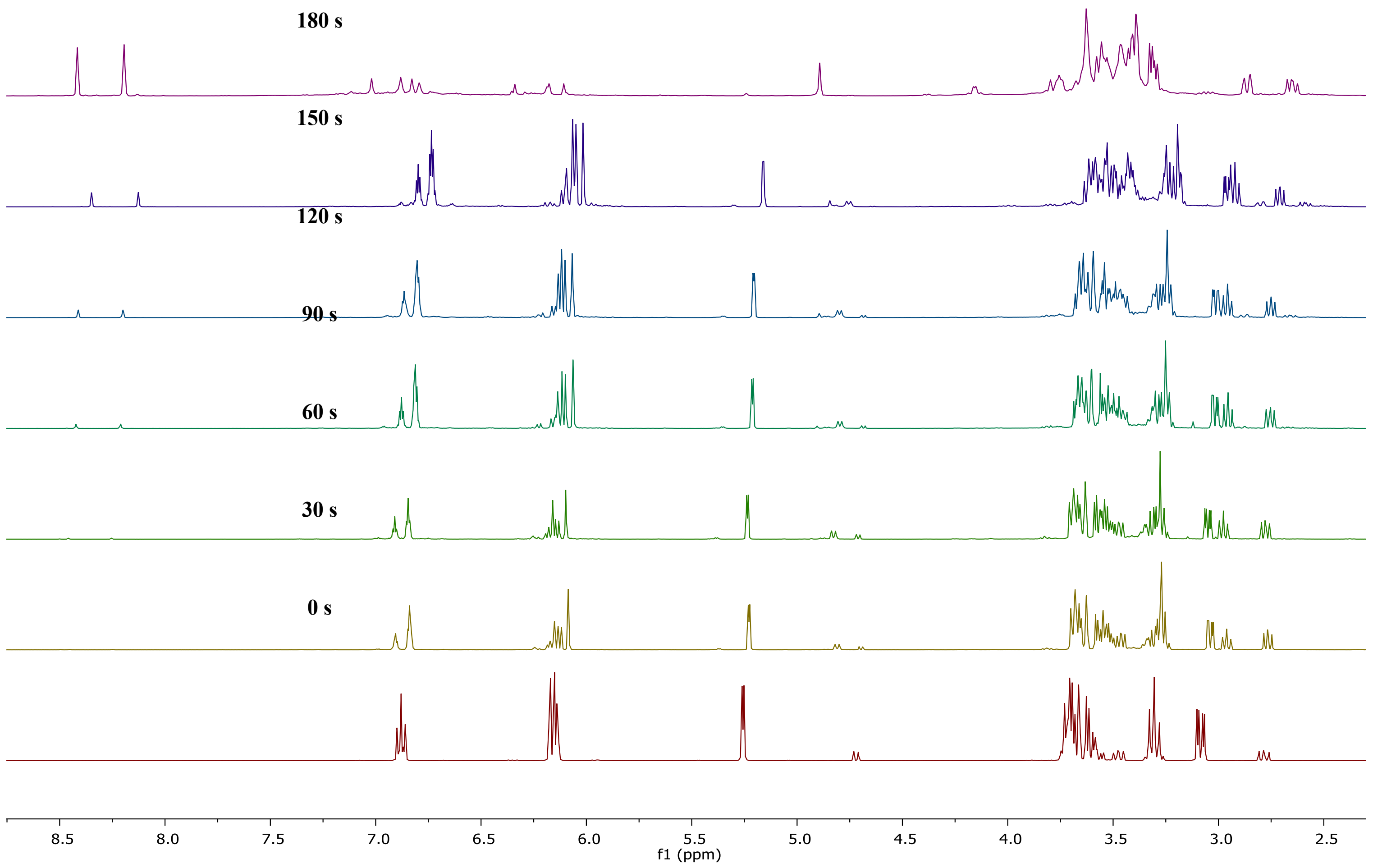

Figure S15. ^{1}H NMR reaction monitoring of FCD-**3** at time points up to 180 s (D_2O, 500 MHz)

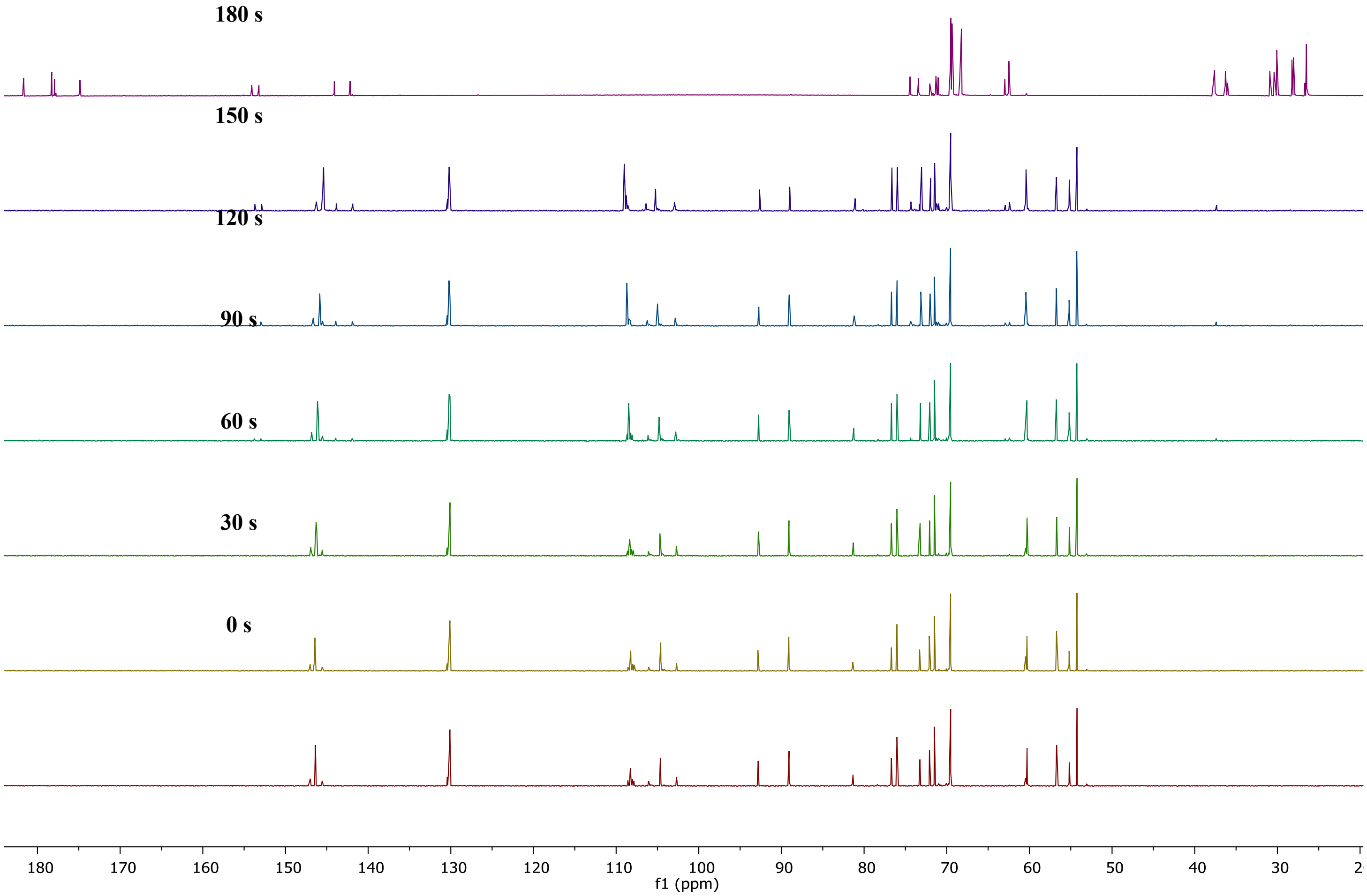

Figure S16. ^{13}C NMR reaction monitoring of FCD-**3** at time points up to 180 s (D_2O, 125 MHz)

Isolation and structural characterization of surface bound 2,5-deoxyfructosazine 4

FCD-**3** was subjected to G15-Sephadex size exclusion column chromatography using methanol as the eluent using gravity (35 cm height and 1.7 diameter). Three fractions were obtained which contained core FCD-**5,** FCD-**3** and 2,5-deoxyfructosazine **4**.

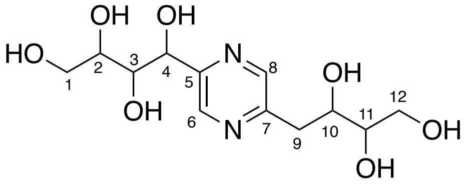

2,5-deoxyfructosazine 4

^{1}H NMR (500 MHz, Deuterium Oxide) δ 8.58 (d, J = 1.4 Hz, 1H, H5), 8.40 (d, J = 1.3 Hz, 1H, H8), 5.02 (d, J = 2.2 Hz, 1H, H4), 3.90 (ddd, J = 9.7, 6.3, 3.4 Hz, 1H, H10), 3.78 – 3.65 (m, 4H, H2, H11, H1a, H12a), 3.62 – 3.47 (m, 3H, H1b, H12b, H3), 3.09 (dd, J = 14.3, 3.3 Hz, 1H, H9a), 2.85 (dd, J = 14.3, 9.8 Hz, 1H, H9b). **^{13}C NMR (126 MHz, D_2O)** δ 153.94 (C5), 153.18 (C7), 144.06 (C8), 142.14 (C6), 74.38 (C3), 73.38 (C11), 71.31 (C4), 71.26 (C10), 71.01 (C2), 62.88 (C1), 62.43 (C12), 37.51 (C9).

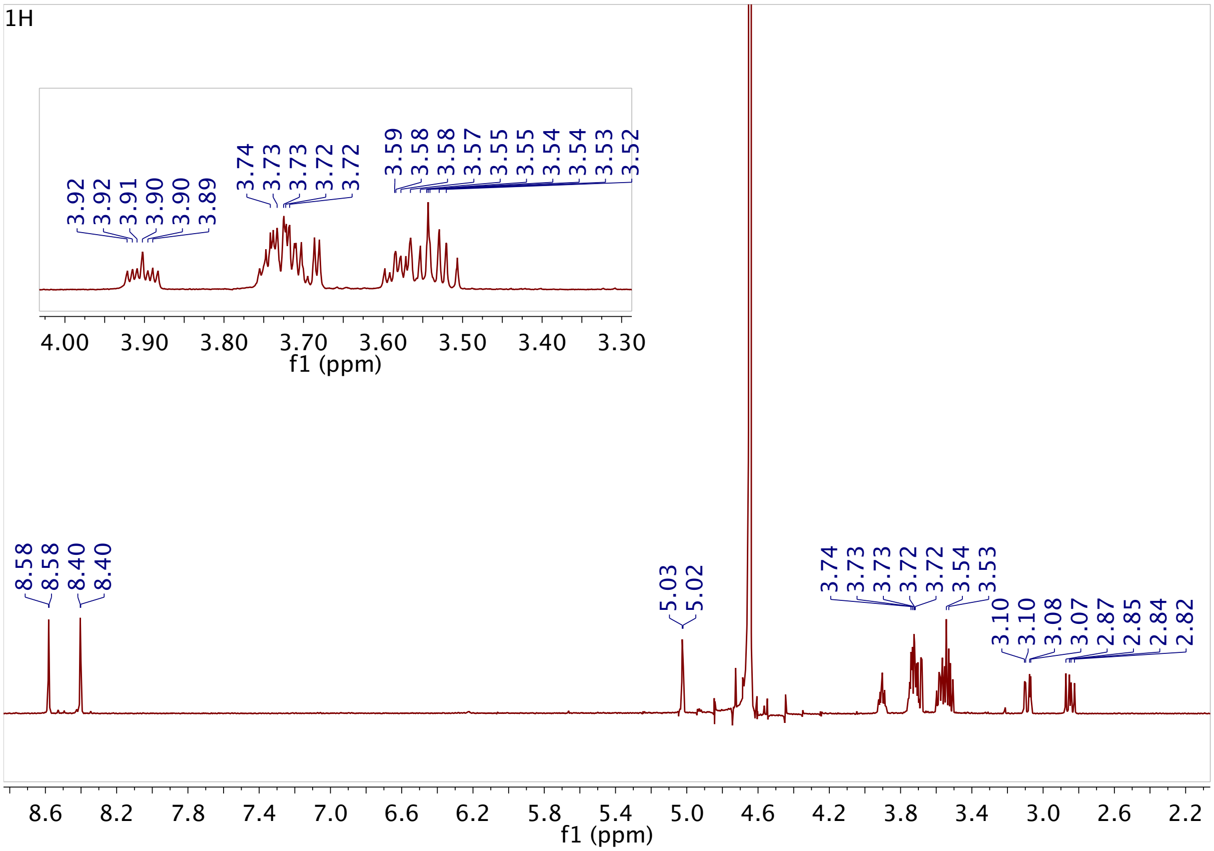

Figure 17. ^{1}H-NMR spectra of **4**.

A) HSQC spectra

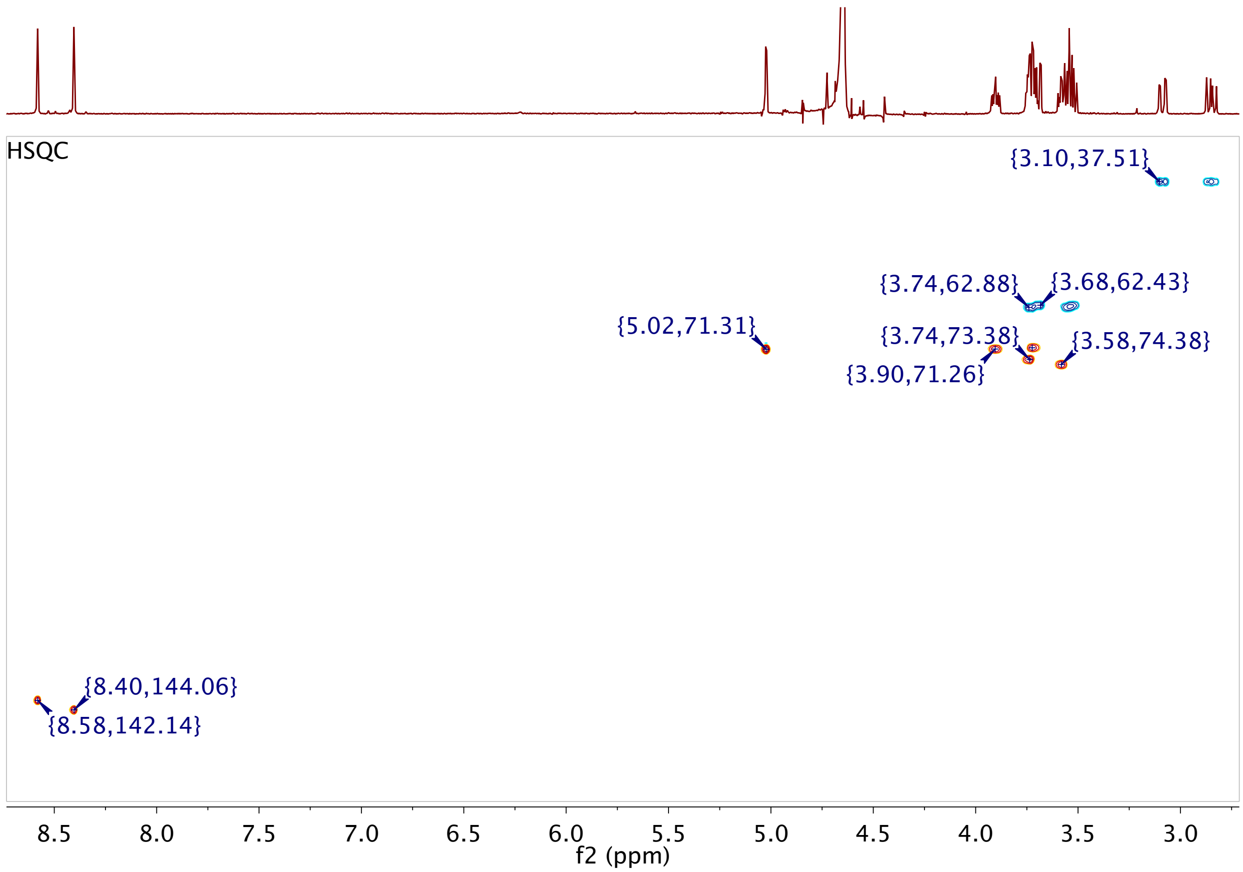

B) HMBC spectra

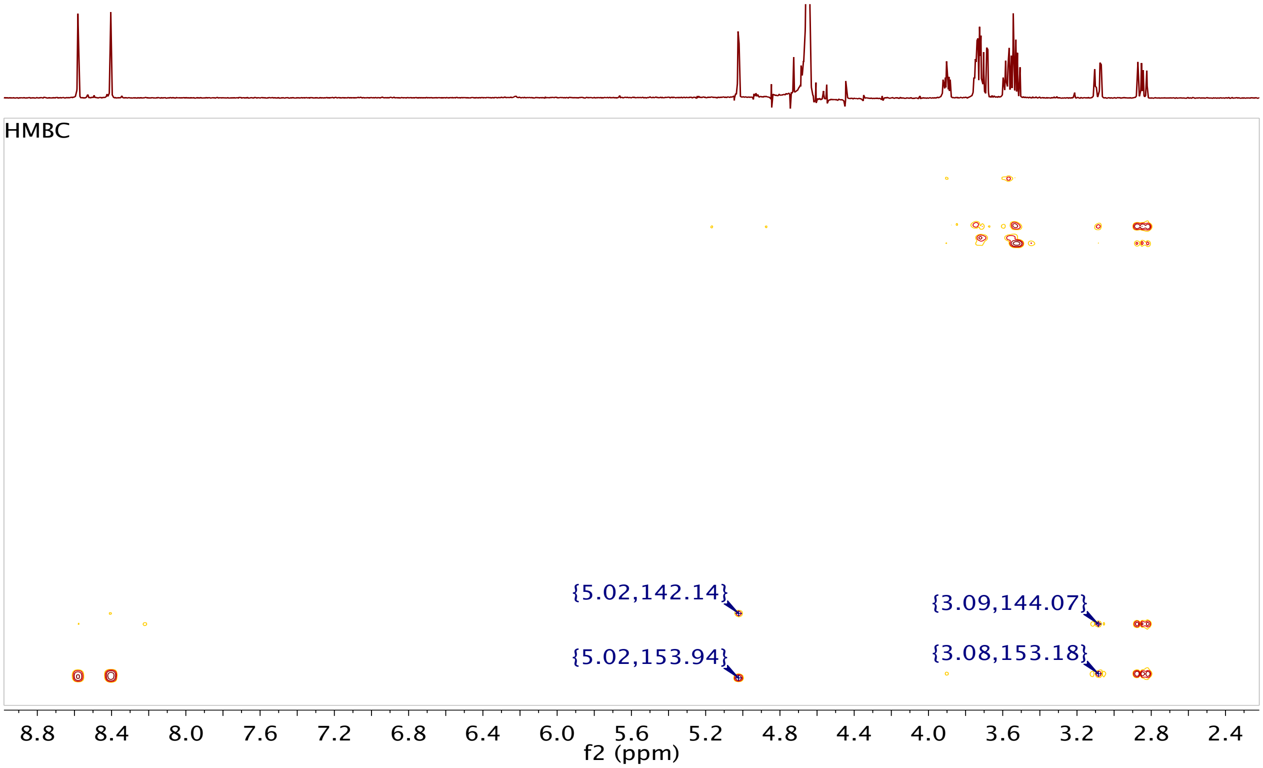

Figure 18. A) HSQC spectra and B) HMBC spectra of **4**.

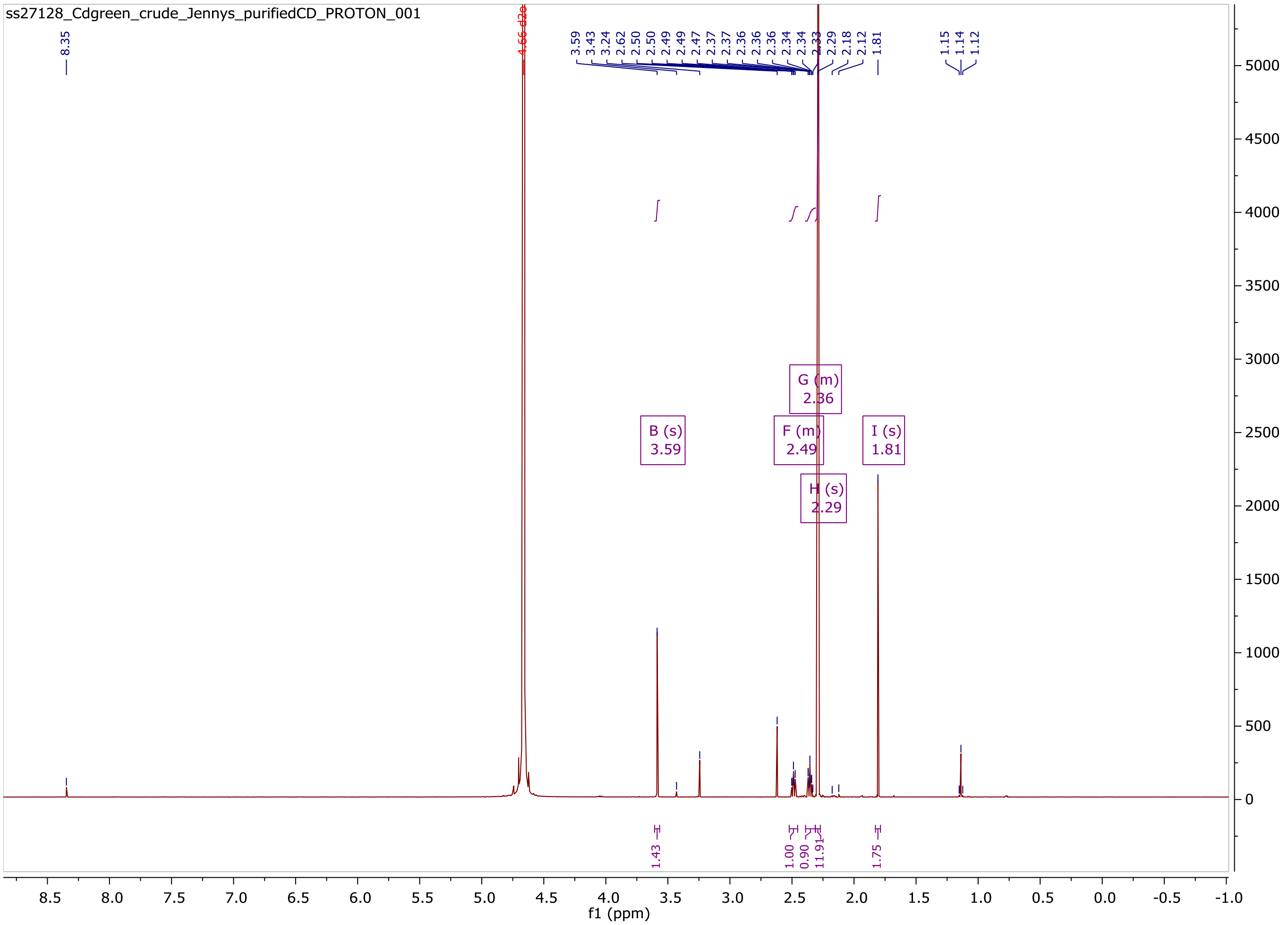

Figure 19. ^{1}H-NMR spectra of core FCD-**5**

Diffusion-Ordered NMR Spectroscopy (DOSY) Experiments

To further confirm that 2,5-deoxyfructosazine is surface bound to FCD-**3**, Diffusion-Ordered NMR Spectroscopy (DOSY), which probes the diffusion coefficient for each of the components of the ^{1}H-NMR spectrum, was acquired in D_2O for FCD-**3**. Diffusion ordered spectroscopy (DOSY) of **3** showed that it had a single diffusion coefficient of 3.83 x10^{-6} cm^2 s^{-1}confirming that **4** is bound to the FCD-core in **3**.

FCD-3

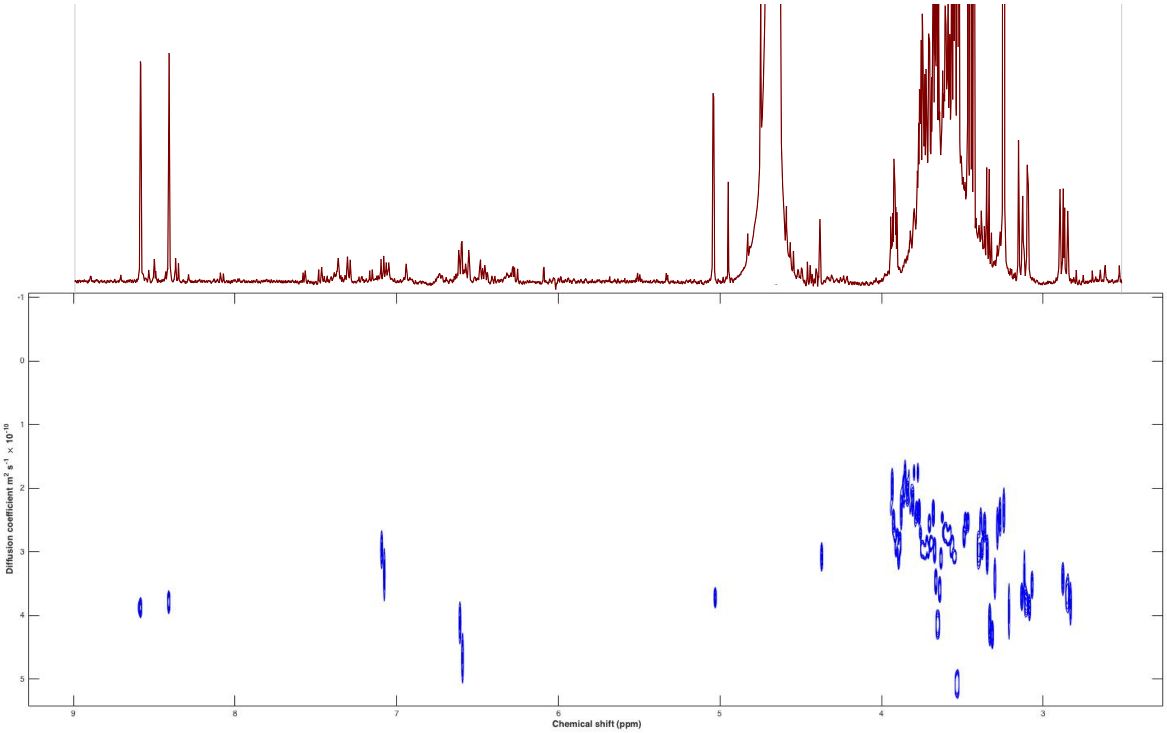

Figure S20. DOSY spectra for A) 2,5-deoxyfructosazine **4** and B) FCD-**3**

Cell culture, confocal microscopy, toxicity assay and related protocols

Cell Culture

HeLa cells (ECACC, Public Health England) were maintained in Dulbecco's Minimal Essential Medium (DMEM) with glucose 1g/L. Human dermal fibroblast cells (HDF, neonatal, ECACC) were grown in Minimal Essential Medium MEM. Media were supplemented with antibiotic-antimycotic (AntiAnti), Glutamax and 10% foetal bovine serum (FBS). Confluent cultures were dissociated using trypsin (Tryp LE Express) and plated at 10^4 cells/well in either petri dishes (Mat-Tek 35 mm, with 14 mm glass microwell) for confocal imaging, or 96-well plates for toxicity tests (8 replicates per condition, i.e. toxicant concentration and time point) for 1 h, 1 day and 3 days). For flow cytometry (FACS) then 24-well plates were used, maintaining the seeding density as above.

For toxicity assays, FCD-**3** at final concentrations of 2000, 1000, 500, 250, 100, 50, 10 $\mu g \cdot mL^{-1}$ followed by 10 times dilutions down to 10^{-3} $\mu g \cdot mL^{-1}$ were added 24 h after plating, in medium with reduced FBS (5%) to avoid protection from high serum. FCD-**3** were added at a 1 in 100 dilution from distilled H_2O. All cell culture media and additives were purchased from Invitrogen, Life Technologies (Thermo-Fisher).

Confocal Microscopy

Incubations were carried out using 50 $\mu g \cdot mL^{-1}$ of FCD-**3** at 37°C unless stated otherwise. Intracellular markers were purchased from ThermoFisher Scientific: CellLight® Reagents *BacMam 2.0 for early (Rab5a) and late endosomes (Rab7a); ER-Tracker™ for the endoplasmic reticulum; BODIPY TR ceramide for the Golgi; and LysoTracker™ for lysosomes, carbocyanine-based MitoTracker® (not dependent on oxidation status) for mitochondria, and NucRed™ Live 647 ReadyProbes™ for nuclei. The manufacturer's specifications were followed for tracker visualization. All images were acquired on a Leica SP5 confocal system equipped with a Leica DMI 6000 inverted microscope. For the excitation of the FCD-**3** 405 nm, 450 nm or 488 nm excitation could be used: *all images shown are using 405 nm, with emission measured 500 nm ± 20*. Cells were imaged in Living Cell Imaging Solution (ThermoFisher Scientific). The images were analysed using Volocity software (PerkinElmer). Global Pearson's Correlation or Coefficient (GPC) was measured for different organelle trackers and FCD-**3**. GPC is a measure used to quantify the colocalisation of two fluorophores (with different emission profiles) having been imaged in two separate fluorescence channels.[4] Areas of positive correlation are considered and a colocalisation between 0 and 1 is generated, following inter-channel analysis. An average GPC value was calculated for each organelle.

Toxicity assays

Cell metabolism was assessed using 5% v/v AlamarBlue in medium without any FBS. AlamarBlue is a cytosolic substrate for reductive enzymes (resazurin to resorufin) whose fluorescence spectrum changes on reduction. The total number of live cells was evaluated using Calcein AM, which is transformed into fluorescent Calcein, in the cytoplasm of live cells. After exposure to FCD-**3**, cultures were washed with PBS, and incubated with both dyes simultaneously for 1h before fluorescence emission (Calcein: λ_{ex} 485 nm, λ_{em} 515 nm; AlamarBlue: λ_{ex} 540 nm, λ_{em} 590 nm)

was measured at on a plate reader. Dihydroethidium (DHE) is a fluorescent substrate used to establish the level of reactive oxygen species (ROS). Oxidative reaction of DHE with ROS leads to formation of ethidium. Monitoring fluorescence decrease (λ_{ex} 370 nm, λ_{em} 420 nm) allows determination of cytosolic ROS production.

DNA/RNA FRET Flow Cytometry

FCD-**3** were incubated at 50 µg·mL^{-1} (in 500 µL of media, in 24-well plate) for 2 h at 37 ^{o}C. The appropriate FRET acceptor-dye (DNA intercalators Enzo NuclearRedID or DRAQ-5 and RNA intercalator F22 RNA Selective Dye were purchased from Thermofisher Scientific and used as instructed by the manufacturer) was added at 90 min and incubated for 30 min. After the combined 2 h, the cells were washed with phosphate-buffered saline before dissociation with Acutase (HeLa cells) or Trypsin (HDF cells). After centrifugation cells were resuspended in Live Cell Imaging Medium. Data were acquired using a Novocyte 3000 flow cytometer (ACEA Biosciences, San Diego, CA, USA), and analysed using FlowJo Software v10.4 (FlowJo, LLC Ashland Oregon, USA). A 405nm solid laser was used to excite the G-FCD and the emission was measured in the VL-2 detector (445/45nm band pass (BP) filter). A 488nm solid laser was used to excite F22 and Enzo dyes, the emission was measured in the BL-2 (585/40nm BP) and BL-4 (675/30nm BP) detector respectively. Finally, a 640nm solid laser was used to excite DRAQ5 and the emission was measured in the RL-1 detector (675/30nm BP). A minimum of 20,000 cells were analysed for each cell-dye pair. To that end, 20,000 events were recorded based on a light scatter gate that encompassed intact cells.

Blue LED Illumination effects

Illumination Assay

Blue-LEDs were purchased from LightingEVER, #4100057-BLUE, Voltage: 12V, Wattage, 24W, and mounted on a black cardboard base, over which clear-bottom 96-well plates were elevated 3 mm from the LEDs and placed under a box. Cells were exposed to FCD-**3** for 2 h at 37 ^{o}C at concentrations of 500, 100, 50, 10 1 and 0 µg mL^{-1}. The 96-well plates were then illuminated for 30, 60 or 90 min at room temperature. Controls were incubated in the same conditions but in the dark. All experiments were conducted in 8-replicates. After illumination medium was replaced with a fresh 100 uL aliquot of media. AlamarBlue and CalceinAM assays were performed after a further 1 h, 1 day, and 3 days, or after 1h for DHE. For time-course temperature assays, the temperature of the media was then measured at 5 or 10 minute time intervals using a Thermocouple (type K from Fisher scientific). The box and plate lid were removed at each time interval in order to measure the temperature of the media. For end-point measurements the box and plate lid were only removed at the end of the run to measure the temperature of the media.

Effects of cell-metabolised FCD-3

Cells were seeded on two 96-well and one 24-well plate, as previously described. Cells were exposed to FCD-**3**, on the 24-well plate, for 2 h followed by 90 min Blue-LED illumination at concentrations of 500, 100, 50, 10, 1 and 0 µgmL^{-1} 8-replicates per concentration. 1 h post-illumination the supernatants from the 24-well plate were transferred to the appropriate well on the 96-well plate. The exposed cells, were dissociated using Trypsin (Tryp LE Express) and lysed

through 3 freeze/thaw cycles in tissue culture medium. The lysate was then transferred to the appropriate well on a separate 96-well plate. As controls, media with FCD-**3** at the concentrations above were illuminated for 90 min, before being added to naïve cultures in a 96-well plate. Alamar Blue and CalceinAM assays were conducted 24 h post-transfer.

ATP Determination Assay

A luciferin/luciferase luminescence assay (ATP Determination Kit, A22066, Thermofisher Scientific) was conducted to quantify the energy state of cells. Cells were seeded on a 96-well plate, as previously described and exposed to FCD-**3** for 2 h at 37 ^{o}C, followed by 90 min Blue-LED illumination at concentrations of 500, 100, 50, 10, 1 and 0 µg mL^{-1}, with 8-replicates per concentration. The illuminated and non-illuminated cells were then incubated for 1 h, 1 day, and 3 days before quantitation of ATP was undertaken using the manufacturer's guidelines. Equivalent plates, acting as dark controls, were exposed to G-FCD but kept in the dark.

Cellular uptake and organelle colocalization measurements for FCD-3 and Confocal microscopy images.

- **HeLa Cells**

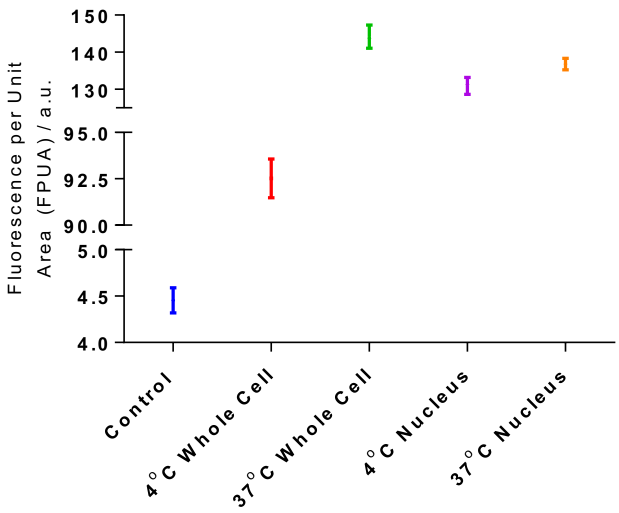

Figure S21. Fluorescence per unit area (FUA) of whole HeLa cells and their nuclei after exposure to 50 µgmL^{-1} of FCD-**3** for 2 h at 4°C and 37°C. Note that whole cell fluorescence at 37 °C is higher than at 4 °C, but the nuclear fluorescence is similar at 4°C and 37°C.

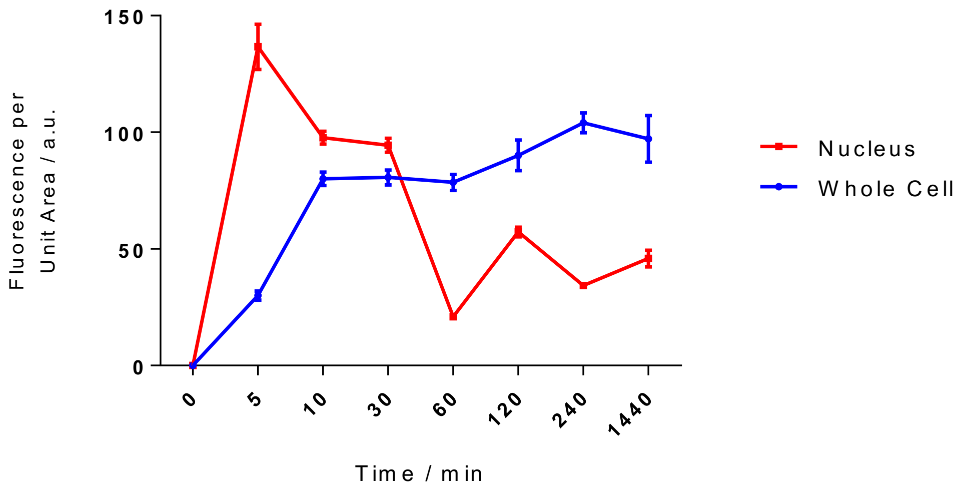

Figure S22. Fluorescence per unit area inside whole HeLa cells (blue) or nucleus (red) at different exposure times (5 min - 1 day) of 50 µgmL^{-1} of FCD-**3** at 37 °C . Note that the dynamics of nucleus and overall cell fluorescence are different: FCDs are rapidly internalized in the nucleus whereas uptake to the whole cell experiences a gradual increase of **3**.

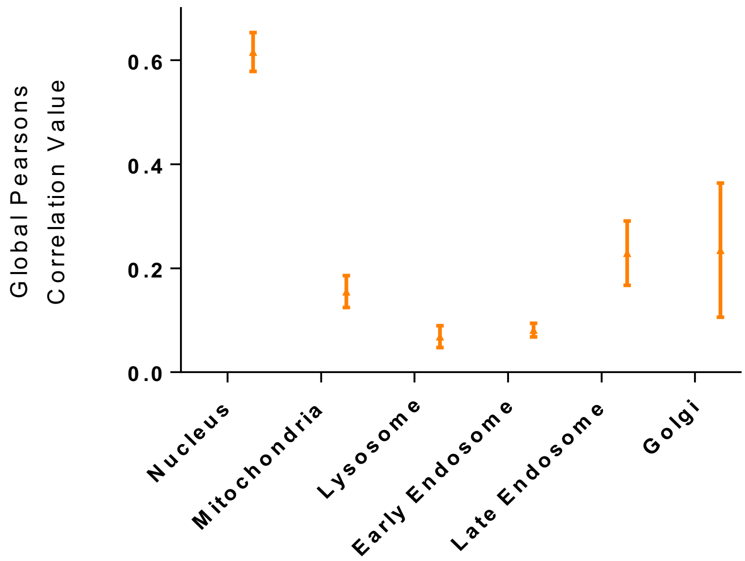

Figure S23 (Figure 2 in the main manuscript). Summary of colocalisation values (Global Pearsons Correlation) for FCD-**3** treated HeLa cells (50 μg·mL^{-1} after 2h at 37°C) with different organelle-selective fluorescent trackers.

The data indicates that FCD-**3** accumulates mostly in the nucleus and cytosol of HeLa cells with rapid internalization in the nucleus.

- **Human Dermal Fibroblasts (HDF) Cells**

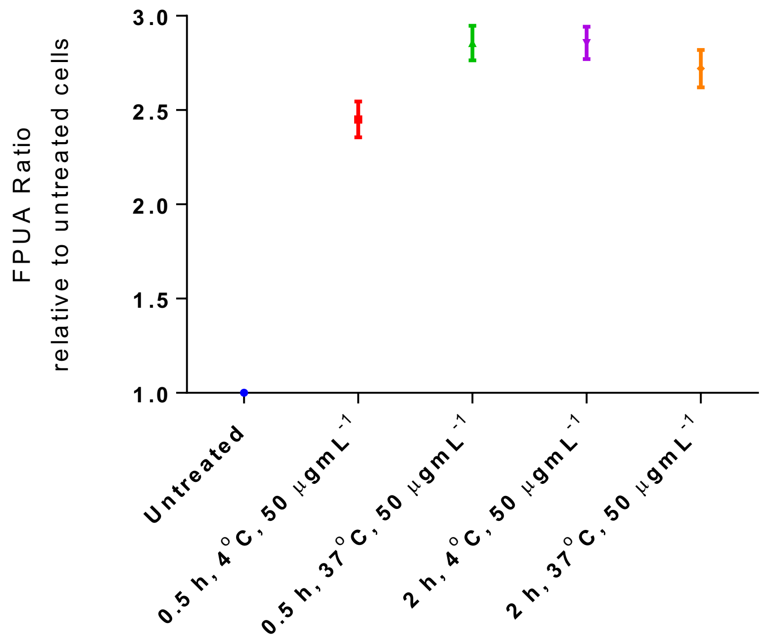

Figure S24. Fluorescence per unit area of whole HDF cells after exposure to 50 μgmL^{-1} of FCD-**3** for 2 h at 4°C and 37°C

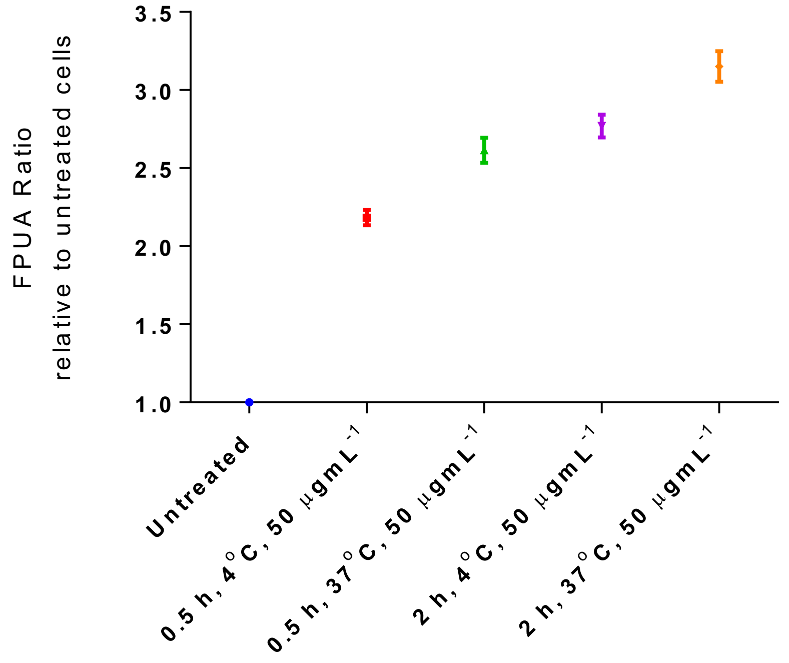

Figure S25. Fluorescence per unit area inside HDF nucleus after exposure to 50 µgmL^{-1} of FCD-**3** for 2 h at 4°C and 37°C

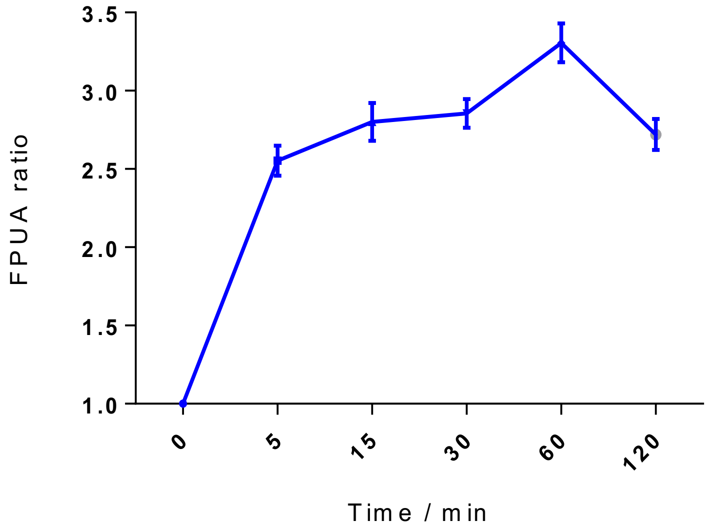

Figure S26. Fluorescence per unit area (FPUA) inside whole HDF cells (blue) at different exposure times (0 – 120 min) of 50 µgmL^{-1} of FCD-**3** at 37 °C relative to untreated cells.

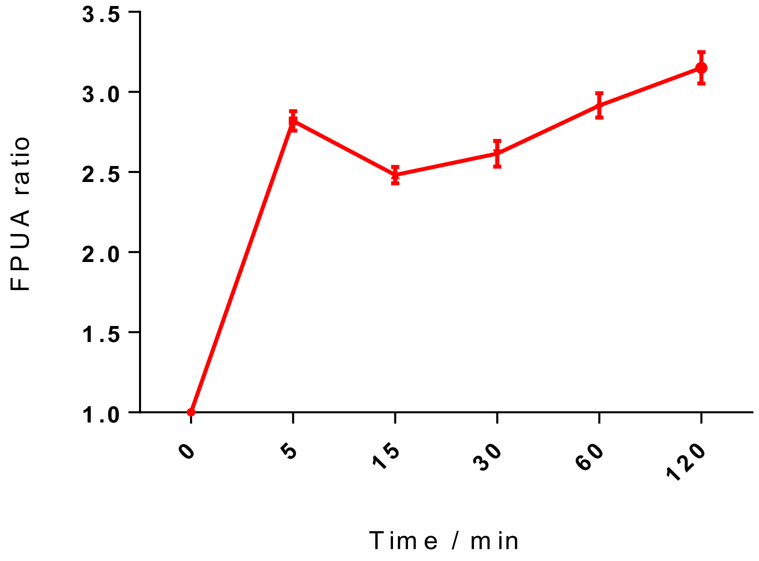

Figure S27. Fluorescence per unit area (FPUA) inside whole HDF cells (blue) at different exposure times (0 – 120 min) of 50 µgmL^{-1} of FCD-**3** at 37 °C relative to untreated cells.

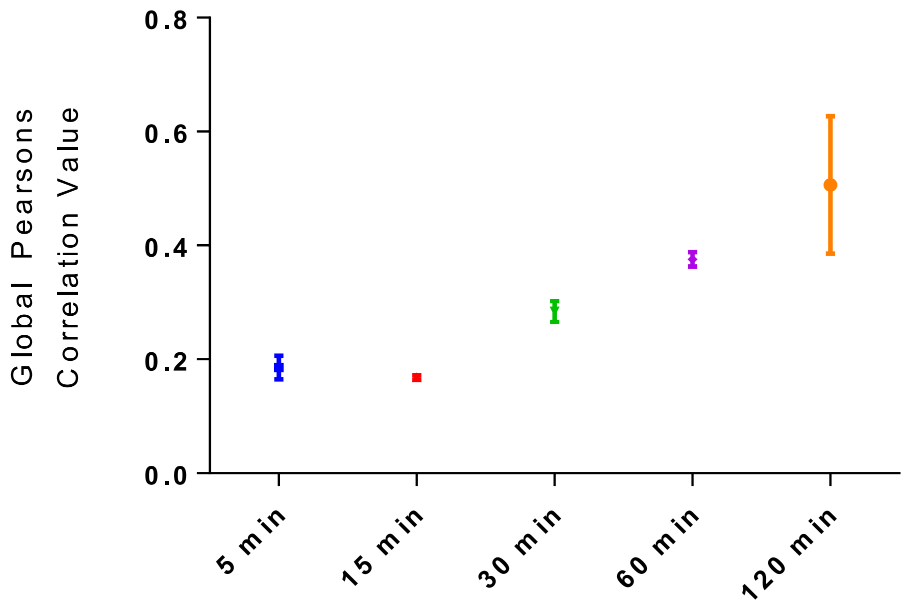

Figure S28. Global Pearson's Correlation (average) values for nuclear colocalization of HDF cells after exposure to 50 µgmL^{-1} of FCD-**3** for 5 min - 2 h 37°C. Accumulation in the nucleus is slow and gradual in HDF cells.

Representative Confocal Images

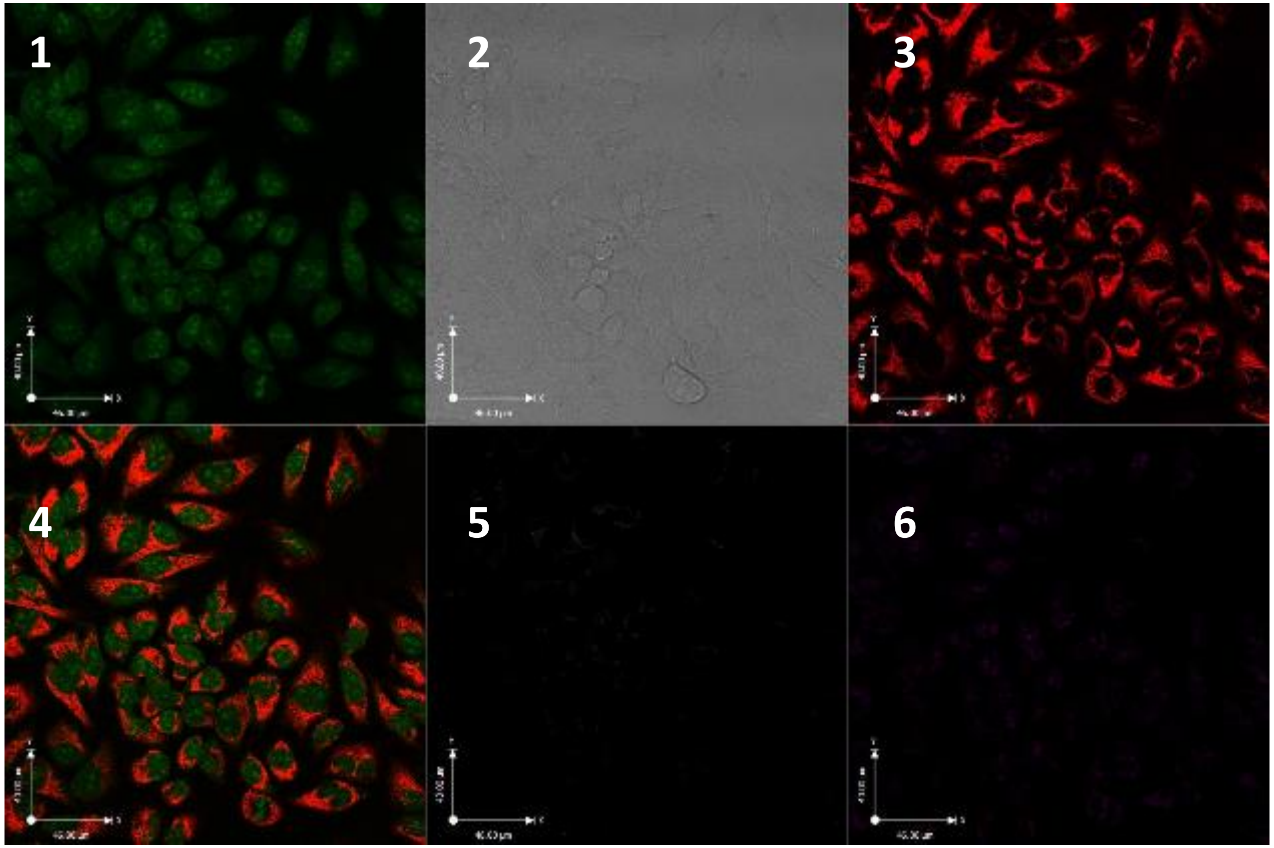

Top Row (1-3): 1: G-FCD; 2: Bright Field; 3: MitoTracker Red

Bottom Row (4-6): 4: 1/3 Overlay; 5: Positive Colocalisation; 6: Negative Colocalisation

Pearsons Correlation	Colocalisation Coefficient M1	Colocalisation Coefficient M2	Overlap Coefficient R	Overlap Coefficient k1	Overlap Coefficient k2	Ch. 1 Threshold	Ch. 2 Threshold	Voxel Ratio Ch.1/Ch.2	Global Pearsons Correlation
-0.0979	0.36	0.747	0.382	1.16	0.125	15	33	1.59	0.239

Figure S29. Confocal image illustrating the colocalization between FCD-**3** and a mitochondrial tracker in HeLa cells over 2 h.

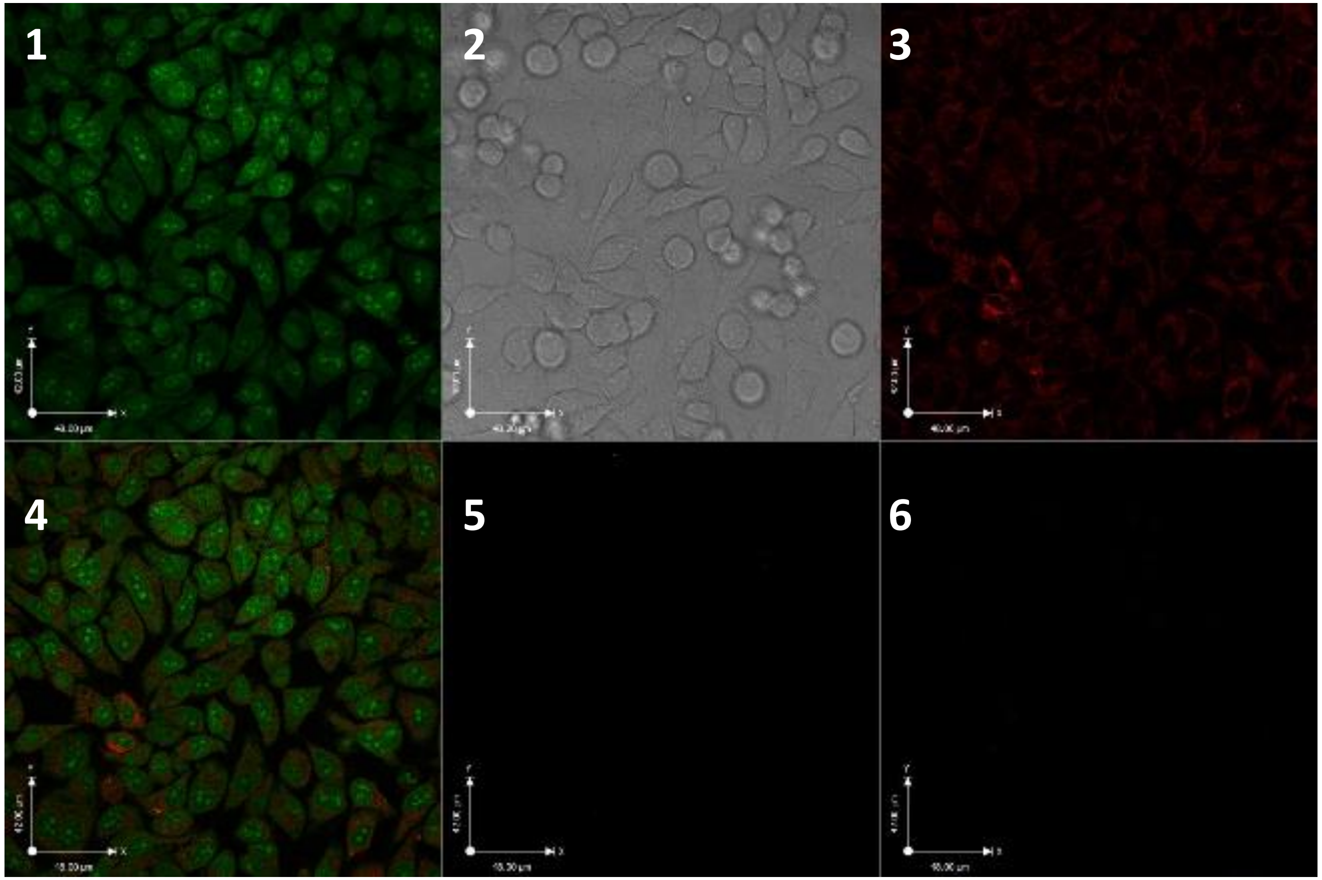

Top Row (1-3): 1: G-FCD; 2: Bright Field; 3: Late Endosome Tracker

Bottom Row (4-6): 4: 1/3 Overlay; 5: Positive Colocalisation; 6: Negative Colocalisation

Pearsons Correlation	Colocalisation Coefficient M1	Colocalisation Coefficient M2	Overlap Coefficient R	Overlap Coefficient k1	Overlap Coefficient k2	Ch. 1 Threshold	Ch. 2 Threshold	Voxel Ratio Ch.1/Ch.2	Global Pearsons Correlation
-0.0915	0.275	0.363	0.256	0.204	0.322	35	22	1.27	0.141

Figure S30. Confocal image illustrating the colocalization between FCD-**3** and late endosomal trackers in HeLa cells after 2h.

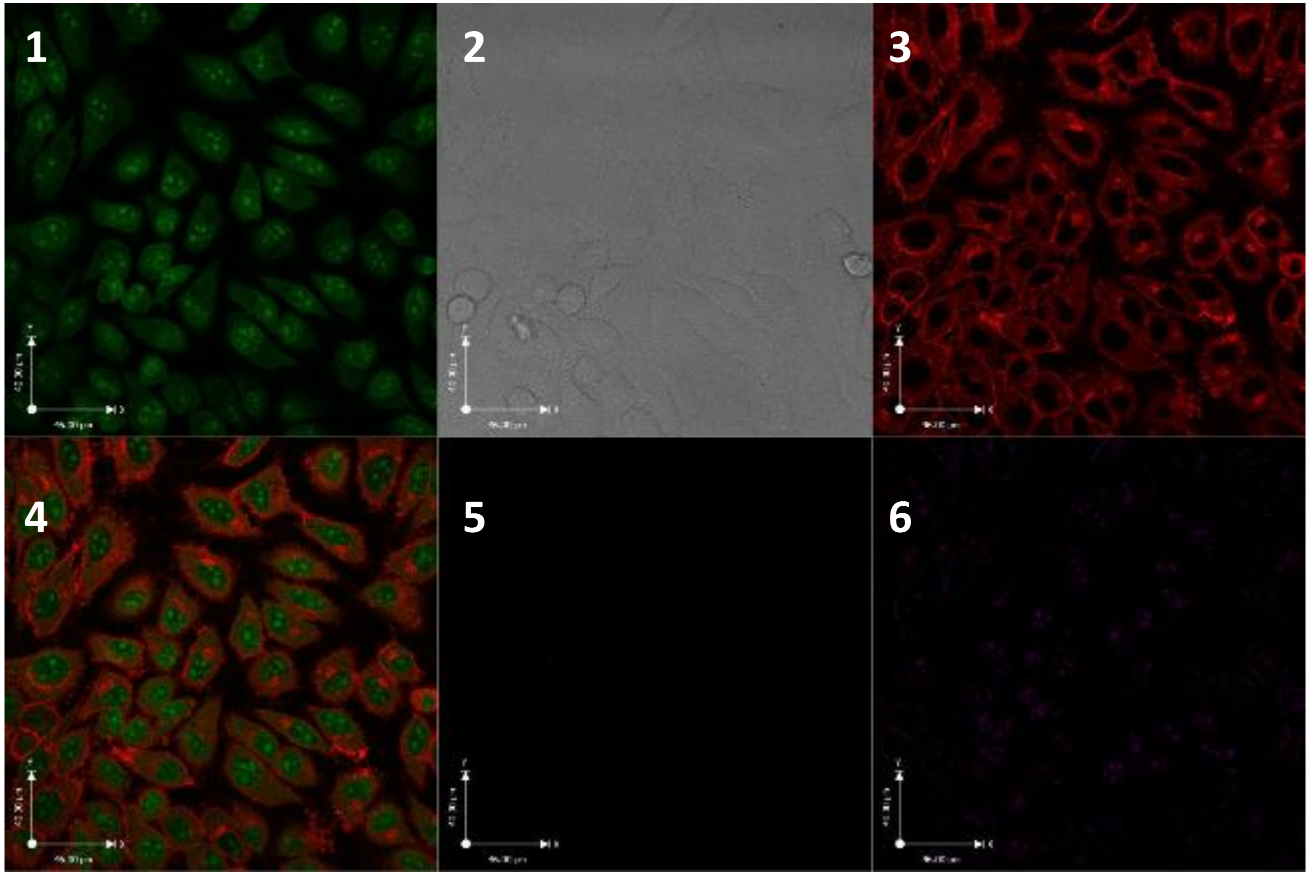

Top Row (1-3): 1: G-FCD; 2: Bright Field; 3: Golgi Tracker

Bottom Row (4-6): 4: 1/3 Overlay; 5: Positive Colocalisation; 6: Negative Colocalisation

Pearsons Correlation	Colocalisation Coefficient M1	Colocalisation Coefficient M2	Overlap Coefficient R	Overlap Coefficient k1	Overlap Coefficient k2	Ch. 1 Threshold	Ch. 2 Threshold	Voxel Ratio Ch.1/Ch.2	Global Pearsons Correlation
-0.0608	0.0636	0.0665	0.0509	0.105	0.0248	35	71	1	0.0298

Figure S31. Confocal image illustrating the colocalization between FCD-**3** and a Golgi tracker in HeLa cells after 2h.

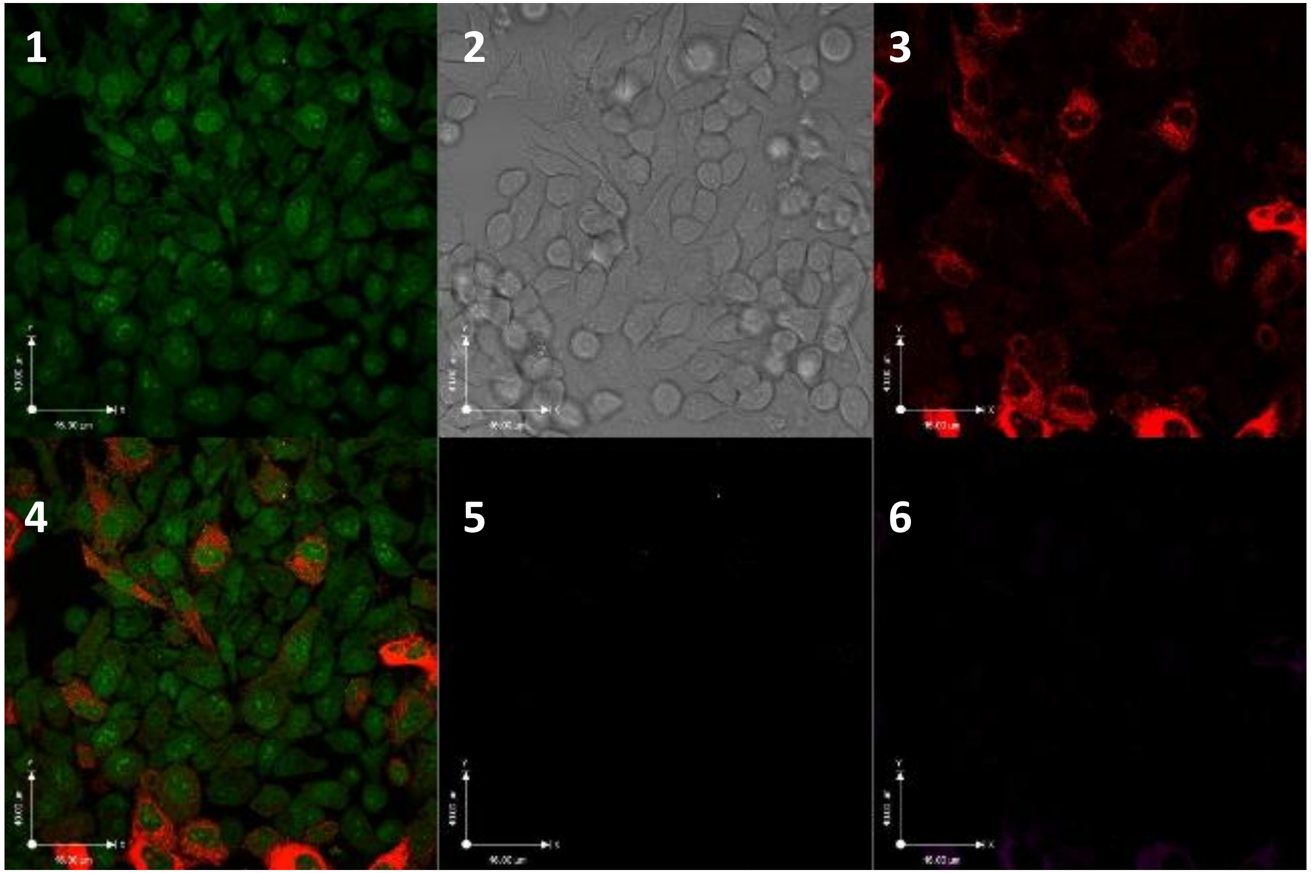

Top Row (1-3): 1: G-FCD; 2: Bright Field; 3: Early Endosome Tracker

Bottom Row (4-6): 4: 1/3 Overlay; 5: Positive Colocalisation; 6: Negative Colocalisation

Pearsons Correlation	Colocalisation Coefficient M1	Colocalisation Coefficient M2	Overlap Coefficient R	Overlap Coefficient k1	Overlap Coefficient k2	Ch. 1 Threshold	Ch. 2 Threshold	Voxel Ratio Ch.1/Ch.2	Global Pearsons Correlation
-0.0828	0.102	0.184	0.107	0.173	0.0666	43	56	1.99	0.0671

Figure S32. Confocal image illustrating the colocalization between FCD-**3** and an early endosomal tracker in HeLa cells after 2h.

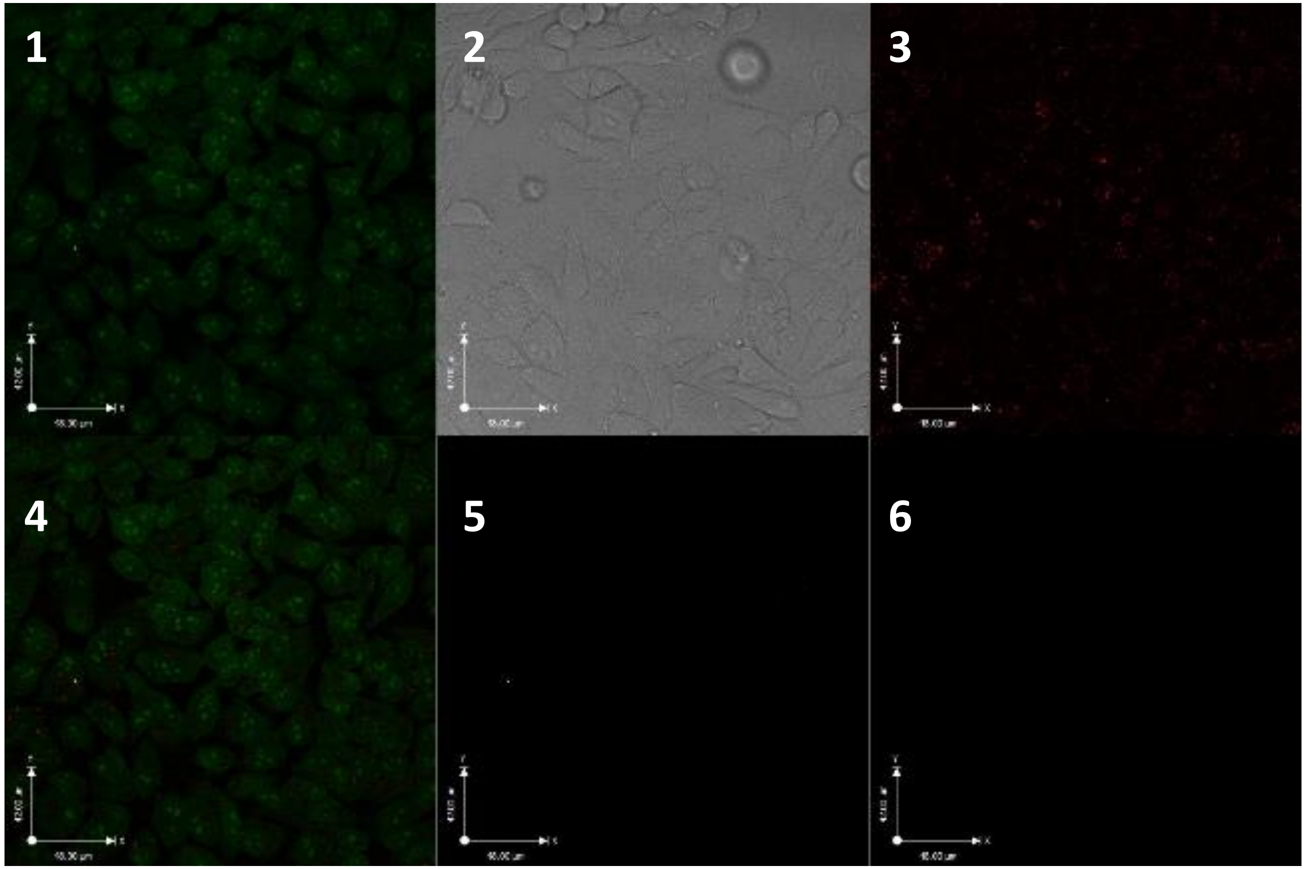

Top Row (1-3): 1: G-FCD; 2: Bright Field; 3: Lysosome Tracker

Bottom Row (4-6): 4: 1/3 Overlay; 5: Positive Colocalisation; 6: Negative Colocalisation

Pearsons Correlation	Colocalisation Coefficient M1	Colocalisation Coefficient M2	Overlap Coefficient R	Overlap Coefficient k1	Overlap Coefficient k2	Ch. 1 Threshold	Ch. 2 Threshold	Voxel Ratio Ch.1/Ch.2	Global Pearsons Correlation
0.461	0.0371	0.0481	0.0521	0.0388	0.07	38	15	1.04	0.038

Figure S33. Confocal image illustrating the colocalization between FCD-**3** and a lysosomal tracker in HeLa cells after 2h.

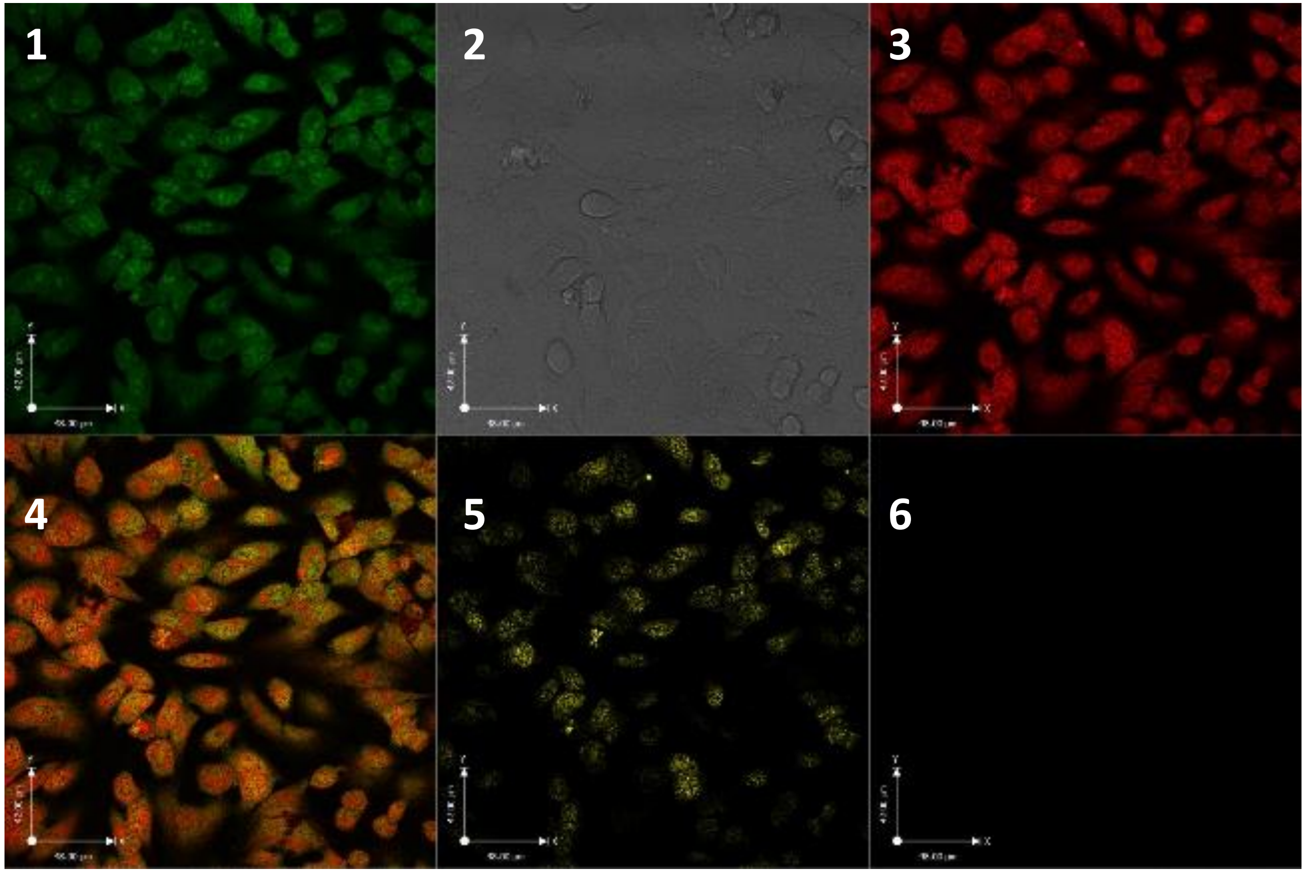

Top Row (1-3): 1: G-FCD; 2: Bright Field; 3: Nuclear (Red647) Tracker

Bottom Row (4-6): 4: 1/3 Overlay; 5: Positive Colocalisation; 6: Negative Colocalisation

Pearsons Correlation	Colocalisation Coefficient M1	Colocalisation Coefficient M2	Overlap Coefficient R	Overlap Coefficient k1	Overlap Coefficient k2	Ch. 1 Threshold	Ch. 2 Threshold	Voxel Ratio Ch.1/Ch.2	Global Pearsons Correlation
0.743	1	0.999	0.873	1.72	0.444	1	1	0.991	0.745

Figure S34. Confocal image illustrating colocalization of FCD-**3** in the nucleus of HeLa cells after exposure to FCD-**3** (50 μgmL^{-1}) for 2 h.

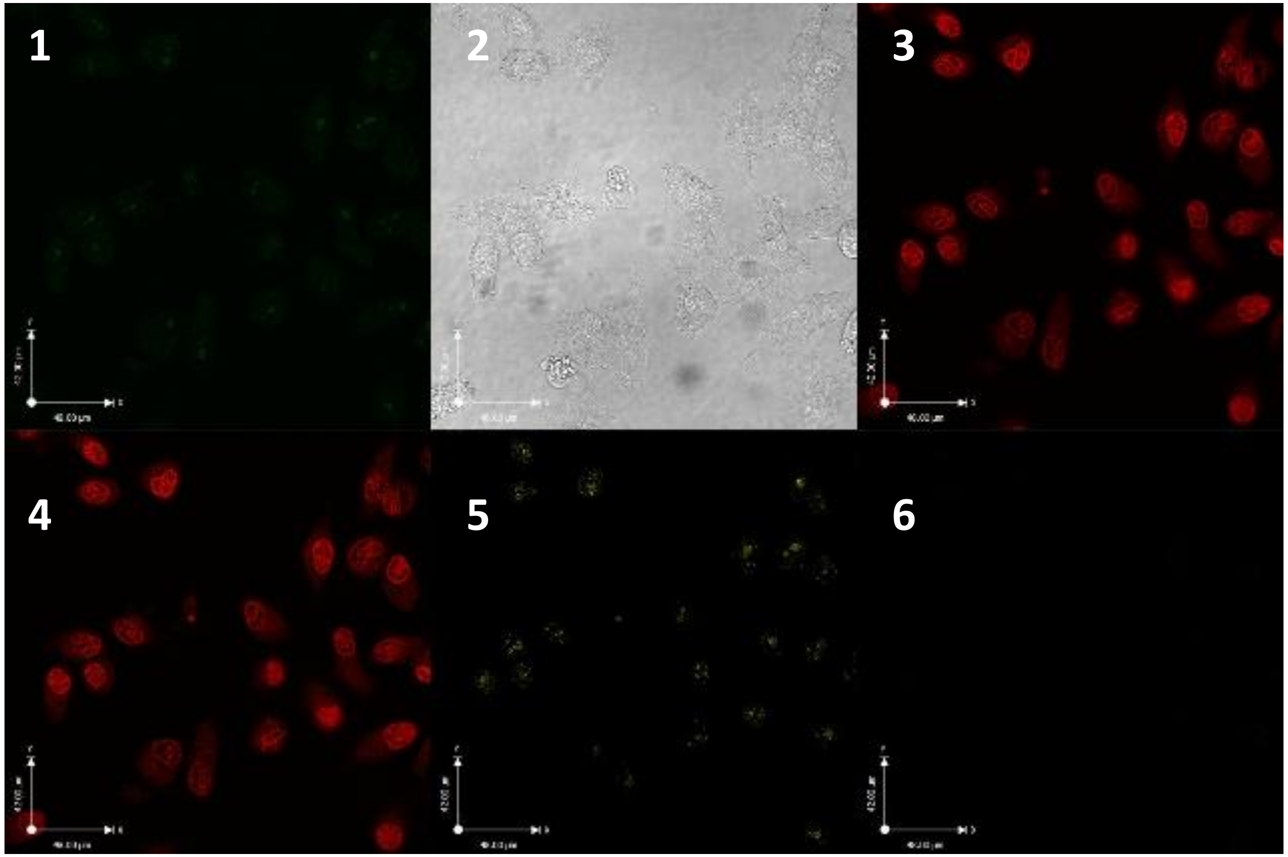

Top Row (1-3): 1: G-FCD; 2: Bright Field; 3: Nuclear (Red647) Tracker

Bottom Row (4-6): 4: 1/3 Overlay; 5: Positive Colocalisation; 6: Negative Colocalisation

Pearsons Correlation	Colocalisation Coefficient M1	Colocalisation Coefficient M2	Overlap Coefficient R	Overlap Coefficient k1	Overlap Coefficient k2	Ch. 1 Threshold	Ch. 2 Threshold	Voxel Ratio Ch.1/Ch.2	Global Pearsons Correlation
0.106	0.647	0.530	0.501	9.11	0.0275	2	23	0.875	0.475

Figure S35. Confocal image illustrating the colocalization between FCD-**3** and the nucleus tracker in HeLa cells after 1 min exposure

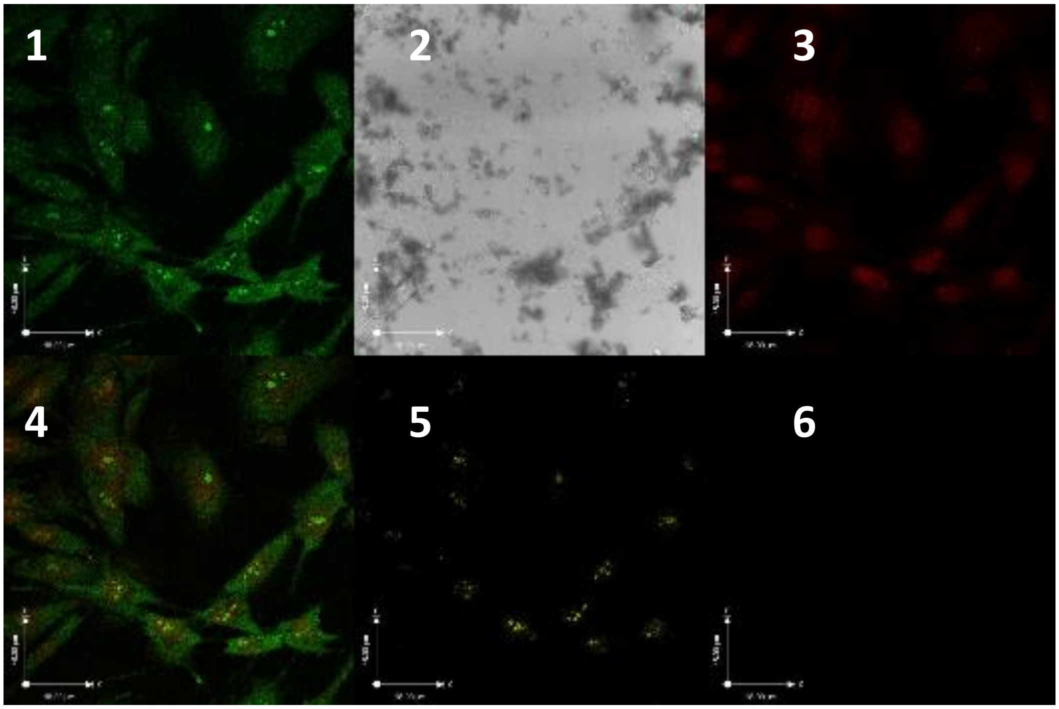

Figure S36. Confocal fluorescence images of FCD-**3** exposed HDF (500 µgmL^{-1}, 2 h) 1: G-FCD; 2: Nuclear (Red647) Tracker; 3: Bright Field; 4: 1/3 Overlay; 5: Positive Colocalisation; 6: Negative Colocalisation

FRET Studies to determine interactions between FCD-3 and intracellular RNA and/or DNA

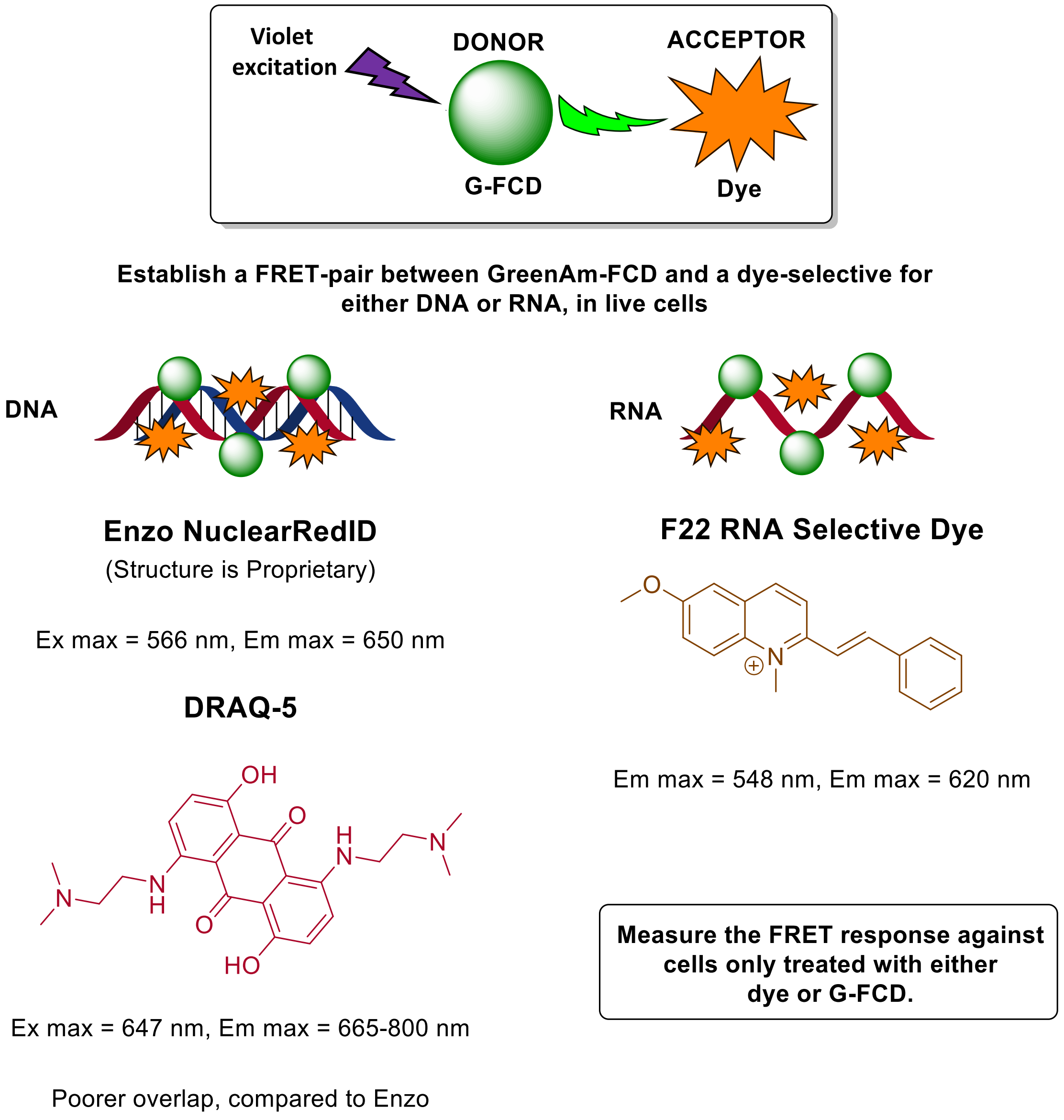

Figure S37. Experimental outline for the investigating the FCD-**3** / polynucleotide binding using **3** as a FRET-donor and a FRET-acceptor pair (A) DNA intercalators: Enzo NuclearRedID or DRAQ-5 and B) RNA intercalator F22 RNA Selective Dye.

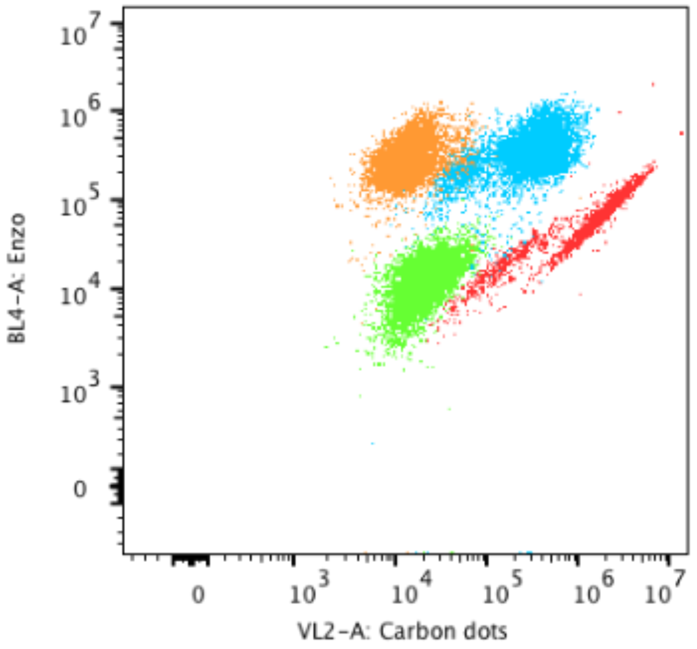

	Sample	Median BL4-A	Median VL2-A
	Untreated cells	12353	21250
	NuclearRed-ID	255992	13038
	G-FCD + NuclearRed-ID	321798	328563
	G-FCD	64611	1.80e6

Figure S38. Fluorescence of FCD-**3** and Enzo NuclearRed-ID DNA marker in HeLa cells

Change in fluorescence for FCD-**3** (using VL2-A detector)

$$Change\ in\ fluorescence\ median\ value = \frac{(G - FCD + NuclearRed - ID)}{(G - FCD)}$$

$$= \frac{328563}{1.80e6} = 0.182$$

FCD-**3** median fluorescence intensity (MFI) was diminished by 82% in the presence of Enzo NuclearRed-ID DNA binding dye

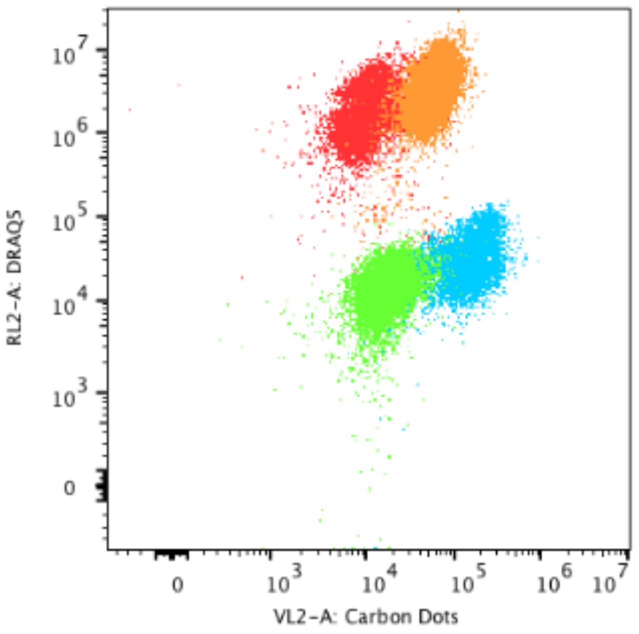

	Sample	Median RL2-A	Median VL2-A
	Untreated cells	14396	15676
	G-FCD + DRAQ5	2.82e6	55742
	G-FCD	28964	136262
	DRAQ5	1.41e6	9682

Figure S39. Fluorescence of FCD-**3** and DRAQ-5 DNA stain in HeLa cells

Change in fluorescence for FCD-**3** (using VL2-A detector)

$$Change\ in\ fluorescence\ median\ value = \frac{(G - FCD + DRAQ5)}{(G - FCD)}$$

$$= \frac{55742}{136262} = 0.409$$

FCD-**3** MFI was diminished by 60% in the presence of DRAQ-5 DNA binding dye.

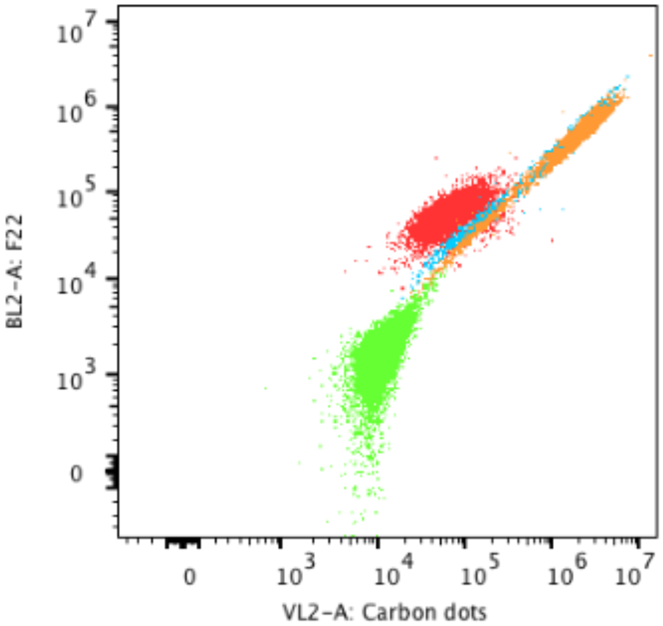

	Sample	Median BL2-A	Median VL2-A
	Untreated cells	1869	11745
	G-FCD	385379	1.80e6
	G-FCD + F22	397597	1.73e6
	F22	55901	65962

Figure S40. Fluorescence of FCD-**3** and F22 RNA stain in HeLa cells

Change in fluorescence for FCD-**3** (using VL2-A detector)

$$Change\ in\ fluorescence\ median\ value = \frac{(G - FCD + F22)}{(G - FCD)}$$

$$= \frac{1.73e6}{1.80e6} = 0.961$$

FCD-**3** MFI was diminished by 4% in the presence of F22 RNA binding dye; deemed to be within error.

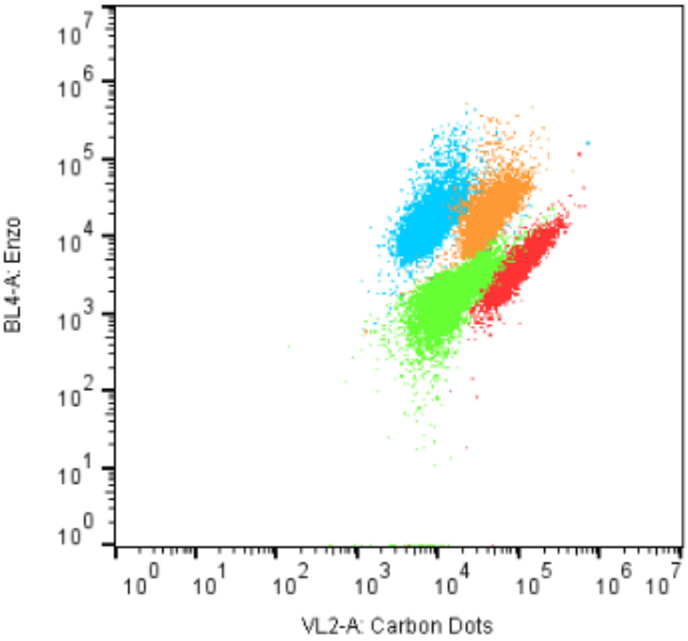

	Sample	Median BL4-A	Median VL2-A
	Untreated cells	1698	13039
	G-FCD + NuclearRed-ID	17309	40497
	NuclearRed-ID	14500	7787
	G-FCD	3644	79683

Figure S41. Fluorescence of FCD-**3** and NuclearRed-ID DNA marker in HDF cells

Change in fluorescence for FCD-**3** (using VL2-A detector)

$$Change\ in\ fluorescence\ median\ value = \frac{(G - FCD + NuclearRed - ID)}{(G - FCD)}$$

$$= \frac{40497}{79683} = 0.508$$

FCD-**3** MFI was diminished by 50% in the presence of NuclearRed-ID DNA binding dye

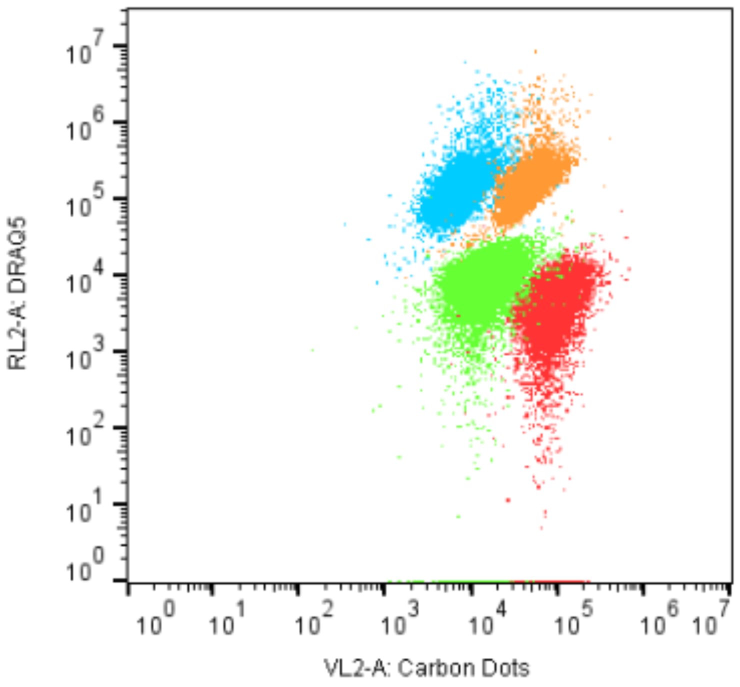

	Sample	Median RL2-A	Median VL2-A
(green)	Untreated cells	8592	13039
(orange)	G-FCD + DRAQ-5	127273	44860
(blue)	DRAQ-5	117178	7169
(red)	G-FCD	4488	79683

Figure S42. Fluorescence of G-FCD and DRAQ-5 DNA stain in HDF cells

Change in fluorescence for G-FCD (using VL2-A detector)

$$Change\ in\ fluorescence\ median\ value = \frac{(G - FCD + DRAQ5)}{(G - FCD)}$$

$$= \frac{44860}{79683} = 0.562$$

FCD-**3** MFI was diminished by 44% in the presence of DRAQ-5 DNA binding dye

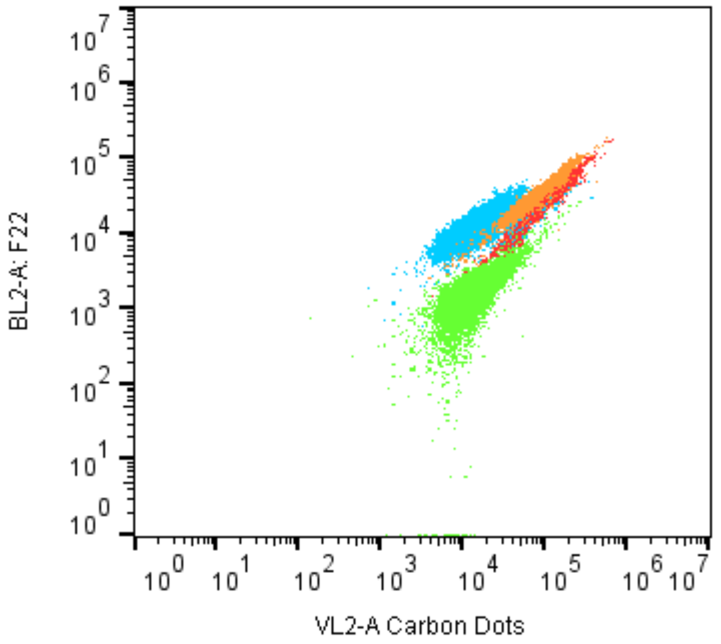

	Sample	Median BL2-A	Median VL2-A
	Untreated cells	1582	13039
	G-FCD + F22	30147	74234
	F22	11541	13777
	G-FCD	20260	79683

Figure S43. Fluorescence of G-FCD and F22 RNA stain in HDF cells

Change in fluorescence for G-FCD (using VL2-A detector)

$$Change\ in\ fluorescence\ median\ value = \frac{(G - FCD + F22)}{(G - FCD)}$$

$$= \frac{74234}{79683} = 0.931$$

FCD-**3** MFI was diminished by 7% in the presence of F22 RNA binding dye; deemed to be within error.

Cell Toxicity assays

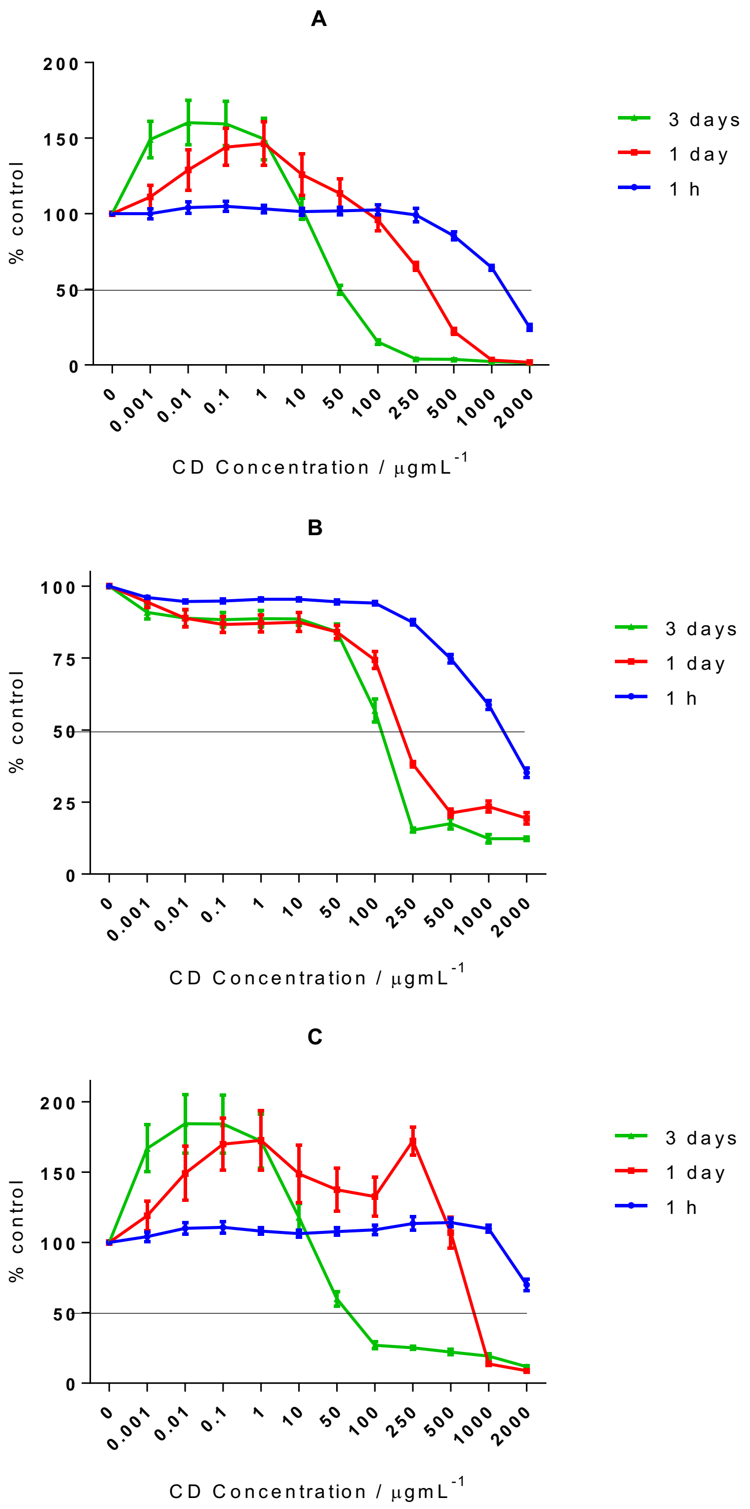

Figure S44. HeLa cells exposure to FCD-**3** for 1 h, 1 and 3 days – (A) reductive metabolism (RM); (B) viable cell numbers and (C) reductive metabolism per cell, all compared to untreated cells.

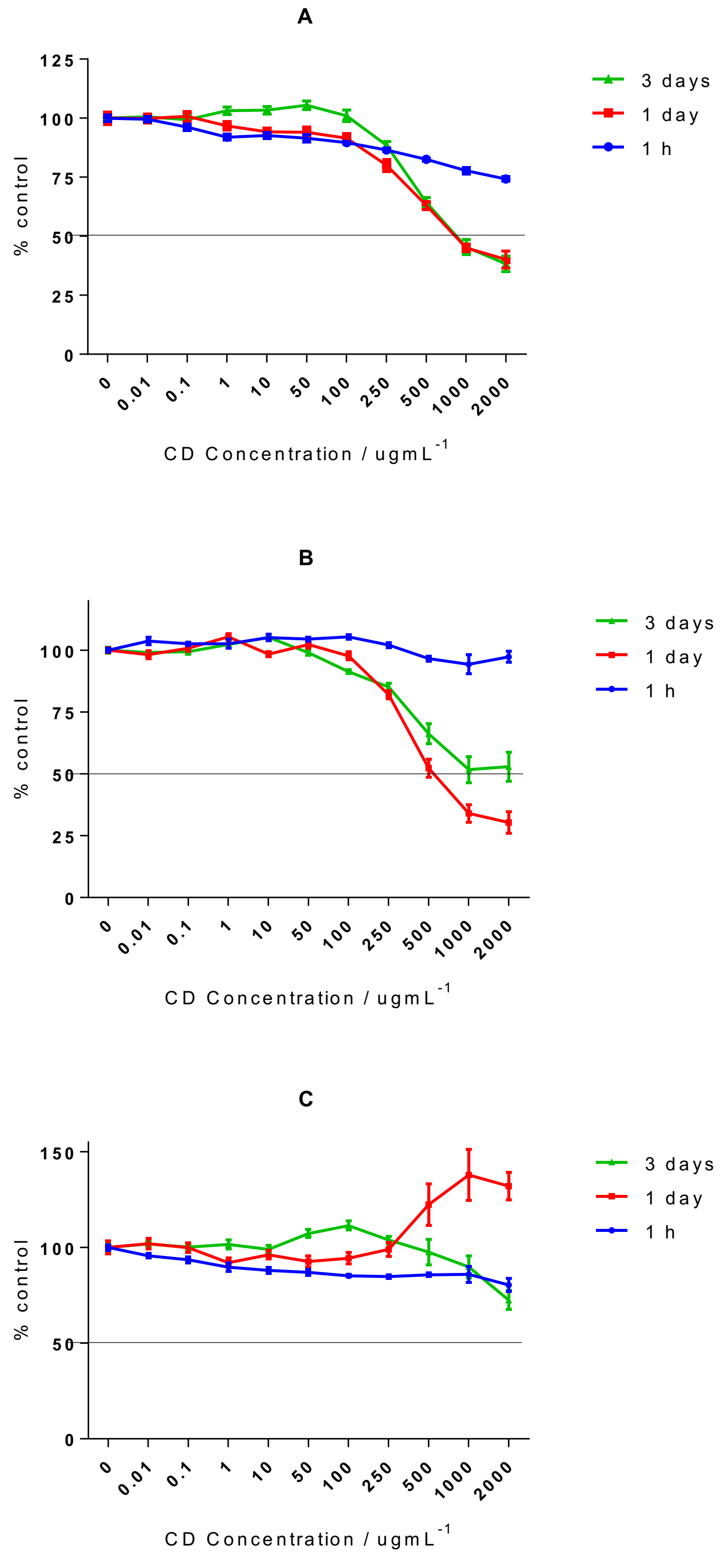

Figure S45. HDF cells exposure to FCD-**3** for 1 h, 1 and 3 days – (A) reductive metabolism (RM); (B) viable cell numbers and (C) reductive metabolism per cell, all compared to untreated cells *NB the apparent increase in reductive metabolism/cell at 3 days is a numerical artefact due to the reduced number of live cells*

Blue-LED Illumination

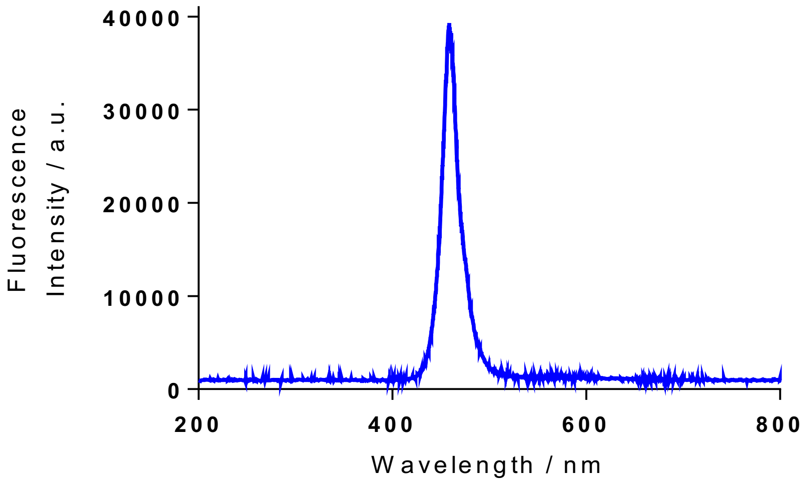

Figure S46. Emission profile of LEDs (blue-LED)

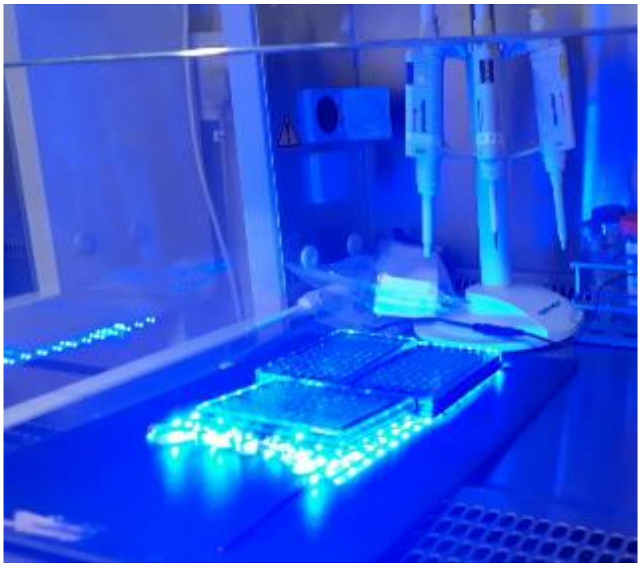

Figure S47. Image of the blue-LED illuminated cell-based experimental setup.

A)

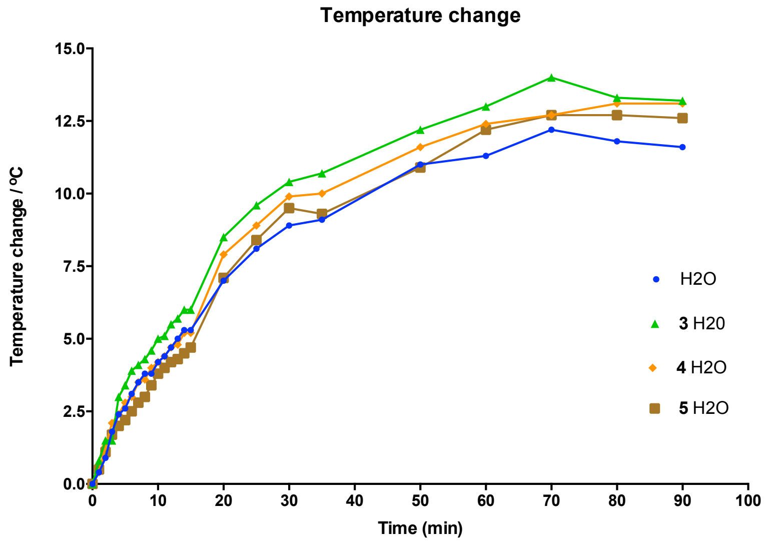

B)

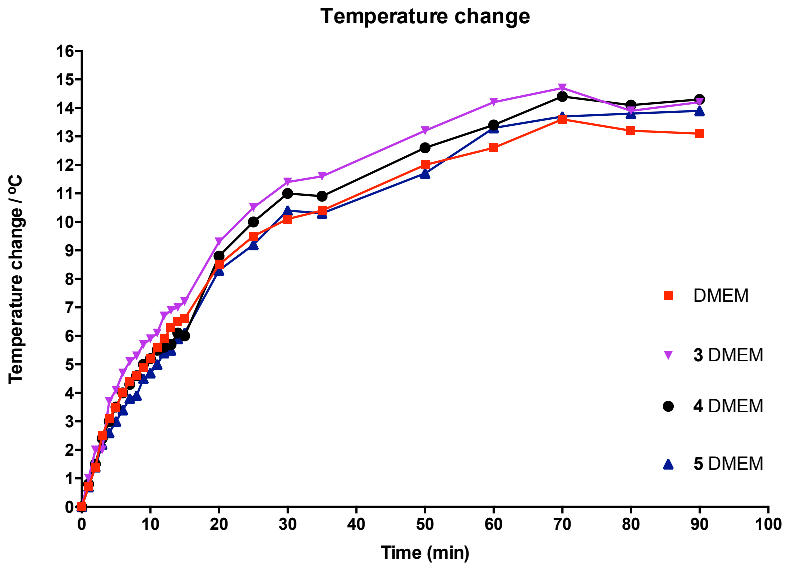

Figure S48. Temperature increases in A) deionised H_2O and B) DMEM cell culture medium after exposure to 90 min. blue-LED irradiation with and without FCD-**3** (500 µgmL^{-1}), FCD-core **5** (500 µgmL-1) and **4** (238 µgmL-1 (which is the same amount of **4** found in 500 µgmL^{-1} of **3**)). Note the excess heating and steeper initial slope of temperature increase in the presence of FCD-**3** when compared to water or media alone. Measurements were made in triplicate, average shown.

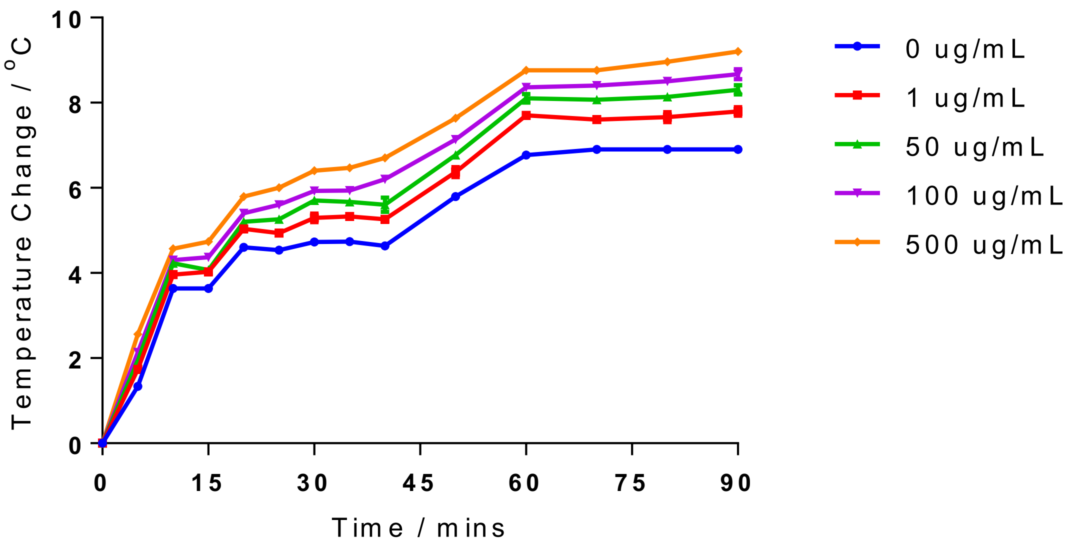

Figure S49. Temperature increases measured overtime in cell culture medium in 96-well plate with FCD-**3** treated HeLa cells (at a range of concentrations in water) after exposure to blue-LED irradiation at room temperature for 90 min. Measurements were made in triplicate, average shown.

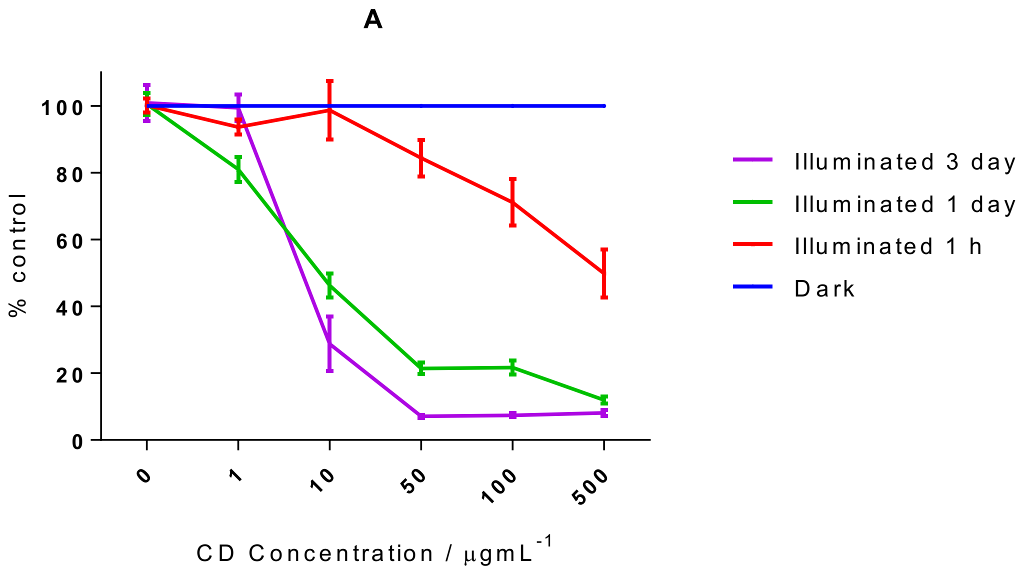

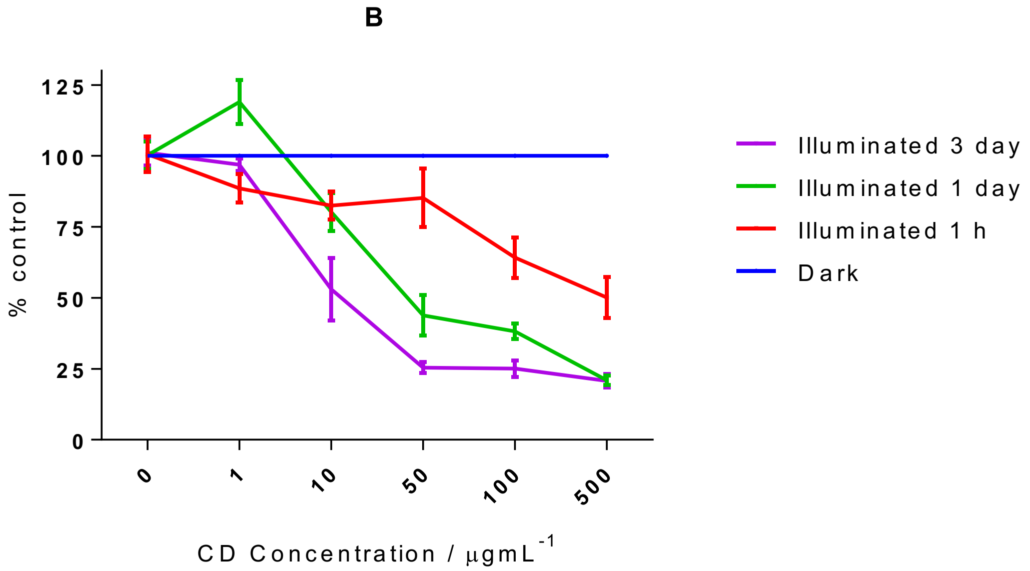

Figure S50. HeLa 2 h exposure to FCD-**3** followed by 30 min. blue-LED illumination. Effects on cell physiology were then measured after 1h, 1 or 3 days in cells that after LED irradiation had the FCD-**3** containing medium removed and fresh medium added. (A) reductive metabolism and (B) viable cell numbers compared to non-irradiated (Dark) cultures exposed to the equal concentrations of **3**.

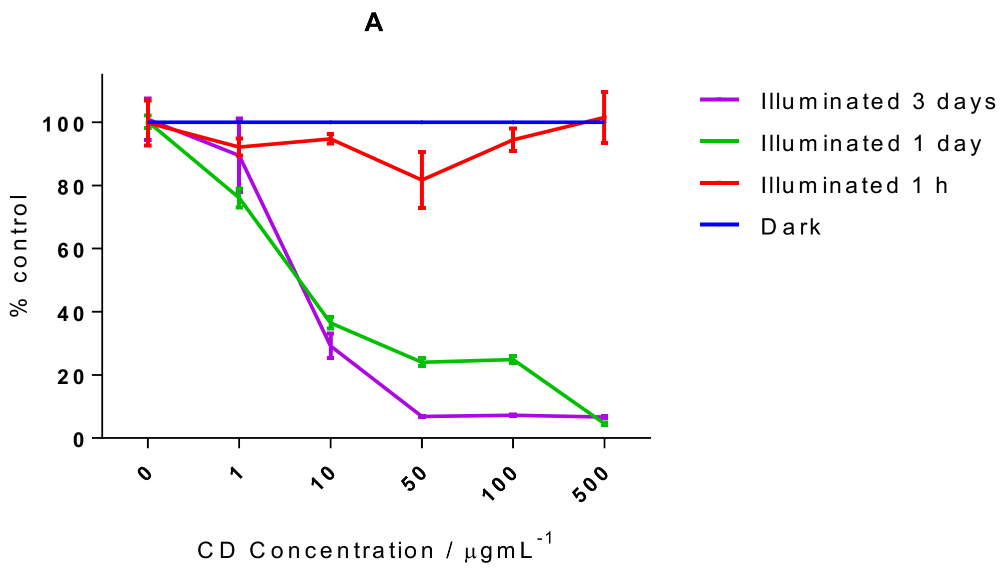

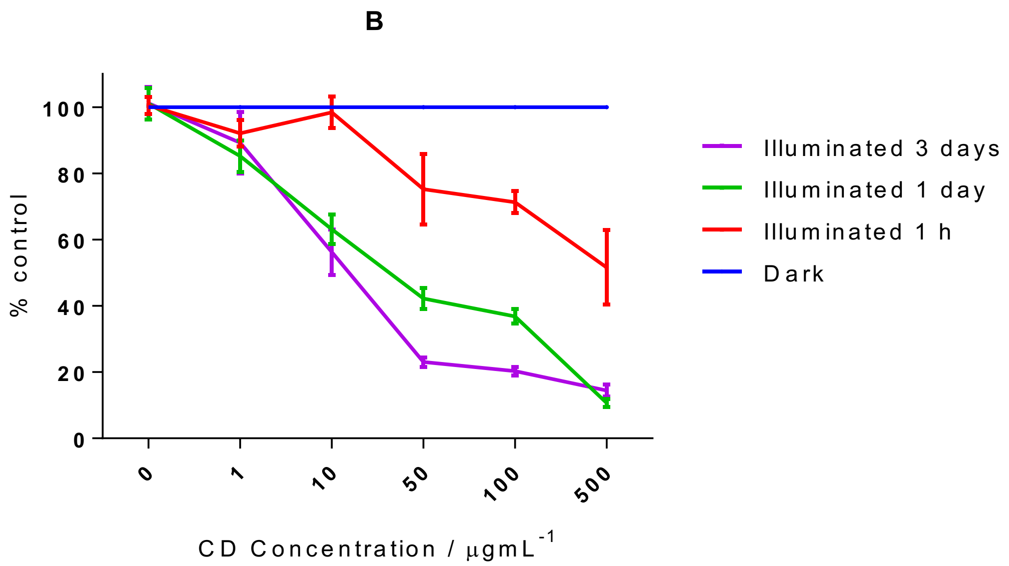

Figure S51. HeLa 2 h exposure to FCD-**3** followed by 60 min. blue-LED illumination. Effects on cell physiology were then measured after 1h, 1 or 3 days in cells that after LED irradiation had the FCD-**3** containing medium removed and fresh medium added. (A) reductive metabolism and (B) viable cell numbers compared to non-irradiated (Dark) cultures exposed to the equal concentrations of **3**.

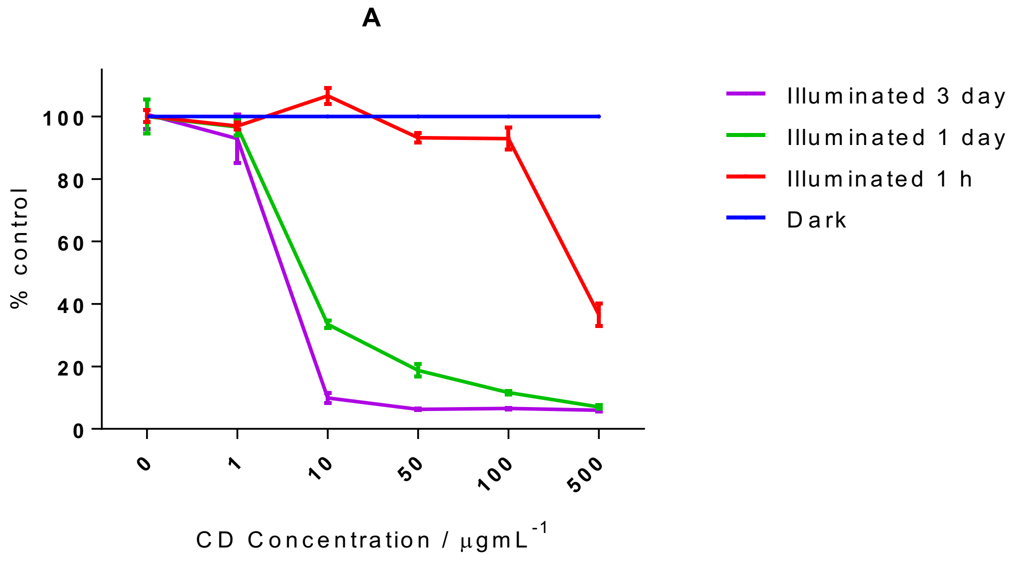

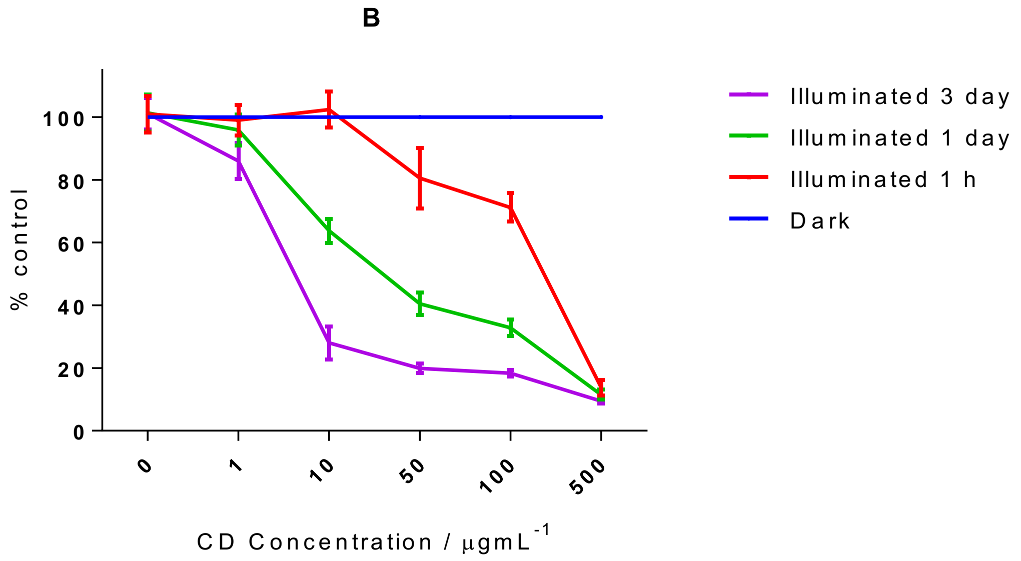

Figure S52. HeLa 2 h exposure to FCD-**3** followed by 90 min. blue-LED illumination. Effects on cell physiology were then measured after 1h, 1 or 3 days in cells that after LED irradiation had the FCD-**3** containing medium removed and fresh medium added. (A) reductive metabolism (Alamar Blue) and (B) viable cell numbers (Calcein AM) compared to non-irradiated (Dark) cultures exposed to the equal concentrations of **3**.

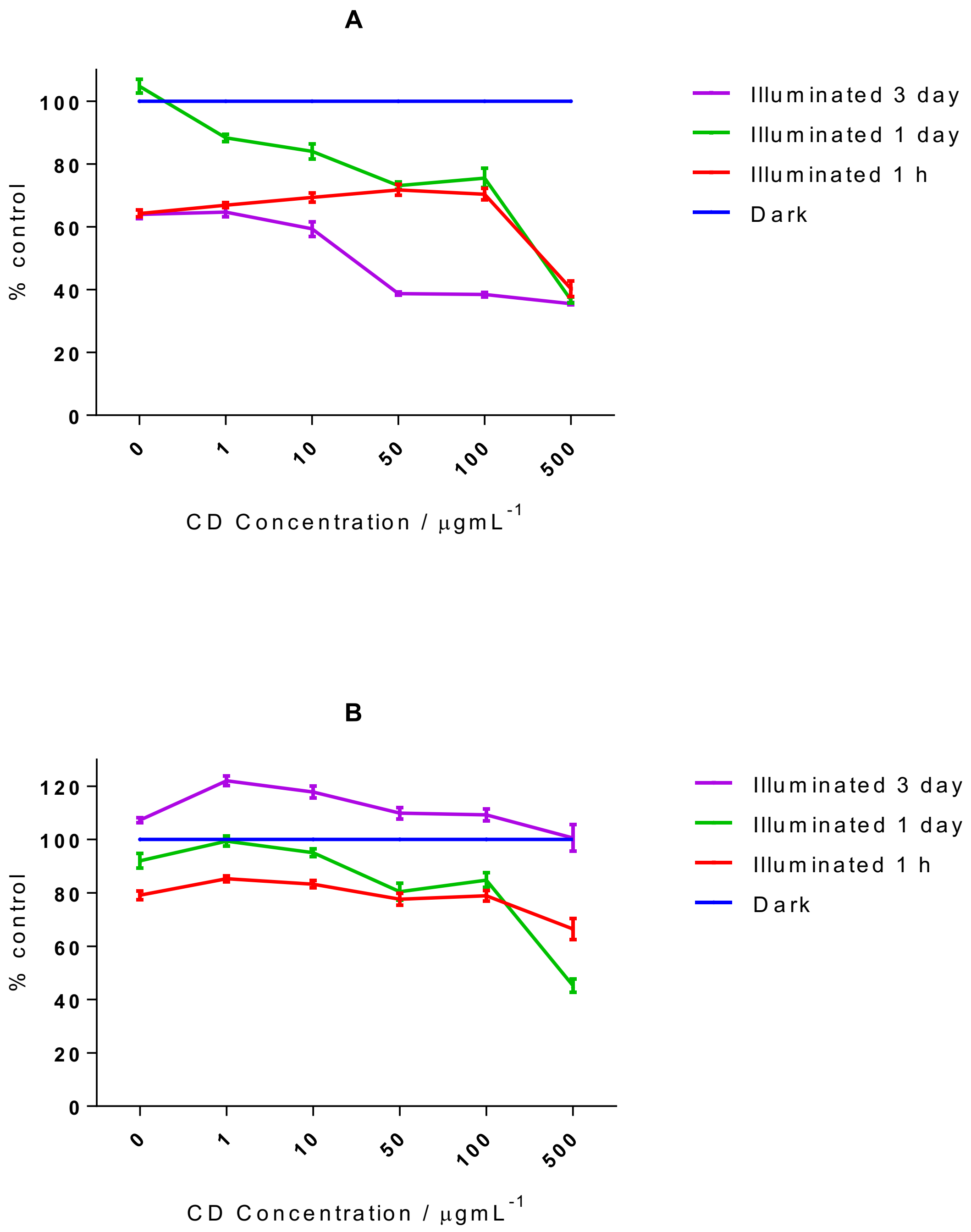

Figure S53. HDF 2 h exposure to FCD-**3** followed by 30 min. blue-LED illumination. Effects on cell physiology were then measured after 1h, 1 or 3 days in cells that after LED irradiation had the FCD-**3** containing medium removed and fresh medium added. (A) reductive metabolism (Alamar Blue) and (B) viable cell numbers (Calcein AM) compared to non-irradiated (Dark) cultures exposed to the equal concentrations of **3**.

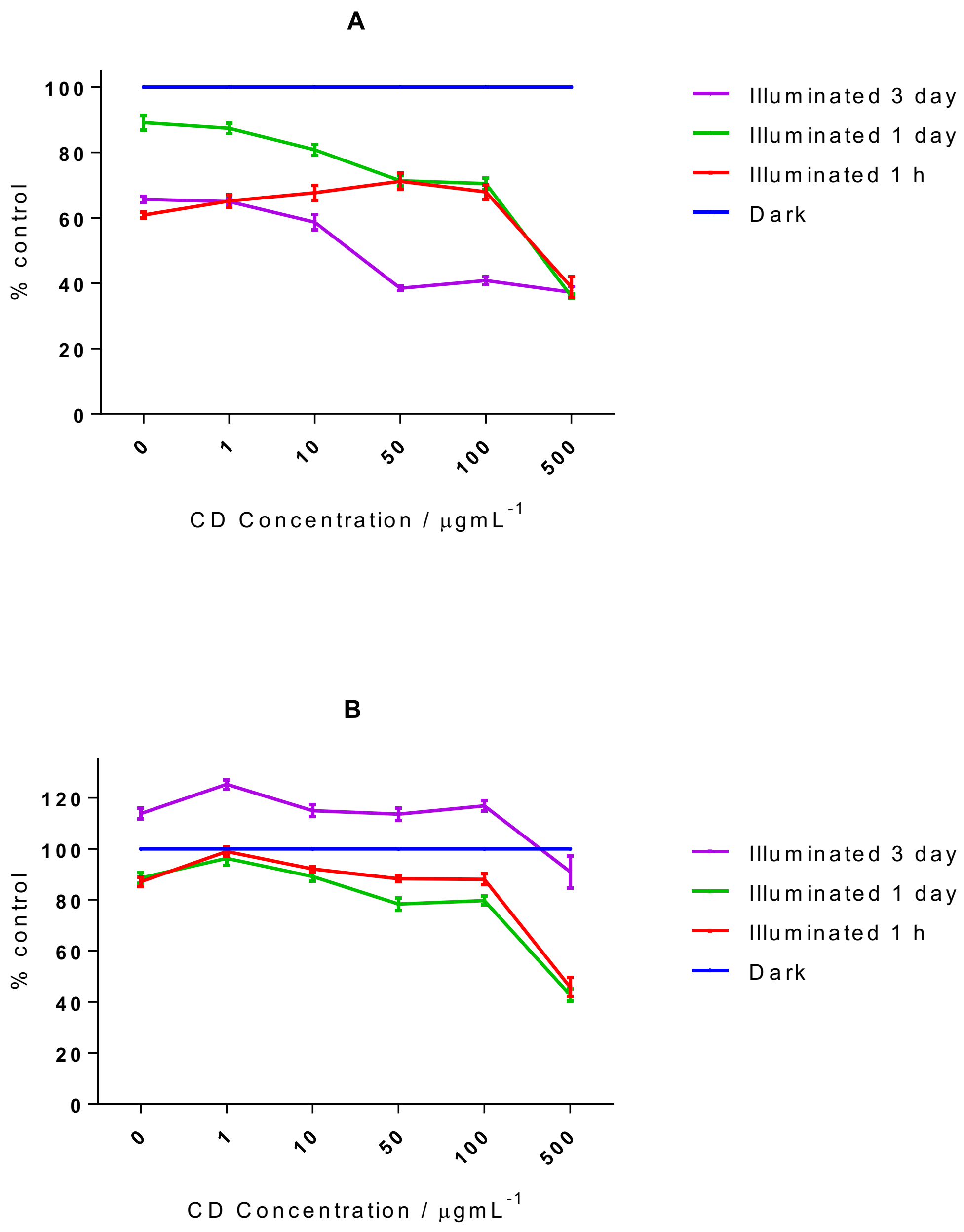

Figure S54. HDF 2 h exposure to FCD-**3** followed by 60 min. blue-LED illumination. Effects on cell physiology were then measured after 1h, 1 or 3 days in cells that after LED irradiation had the FCD-**3** containing medium removed and fresh medium added. (A) reductive metabolism (Alamar Blue) and (B) viable cell numbers (Calcein AM) compared to non-irradiated (Dark) cultures exposed to the equal concentrations of **3**.

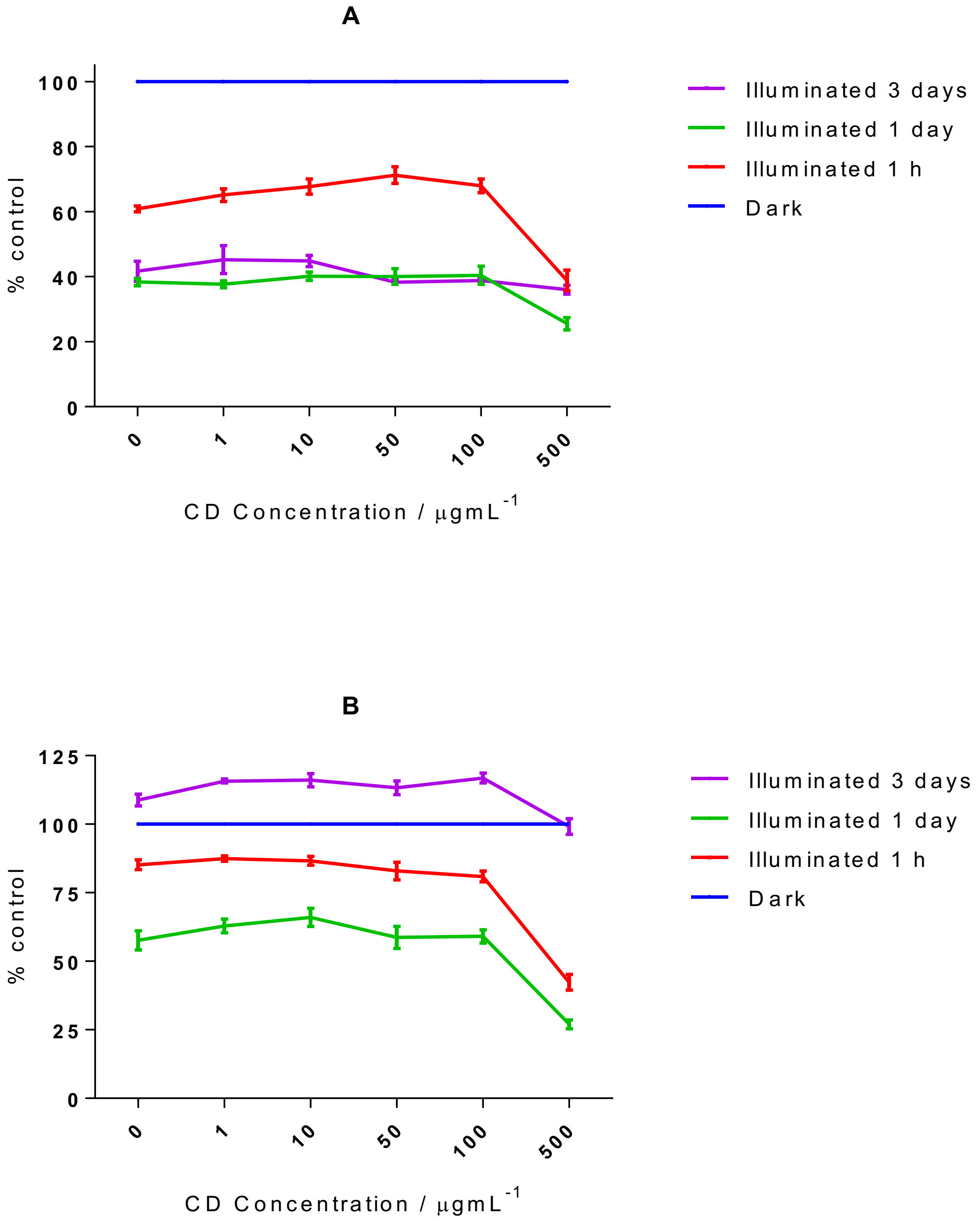

Figure S55. HDF 2 h exposure to FCD-**3** followed by 90 min. blue-LED illumination. Effects on cell physiology were then measured after 1h, 1 or 3 days in cells that after LED irradiation had the FCD-**3** containing medium removed and fresh medium added. (A) reductive metabolism (Alamar Blue) and (B) viable cell numbers (Calcein AM) compared to non-irradiated (Dark) cultures exposed to the equal concentrations of **3**.

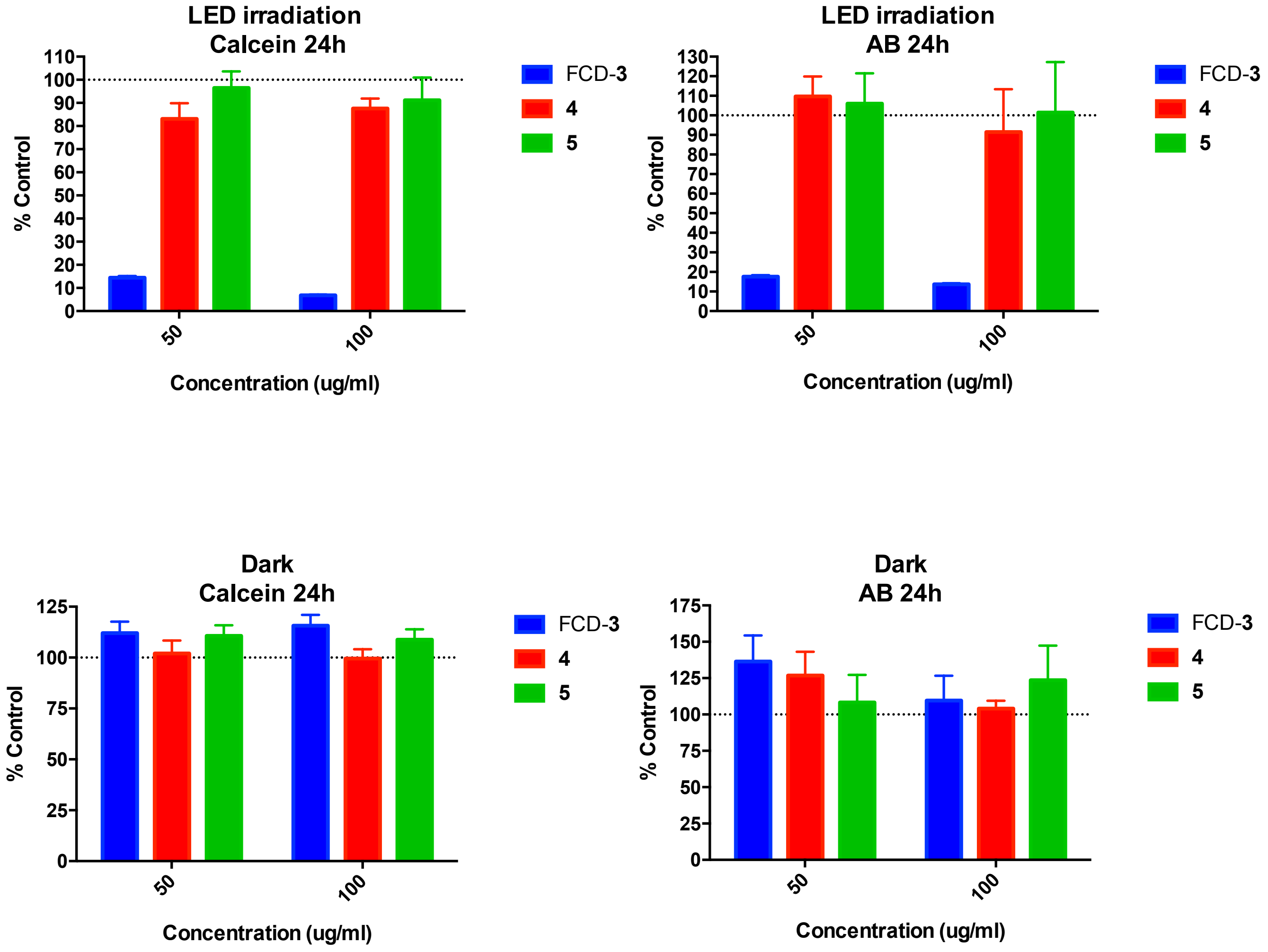

Figure S56. HeLa 2 h exposure to either FCD-**3**, 2,5-deoxyfructosazine **4 or** core FCD-**5** at two different concentrations (50 and 100 ug/mL), followed by 60 min. blue-LED illumination (top) or kept in the dark (bottom). Effects on cell physiology (viable cell numbers – Calcein AM) and reductive metabolism – Alamar Blue (AB)) were then measured after 1 day in cells that after LED irradiation had the FCD-**3** containing medium removed and fresh medium added.

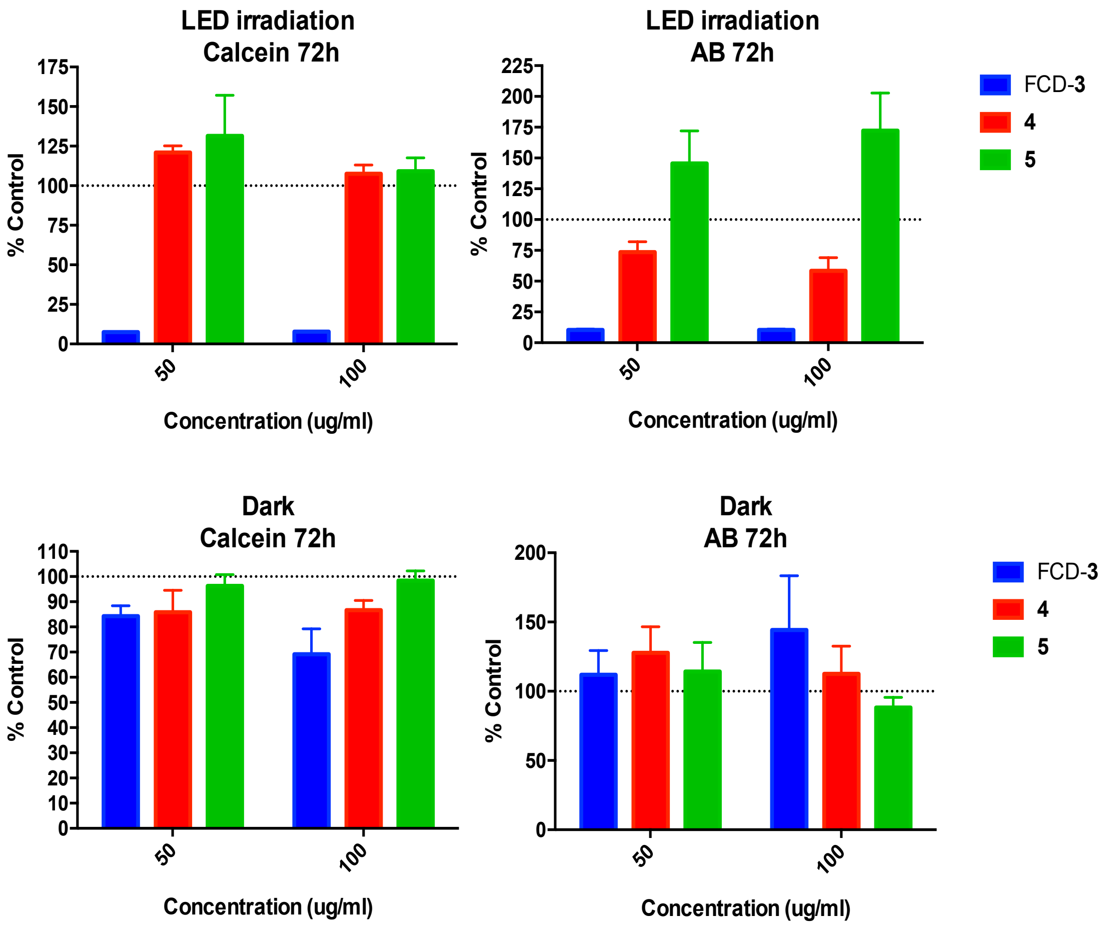

Figure S57. HeLa 2 h exposure to either FCD-**3**, 2,5-deoxyfructosazine **4** or core FCD-**5** at two different concentrations (50 and 100 ug/mL), followed by 60 min. blue-LED illumination (top) or kept in the dark (bottom). Effects on cell physiology (viable cell numbers – Calcein AM) and reductive metabolism – Alamar Blue (AB)) were then measured after 3 days in cells that after LED irradiation had the FCD-**3** containing medium removed and fresh medium added.

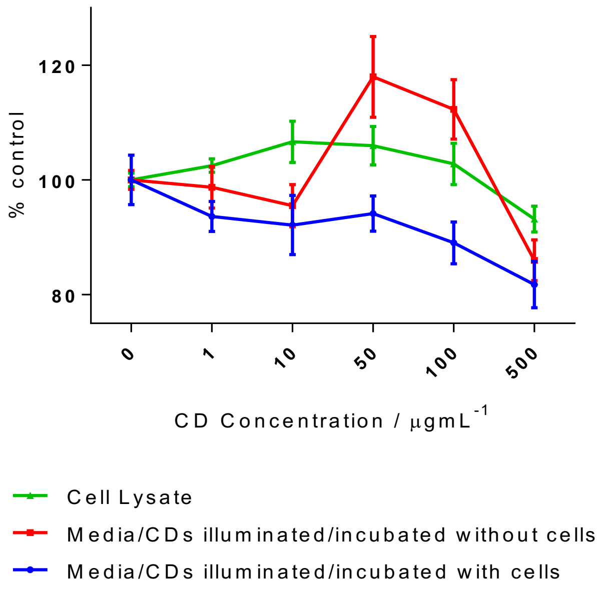

Figure S58. Secondary effects measured in the reductive metabolism of naïve HeLa cells that are treated with: cell lysates of cells that had been subjected to FCD-**3** exposure (2 h) at different concentrations followed by 90 min. LED irradiation (green); supernatants of of cells that had been subjected to FCD-**3** exposure (2 h) at different concentrations followed by LED irradiation (blue), and LED irradiated media containing **3** (red).

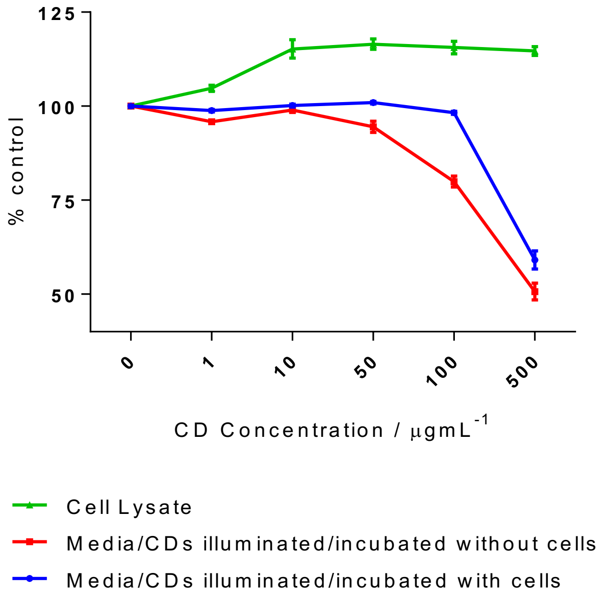

Figure S59. Secondary effects measured on the viable cell number in naïve HeLa cells that are treated with: cell lysates of cells that had been subjected to FCD-**3** exposure (2 h) at different concentrations followed by 90 min. LED irradiation (green); supernatants of of cells that had been subjected to FCD-**3** exposure (2 h) at different concentrations followed by LED irradiation (blue), and LED irradiated media containing **3** (red).

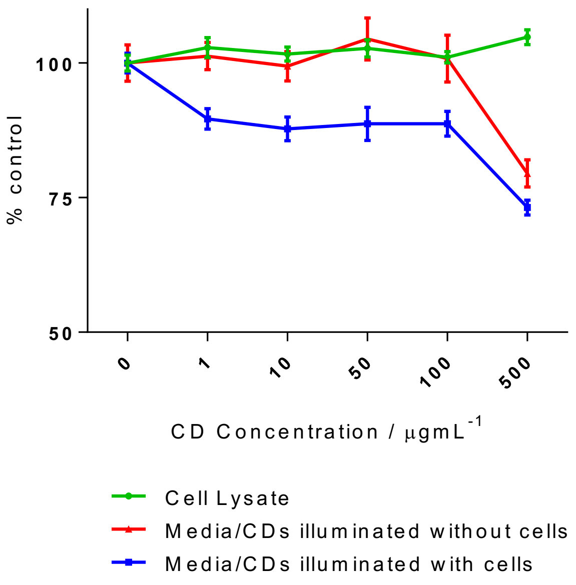

Figure S60. Secondary effects measured in the reductive metabolism of naïve HDF cells that are treated with: cell lysates of cells that had been subjected to FCD-**3** exposure (2 h) at different concentrations followed by 90 min. LED irradiation (green); supernatants of of cells that had been subjected to FCD-**3** exposure (2 h) at different concentrations followed by LED irradiation (blue), and LED irradiated media containing **3** (red).

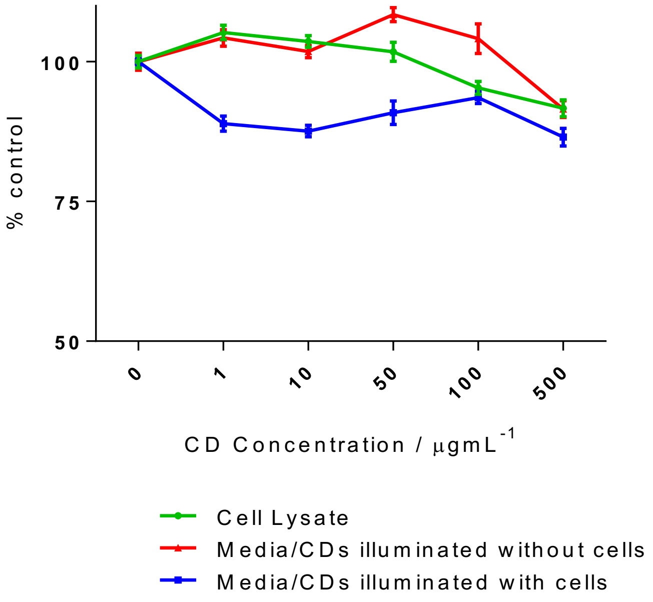

Figure S61. Secondary effects observed on the viable cell number in naïve HDF cells that are treated with: cell lysates of cells that had been subjected to FCD-**3** exposure (2 h) at different concentrations followed by 90 min. LED irradiation (green); supernatants of of cells that had been subjected to FCD-**3** exposure (2 h) at different concentrations followed by LED irradiation (blue), and LED irradiated media containing **3** (red).

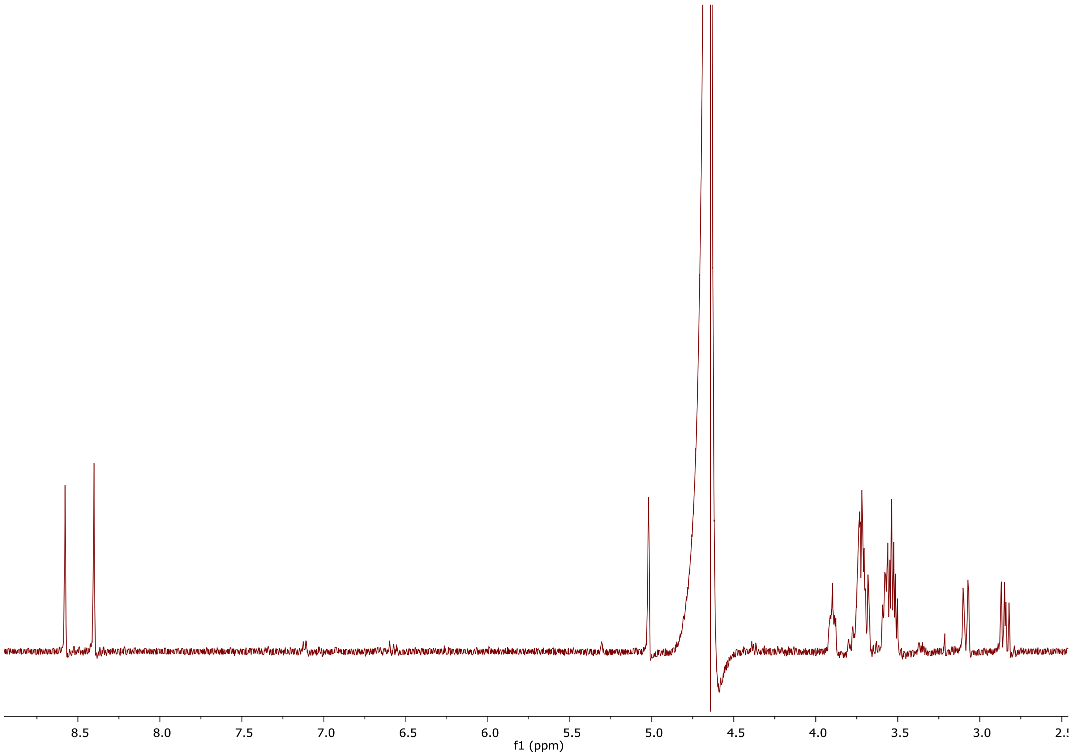

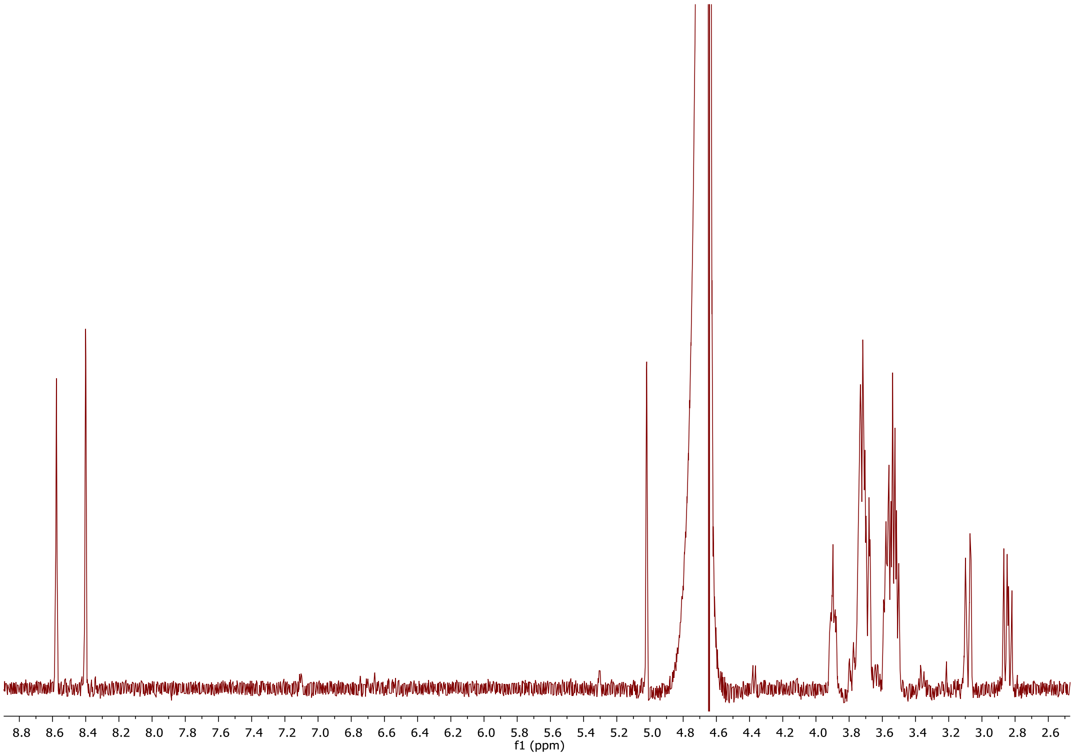

Figure S62. ^{1}H-NMR (500 MHz, D_2O) spectra of FCD-**3** before (Top) and after (Bottom) 90 min blue-LED illumination. No significant changes observed in peak placement, multiplicity or integration.

References

1. Rohovec, J.; Kotek, J.; Peters, J. A.; Maschmeyer, t., A Clean Conversion of D-Glucosamine Hydrochloride to a Pyrazine in the Presence of Phenylboronate or Borate. *Eur. J. Org. Chem.* **2001**, 3899-3901.
2. Jia, L.; Wang, Y.; Qiao, Y.; Qi, Y.; Hou, X., Efficient one-pot synthesis of deoxyfructosazine and fructosazine from d-glucosamine hydrochloride using a basic ionic liquid as a dual solvent-catalyst. *RSC Adv.* **2014,** *4* (83), 44253-44260.
3. Bhattacherjee, A.; Hrynets, Y.; Betti, M., Fructosazine, a Polyhydroxyalkylpyrazine with Antimicrobial Activity: Mechanism of Inhibition against Extremely Heat Resistant Escherichia coli. *J. Agric. Food Chem.* **2016,** *64* (45), 8530-8539.
4. Dunn, K. W.; Kamocka, M. M.; McDonald, J. H., A practical guide to evaluating colocalization in biological microscopy. *Am. J. Physiol. Cell Ph.* **2011,** *300* (4), C723-C742.
